# A polyherbal and probiotic-based immunostimulatory formulation for managing gut dysbiosis in colorectal cancer

**DOI:** 10.3389/fnut.2026.1854998

**Published:** 2026-09-09

**Authors:** Abhiram Kumar, Chhavi Dhiman, Vijaykumar Dayaram Nimbarte, Priyanka Erukulla, Madhu Rani Bharati, Raktim Chattopadhyay, Anubhab Mukherjee, Akash Chaurasiya, Kumar Pranav Narayan

**Affiliations:** 1 Laboratory of Molecular Medicine, Department of Biological Sciences, Birla Institute of Technology and Science Pilani, Hyderabad Campus, Hyderabad, India; 2 Department of Pharmacy, Birla Institute of Technology and Science Pilani, Hyderabad Campus, Hyderabad, India; 3 Esperer Institute of Oncology and Metabolic Sciences (EIOMS), Mumbai, India

**Keywords:** colorectal cancer, gut dysbiosis, gut–immune axis, 16S rRNA sequencing, immunomodulation, probiotics, chemoprevention, nutraceutical formulation

## Abstract

**Introduction:**

The prevention of colorectal cancer (CRC) and its possible relapse remains a daunting challenge due to the unclear mechanisms governing gut dysbiosis and its interaction with the gut–immune axis. The present study aimed to investigate the preventive role of a polyherbal immunostimulatory formulation to restoring microbial diversity in gut and preventive management of colon cancer.

**Methods:**

We developed a novel polyherbal and probiotic-based immunostimulatory formulation with four variants (F3A, F3B, F3C, and F3D), incorporating botanical extracts and probiotics. Initially, plant-derived biological lead compounds were identified using LC/MS/MS. Next, the identified biological leads were subjected to *in-silico* molecular docking and molecular dynamics simulations with apoptotic biomarker, oncogenic marker, and immunomodulatory biomarkers (i.e., BCL2/BAX, KRAS, BRAF, IL-6, and IL-10), respectively. Followed by, the cellular cytotoxicity of the formulations were analyzed in murine colon cancer cell line (CT26), human colorectal adenocarcinoma cell line (HT29), and normal human embryonic kidney cells (HEK-293). All four formulations were tested for their immunomodulatory potential by western blot analysis in in CT26 cell line. Furthermore, *E. coli* growth inhibition, and T-cell and B-cell proliferation were examined by *ex-vivo* conditions.

**Results:**

Formulation F3B showed maximal cytotoxicity in CT26 cell line however it considered for animal experiments. Thereafter, *in-vivo* experiments were conducted using adult female BALB/c mice to investigate the modulation of gut dysbiosis. In the present study, animals were divided in three groups (normal control, diseases control, treatment control) while each group consisted of *n* = 6 animals.

**Discussion:**

Our findings demonstrate that the proposed formulations exhibit anti-proliferative, mild pro-apoptotic, immunomodulatory, and antimicrobial effect. Based on 16S rRNA sequencing results, F3B demonstrated better outcomes toward managing gut dysbiosis in female BALB/C mice. However, we can conclude that formula 3FB can be used as preventive approach to control gut dysbiosis and possible colon cancer relapse.

## Graphical Abstract

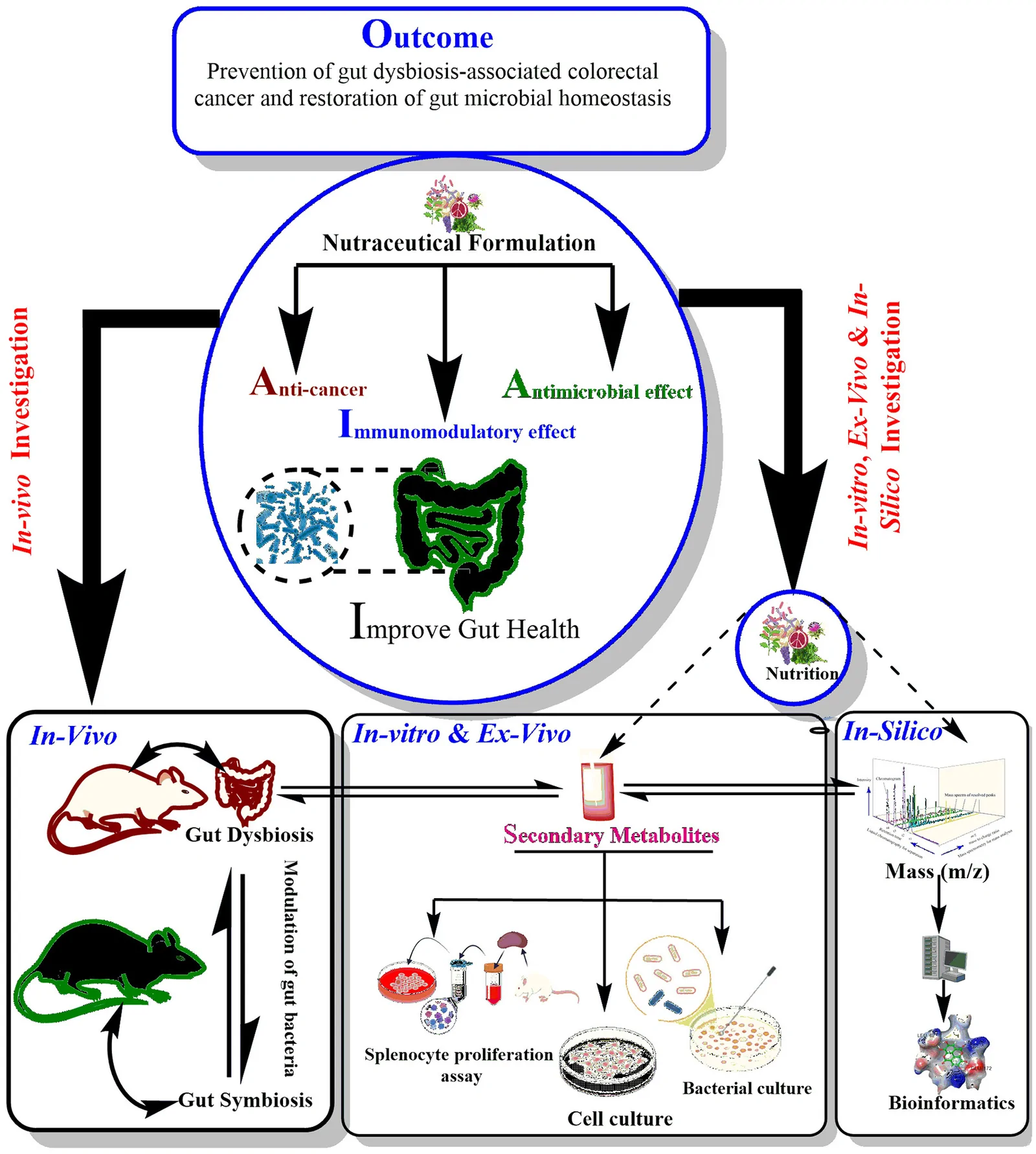

## 1 Introduction

Colorectal cancer (CRC) remains one of the most significant global malignancies, representing a formidable public health challenge, ranking as the third most diagnosed cancer and second leading cause of cancer-related mortality globally. According to the GLOBOCAN report of 2022, CRC remains ranked third, with 1,926,425 new cases and 904,019 deaths (1). CRC is a complex disease characterized by stepwise progression from normal colorectal epithelium to invasive and metastatic tumors, driven by the accumulation of genetic and epigenetic alterations (2, 3). Recently, scientists have been paying more attention to gut microbiome studies. The gut is often referred to as the “second brain” because of its extensive bidirectional communication with the central nervous system. Trillions of microbes reside in the gut microbiome. Thus, some of them are pathogenic and produce toxic metabolites, which lead to prompt inflammation and eventually enhance the chance and progression of colorectal cancer (4). When there is an imbalance in these gut microbes, called dysbiosis, the harmful bacterial population increases, and healthy bacterial diversity decreases (5). Similar to many other diseases, a combination of various genetic and environmental factors determines the genesis and progression of neoplastic growth in the colon (6). The detrimental roles of microorganisms and environmental factors have been increasingly recognized in cancer biology. Specific enteropathogenic bacteria, such as *Fusobacterium nucleatum, Bacteroides fragilis*, and certain strains of *Escherichia coli*, are known to promote chronic inflammation, modulate host immune responses, and induce DNA damage and chromosomal instability (CIN) (6). Evidence suggests that chromosomal instability (CIN) accounts for approximately 84% of sporadic CRC cases and is prevalent in familial adenomatous polyposis (FAP). This pathway is marked by chromosomal aberrations, including somatic copy number alterations (SCNAs) and mutations or deletions in key genes, such as adenomatous polyposis coli (APC), deleted in colorectal cancer (DCC), and p53 silencing (7). CIN involves the activation of proto-oncogenes, such as BRAF and KRAS, and the inactivation of tumor suppressor genes, including APC (located on 5q21-22), p53 (at 17p13), and loss of heterozygosity (LOH) at 18q, facilitating colorectal carcinogenesis (8). This dysbiotic state drives primary tumor development and contributes to disease recurrence and poor prognosis. The absence of adequate nutrition exacerbates gut dysbiosis, perpetuating inflammation conducive to inflammatory bowel disease (IBD) and increasing CRC risk (9–13). Dysbiosis also activates pro-inflammatory signaling, suppresses immune surveillance, stimulates angiogenesis, and contributes to epigenetic changes that favor malignant transformation. In the context of the tumor microenvironment, tumor-associated microbiota may influence cell infiltration and immune checkpoint expression, and thus, prognosis (14). Microbial alterations may persist after surgical resection or chemotherapy, which may cause chronic inflammation and micrometastatic niches, thereby increasing the risk of relapse. To address these complications and the intricate interplay between gut dysbiosis, inflammation, and CRC progression, this study offers an innovative probiotic and prebiotic-based immunostimulatory supplement that provides a preventive strategy to combat CRC and restore gut homeostasis. This formulation is underpinned by a strong scientific rationale that leverages the established anti-inflammatory, anti-proliferative, and pro-apoptotic properties of its individual components. The central hypothesis is that this synergistic blend (probiotics & plant extract) can modulate the gut microbiome, mitigate inflammatory cascades, and directly inhibit colorectal cancer cell proliferation and survival (15). The proposed formulations primarily differ in the proportions of the incorporated botanical ingredients to evaluate their potential synergistic effects on antioxidant, immunomodulatory, and gut health-promoting activities. Briefly, F3A is enriched with *Petroselinum crispum, Malus domestica*, and *Camellia sinensis*; F3B contains a balanced combination of all selected phytonutrients; F3C is enriched with *Silybum marianum* and *Vitis vinifera*; whereas F3D primarily consists of *Vitis vinifera, Allium sativum, Punica granatum*, and *Camellia sinensis*. The clinical significance of this project lies in its potential to offer a preventative and adjunctive therapeutic option for CRC management, particularly reversing gut dysbiosis to symbiosis, which helps manage chronic inflammation, adaptive immune responses, and restore microecological balance by reconstituting gut microbial homeostasis, increasing beneficial SCFA producers, and reducing the abundance of CRC-associated pathogens.

This study employed a comprehensive experimental design to evaluate the efficacy and safety of this novel probiotic and prebiotic-based immunostimulatory formulation. The experiments included *in-silico* (bioinformatics), *in-vitro* (cell culture and bacterial culture), and *in-vivo* studies. First, we utilized advanced tools such as FE-SEM and LC-MS/MS to analyze the components of the supplement and then employed computer modeling to predict how its proteins might interact with other molecules. To evaluate the effectiveness on cancer cells (HT-29 and CT26) and the safety of normal cells (HEK-293), cellular assays, such as MTT and Annexin V-FITC, were employed. Proteomics, specifically western blot analysis, was used to investigate apoptotic and inflammatory pathways as well as the levels of oncogenic proteins KRAS and BRAF in the murine colon carcinoma cell line (CT-26). Microbiological tests were performed on pathogenic *E. coli*. The secondary metabolites of each probiotic strain (*Lactobacillus casei, Lactobacillus rhamnosus, Lactobacillus plantarum, and Bifidobacterium lactis*) were incorporated into the formulation, which effectively inhibited the growth of *E. coli*. Immunomodulation was evaluated based on splenocyte proliferation, and immunoglobulin levels were assessed. *In-vivo* experiments were conducted on a BALB/c mouse model of gut dysbiosis to confirm the restoration of microbial balance (via 16S rRNA gene sequencing) and evaluate the physiological, hematological, and histopathological effects. Our metagenomic analysis identified *Bacteroides fragilis, Escherichia coli, Enterococcus faecalis*, and *Streptococcus gallolyticus* as being mechanistically linked to CRC. This comprehensive approach aims to validate the formulation as a preventive measure to restore gut dysbiosis to a symbiotic state, thereby aiding in colon cancer management and reducing the risk of recurrence (16, 17). Additionally, it significantly contributes to the management of colon cancer, converting gut dysbiosis to symbiosis and activating the immune system, ultimately lowering the risk of colorectal cancer relapse associated with gut dysbiosis.

## 2 Material and method

All chemicals and reagents were acquired from reputable vendors. Formulation ingredients were supplied by Esperer Onco Nutrition (India). Cell culture-grade plasticware and consumables were procured from HiMedia, Gibco, Cell Signaling Technology, Sigma, and Thermo Scientific. Specific consumables were arranged in the following order: antibiotics (penicillin and streptomycin), LB Agar, MRS broth, thioglycolate broth, MTT reagent (Cat No: #TC-191), and RBC lysis buffer (Cat No: #R075) were purchased from HiMedia. DMEM and FBS were obtained from Grand Island, NY, USA. Antibodies against Bax B-RAF (Cat No: #14814), K-RAS (Cat No: #71835) (Cat No: #2772), Bcl-2 (Cat No: #3498), and b-actin (Cat No: #4970) were purchased from Cell Signaling Technology (Danvers, MA, USA). RIPA buffer (Cat No: #R0278), Concanavalin A (ConA), and lipopolysaccharide (LPS) were obtained from Sigma–Aldrich (St. Louis, MO, USA). The Annexin V-FITC apoptosis kit (Cat No: V13242), ECL substrate for blot development (Cat No: #34075), protease inhibitor cocktail (Cat No: #78429), and BCA kit (Cat No: #23225) were purchased from Thermo Fisher Scientific (Waltham, MA, USA). Animal-related consumables including ketamine, xylazine, and isoflurane were procured from a licensed medical shop. BALB/c mice were acquired from a registered vendor approved by CPCSEA, Jeeva Life Sciences Private Limited (Reg. No 1757IPO/RCBiBINRc/S/14/CPCSEA).

### 2.1 Formulation development

Powder blending was used to design the final formulation. Appropriate amounts of ingredients were weighed according to the formula: (parsley extract 250 mg, apple extract 100 mg, *Silybum marianum* extract 175 mg, grape seed extract 75 mg, *Allium sativum* extract 125 mg, green tea extract 125 mg, pomegranate extract 60 mg, *Lactobacillus plantarum 162 mg, Lactobacillus rhamnosus 162 mg, Lactobacillus casei 162 mg, Bifidobacterium lactis 162 mg*, thaumatin 150 mg, and ascorbyl palmitate 300 mg). All the ingredients were mixed using a mortar and pestle. After complete mixing, the mixture was blended using a grinder (5–10 min). Thereafter, the blended mixture was sieved using a sieve of size 60 μm to yield an amorphous powder. Furthermore, the formulations were packed into sachets with precise sealing and labeling. The same procedure was followed for formulations (3A–D) except for the amounts.

### 2.2 *In-vitro* & *in-silico* characterization

#### 2.2.1 Powder morphology by FE-SEM

Powder morphology analysis was performed using field-emission scanning electron microscopy **(**FE-SEM). The procedures are reported in the Supplementary material.

#### 2.2.2 Lead compound identification by liquid chromatography-mass spectrometry (LCMS)

The biologically active compound was identified from the plant extract using Shimadzu Liquid Chromatography Mass Spectrometry **(**LC-MS/MS) (model no. SIL-20AC, detector LCMS-8040). The detailed procedure is reported in the Supplementary material (18, 19).

#### 2.2.3 Bioinformatics (protein and ligand interaction)

Next, the biologically active lead compounds identified above were docked with various target proteins, including BAX, BCL-2, K-RAS, B-RAF, IL6, and IL10. Docking and molecular dynamics (MD) simulations were performed using the licensed version of Schrödinger software (Maestro 2025-2) (20). The MD simulations were conducted for a duration of 100 ns. The selected proteins were docked with the biological lead compounds quercetin, apigenin, silymarin, anthocyanidins, allicin, EGCG, and ellagic acid present in our probiotic and prebiotic-based immunostimulatory formulation (21). The appropriate crystal structures of these proteins were retrieved from the Protein Data Bank (PDB) database. Docking was performed using the following structures: BCL2 (PDB ID: 2O2F), IL6 (PDB ID: 1ALU), IL10 (PDB ID: 2ILK), B-RAF (PDB ID: 1UWH), K-RAS (PDB ID: 8AZX), and BAX (PDB ID: 6EB6). The binding sites for each target were defined based on co-crystallized ligands or key active-site residues. In the present study, based on the reported therapeutic potential, quercetin was chosen for the molecular dynamics study. The MD simulations were conducted for a duration of 100 ns, and the program was run on a supercomputer (22). Additionally, we performed the bioavailability prediction by swissdock online tools under the CC 4.0 license of SIB Swiss Institute of Bioinformatics.

### 2.3 Cell & bacterial culture and maintenance

The murine colon carcinoma cell line (CT 26), human colorectal adenocarcinoma cell line (HT-29), and normal human embryonic kidney cell line (HEK-293) obtained from authenticated sources and maintained at the cell culture facility of BITS-Pilani Hyderabad Campus. Cells were cultured Dulbecco's modified Eagle's medium (DMEM) supplemented with 10% Fetal Bovine Serum (FBS) and 1% penicillin-streptomycin in a humidified atmosphere containing 5% CO_2_ at 37 °C with 5% CO_2_. Cells were routinely passaged at 70%−80% confluency and experiments were performed using cells between passages 5 and 20. All cell lines were periodically tested and confirmed to be free of mycoplasma contamination prior to experimental use.

Bacterial cultures were performed under biosafety levels 1 or 2 conditions as appropriate. For antimicrobial and dysbiosis-related assays, bacterial strains were cultured in Luria-Bertani (LB) broth for aerobic growth and De Man, Rogosa, and Sharpe (MRS) broth for culturing of probiotic cultures. Thioglycolate medium was used to culture facultative anaerobic or anaerobic bacteria. Anaerobic cultures were maintained in sealed containers with oxygen-limited conditions. Bacterial growth was monitored by measuring optical density at 595 nm.

#### 2.3.1 Cell cytotoxicity assay

The colon cancer cell line (CT 26) were seeded at a density of 7,000 cells/well in a 96-well plate and incubated overnight. The cells were then treated with different concentrations of formulations, such as F3A, F3B, F3C, and F3D. Each formulation was dissolved in sterile 1 × PBS buffer, diluted in DMEM, and filtered through a 0.22 μm syringe filter prior to treatment. Subsequently, CT26, HT29, and normal human embryonic kidney (HEK293) cells were treated with different concentrations (0.25, 0.125, 0.063, and 0.0312 %/w/v) for 24, 48, and 72 h, respectively. Following treatment, MTT solution (0.5 mg/mL) was added to each well at the indicated time points and incubated for 4 h (22). The medium was then carefully removed, and 50 μL of culture-grade DMSO was added to each well to dissolve the formazan crystals. Absorbance was measured at 570 nm using a SpectraMax microplate reader (Model ID3). The same procedure was followed to assess the cytotoxicity of probiotic-derived secondary metabolites. Cell viability was calculated using the GraphPad Prism software.

#### 2.3.2 Apoptosis assay by flow cytometry

Murine colon carcinoma cells (CT26) were seeded at a density of 2.5 × 10^4^ cells per well in six-well plates and incubated for 24 h to facilitate cellular adherence. Following initial attachment, the cells were exposed to 0.1% (w/v) concentrations of the test formulations (F3A-F3D) for a subsequent 24-h period. After treatment, the cells were thoroughly rinsed with 1 × PBS and detached via trypsinization. Cell pellets obtained by centrifugation were resuspended in 200 μL of binding buffer containing annexin-V conjugated to fluorescein isothiocyanate (FITC) at a final concentration of 0.25 μg/mL. The suspension was gently vortexed and incubated in the dark for 20 min to allow optimal staining. Thereafter, propidium iodide (PI) was added to a final concentration of 1.0 μg/mL to enable the discrimination of late apoptotic and necrotic cells. Stained samples were analyzed by flow cytometry on a BD FACS Canto II system, and data for cellular populations were acquired and interpreted across four standard quadrants (Q1–Q4), allowing for the quantitative assessment of apoptosis and cell viability.

#### 2.3.3 Proteomics study (western blot analysis)

CT26 cells were seeded at a density of 2.5 × 10^5^ cells/well and incubated for 18–24 h. The cells were then treated for 24 h with formulations F3A-F3D at their MTT-derived IC_50_ concentration (0.1% w/v), as well as untreated controls. The following day, the cells were harvested by gentle scraping and proteins were extracted using RIPA buffer (Sigma) on ice. Lysates were centrifuged at 13,000 rpm for 20 min at 4 °C, and the resulting supernatants were collected into 1.5 mL microcentrifuge tubes for protein quantification using the Bradford assay. Equal amounts of protein (20 μg per sample) and 3 μL of protein ladder were loaded onto a 12% SDS-PAGE gel. Electrophoresis was performed at 80 V for stacking and at 120 V for resolution. Proteins were then transferred onto a PVDF membrane (Millipore) using the wet transfer method. Membranes were blocked with 3% BSA in PBS-T for 2 h at room temperature, followed by incubation with primary antibodies (1:1000 dilution), including rabbit anti-Bcl-2, anti-Bax, anti-IL6, anti-IL10, and anti-ß-actin (used as the loading control), overnight at 4 °C with gentle shaking. After washing, the membranes were incubated for 2 h with 1:10000 diluted biotinylated goat anti-rabbit secondary antibody. Protein bands were visualized using enhanced chemiluminescence (ECL) solution (Thermo Fisher Scientific) and detected using chemiluminescent imaging. The experiments were independently repeated thrice (*n* = 3) to confirm reproducibility.

#### 2.3.4 *E. coli* growth inhibition assay

An *E. coli* growth inhibition assay was performed using a spectrophotometric method (23). The effects of probiotic-derived secondary metabolites (PDSM) and phytoconstituents were evaluated against *Escherichia coli (E. coli)*. Briefly, an overnight culture of *E. coli* was grown in a nutrient broth at 37 °C with shaking at 150 rpm. The culture was then diluted in fresh medium to an initial OD density of ~0.1. The bacterial suspension was subsequently treated with different combinations of probiotic-derived secondary metabolites and crude plant-derived phytoconstituents both individually and in combination. Treatment groups included secondary metabolite (SM) alone, formulation (F3B) alone, and SM: F3B mixtures at different ratios of 1:2, 1:1, and 2:1. The untreated bacterial culture served as a control. All samples were incubated at 37 °C under identical conditions, and bacterial growth was monitored spectrophotometrically by measuring optical density at 600 nm (OD600) at 2-h intervals throughout the incubation period.

#### 2.3.5 *E. coli* viability by CFU plate count

Aliquots of the same cultures were collected at the 4th and 8th hours for viability assessment by colony-forming unit (CFU) counting. Serial dilutions of the treated cultures were prepared in sterile saline, and 100 μL aliquots from appropriate dilutions were spread in triplicate onto nutrient agar plates. The plates were incubated overnight at 37 °C, after which bacterial colonies were enumerated to determine the CFU per milliliter (CFU/mL) using standard microbiological procedures.

### 2.4 *In-vivo* animal experiments

Female adult BALB/c mice, weight 15 to 20 g were procured from a CPCSEA-registered vendor such as M/S Jeeva Life Sciences, Hyderabad, India (Registration No. 1757/PO/RcBiBt/S/14/CPCSEA) following approval from the Institutional Animal Ethics Committee (IAEC), BITS Pilani, Hyderabad Campus (Approval No. BITS/HYD/IAEC/2022-20). Animals were housed in polypropylene cages under standard laboratory conditions (22 ± 2 °C, 50%−60% relative humidity, and a 12 h light/12 h dark cycle) with *ad libitum* access to a standard pellet diet and drinking water. All animals were acclimatized for 1 week prior to the initiation of the study. During experimental procedures required anesthesia such as isoflurane were used by mixing of 5% isoflurane in 95% oxygen for induction and 1.5%−2.5% for maintenance via inhalational method. At the end of the experiments, the animals were scarified by excessive anesthesia followed by cervical dislocation as per IAEC protocol.

#### 2.4.1 Gut dysbiosis model development in BALB/C mice

A gut dysbiosis model was developed in adult female BALB/c mice at 10–12 weeks of age. The detailed induction protocols are reported in the Supplementary material. Adult female BALB/c mice were used in this study due to their well-characterized immune phenotype and widespread application in immunological and gut microbiota-immune interaction studies. Female mice were selected to minimize variability associated with sex-dependent behavioral and hormonal differences, thereby ensuring experimental consistency.

#### 2.4.2 Validation of gut dysbiosis on BALB/c mice model

In this investigation, a range of physiological parameters was rigorously monitored to evaluate the impact of the intervention. The body weights of the animals were measured on days 0 and 21, and the percentage change was calculated to quantify alterations in growth or overall health status. Gastrointestinal function was assessed by analyzing the length and frequency of fecal pellets. Fecal pellets were collected randomly from the sanitized cages, and their lengths were measured. On day 28, each mouse was individually housed in a clean cage for 1 h to record the number of pellets excreted, thereby providing metrics of intestinal motility and stool consistency. Additionally, a gastrointestinal transit assay was performed at the end of the treatment period. Following an overnight fast, mice were administered 5% Evans blue in 1.5% methylcellulose via oral gavage, and the interval to the appearance of the first blue-stained fecal pellet was recorded at 10-min intervals. Fecal water content was determined by weighing freshly collected fecal pellets before and after drying at 60 °C overnight, serving as an index of hydration status.

#### 2.4.3 Modulation of gut dysbiosis to symbiotic by spectroscopic and FE-SEM

A total of female 36 adult BALB/c mice, each weighing 25–30 g, were enrolled in the study. The animals were randomized into six groups: four probiotic treatment cohorts, one dysbiotic control, and one normal control group. Dysbiosis was induced in the relevant groups by administering a combination of ciprofloxacin and metronidazole orally for 14 consecutive days. Prolonged antibiotic exposure was used to induce sustained microbial imbalance, which is known to elicit systemic immune alterations beyond the gut. Model establishment was validated by culturing fecal samples in thioglycolate medium for 48 h, followed by quantification of the bacterial load using optical density (OD) measurements at 600 nm. Validation assessments were conducted on days seven and 24 of the antibiotic regimen. Upon completion of dysbiosis induction, antibiotics were withdrawn, and probiotic-based formulations (designated F3A-F3D) were administered daily by oral gavage at a dose of 250 mg/kg body weight for 14 days. All groups received treatments at 24-h intervals. At the end of the experimental period, fecal samples from each group were randomly collected and incubated in thioglycolate medium for 48 h to evaluate the post-treatment microbial composition. Bacterial load was determined by measuring the OD at 600 nm, and the results were plotted to compare treatment groups with controls. Bacterial diversity was further assessed using field-emission scanning electron microscopy (FE-SEM). Cultured bacteria were isolated in 1 × PBS and fixed with 2.5% glutaraldehyde in saline for 30–60 min. Aliquots of 20 μL were placed onto 18 mm round glass coverslips, dried overnight under vacuum, and imaged at magnifications of 5 μm, 2 μm, and 1 μm.

#### 2.4.4 Hemolysis test

Blood samples were collected from BALB/c mice via the retro-orbital plexus. The collected blood was washed three times with sterile 0.9% saline by centrifugation at 180 × g for 5 min to separate the red blood cells (RBCs). A 0.5 mL aliquot of a 0.5% RBC suspension was prepared for the assay. The RBC suspension was then mixed with an equal volume of the test compounds (formulation 3B) at concentrations of 0.25%, 0.125%, 0.063%, and 0.0312% w/v. The mixture was incubated at room temperature for 1 h to assess hemolysis. The extent of hemolysis was quantified by measuring the absorbance of free hemoglobin in the supernatant at 540 nm. The percentage of hemolysis was calculated according to Equation 1.

$$\begin{array}{l} Hemolysis\;\left(\%\right)=\frac{OD_{Hem}^{Sample}-OD_{Hem}^{Negt.}}{OD_{Hem}^{Total}-OD_{Hem}^{Neg}}\times 100. \end{array}$$ (1)

#### 2.4.5 Hematological test (WBC count, RBC count, hemoglobin, neutrophils, lymphocytes)

Complete Blood Count (CBC) analyses were conducted on three groups of mice: normal control, dysbiotic control, and treated group, which received the most efficacious formulation, 3 B. Blood was collected from all animals via retro-orbital plexus sampling and processed at the pathology laboratory. Hematological parameters, including white blood cells (WBC), red blood cells (RBC), hemoglobin (HB), neutrophils, and lymphocytes, were quantified using the electrical impedance method with a Horiba cell counter. The resulting data were subsequently compiled and used to generate comparative graphical representations across all the experimental groups.

#### 2.4.6 Reverse the gut dysbiosis by confirming 16S rRNA sequencing

Genomic DNA was isolated from fecal samples obtained from three experimental groups: normal, dysbiotic, and treated controls. Approximately 25 mg of fecal material per sample was processed using the Xploregen DNA extraction kit, according to the manufacturer's protocol. Following extraction, 16S rRNA gene regions were amplified by Polymerase Chain Reaction (PCR). Amplicons were subsequently purified using Ampure beads to eliminate residual primers. To facilitate downstream sequencing, eight additional cycles of PCR were performed using Illumina barcoded adapters, thereby generating sequencing-ready libraries. Libraries were further purified with Ampure beads and quantified using a Qubit dsDNA high sensitivity assay kit. Sequencing was conducted on an Illumina MiSeq using a 2 × 300 bp paired-end v3-v4 sequencing kit. The sequencing data were subsequently analyzed, and heatmaps and venn diagrams were generated to evaluate the changes in bacterial diversity and assess the reversal of dysbiosis (24).

#### 2.4.7 *Ex-vivo* immunomodulation assay for T-cell & B-cell

To determine whether F3B and SMs caused any toxicity to normal unstimulated mouse splenocytes, cell viability was assessed by MTT assay after a 24-h exposure to various concentrations of F3B and SMs of the four probiotic strains. Spleen was isolated from healthy mice and aseptically processed to obtain a single-cell suspension via mechanical disaggregation and filtration through a 40 μm nylon mesh. Erythrocytes were lysed in 1 × RBC lysis buffer for 2 min. The remaining splenocytes were washed, resuspended in complete RPMI-1640 medium supplemented with penicillin, streptomycin, kanamycin, and 10% FBS, and then counted. Cells were seeded into 96-well plates at a density of 5 × 10^5^ cells/well. After 2 h, the cells were treated with free Concanavalin A (ConA, 5 μg/mL), free lipopolysaccharide (LPS, 10 μg/mL), and a combination of plant formulations (0.2% w/v) and probiotic-derived secondary metabolites (0.2% w/v). Both F3B and SM were mixed in equal ratios with four different probiotic strains: *Lactobacillus casei, Lactobacillus rhamnosus, Lactobacillus plantarum, and Bifidobacterium lactis*. The F3B-SM cocktail was combined with ConA and LPS in equal ratios and incubated for 48 h. Cell proliferation was assessed using the MTT assay as follows: 20 μL of 5 mg/mL MTT solution was added and incubated for 4 h, followed by the addition of 50 μL DMSO after centrifugation. Absorbance was measured at 570 nm and the percentage of T and B cell proliferation was calculated.

#### 2.4.8 Immunomodulation assay (IgA, IgM, and IgG)

In these experiments, only the most effective formulation, 3B, was used. Mice in the treatment group received 250 mg/kg body weight daily for 14 days, beginning on the day the dysbiosis model was established. On day 28, blood samples were collected from all the mice via the retro-orbital plexus and sent to the pathology laboratory for analysis. The samples were thawed and equilibrated before further dilution with specific anti-mouse IgA, IgM, and IgG reagents using nephelometer cuvettes. Readings were recorded, and the resulting data were used to generate comparative graphs.

#### 2.4.9 Histopathology

The experiment concluded on day 28, at which point major organs, including the liver, lungs, kidneys, heart, spleen, and colon, were collected from all experimental groups of mice. Organs were immediately fixed with 10% formalin to preserve the tissue architecture and subsequently processed for paraffin embedding. The embedded tissues were sectioned at 5–10 μm thickness using a microtome and carefully mounted onto glass slides. For histological analysis, the sections were deparaffinized with xylene and rehydrated using a graded ethanol series. The rehydrated sections were stained with hematoxylin to label the cell nuclei blue-purple, rinsed, and counterstained with eosin to visualize the cytoplasm and extracellular matrix in pink. Finally, the slides were dehydrated, cleared with xylene, and cover-slipped for microscopic examination. Representative images were captured at 4 ×, 10 ×, and 20 × magnifications.

## 3 Results

### 3.1 Formulation development

As described in the preceding methods section, the proposed formulation comprises major components primarily derived from plant sources (plant extract), including apigenin from *Petroselinum crisped* (Parsley extract), Quercetin from *Malus domestica* (Apple extract), Silymarin from *Silybum marianum extract*, Anthocyanidins from *Vitis vinifera* (Grape seed extract), *Allicin* from *Allium sativum* (Garlic extract), EGCG from *Camellia sinensis* (Green tea extract), and ellagic acid from *Punica granatum* extract probiotics (probiotics, *Lactobacillus plantarum, Lactobacillus rhamnosus, Lactobacillus casei, Bifidobacterium lactis*), natural sweeteners (thaumatin), and preservatives (Ascorbyl Palmitate). All the phytoconstituent composition, as described in the formulation Table 1, with F3B demonstrated better cytotoxicity however, further same formulation used in *in-vivo* gut dysbiosis study. Table 2 mentions the concentration of secondary metabolite derived from each probiotic strain. The selection of each phyto extract in this formula was grounded in its established therapeutic effectiveness and nutrient value. Comprehensive details are provided in Supplementary Tables S1a, b.

**Table 1:** Formulation containing phytonutrients, healthy probiotics, natural sweeting agent and natural preservative and/or antioxidant, respectively.

Ingredients	F3A	F3B	F3C	F3D
*Petroselinum crisped*	325 mg	250 mg	0 mg	0 mg
*Malus domestica*	325 mg	100 mg	0 mg	0 mg
*Silybum Marianum*	0 mg	175 mg	275 mg	0 mg
*Vitis vinifera*	0 mg	75 mg	300 mg	275 mg
*Allium Sativum*	0 mg	125 mg	120 mg	300 mg
*Camellia sinensis*	250 mg	125 mg	110 mg	120 mg
*Punica granatum*	0 mg	60 mg	0 mg	110 mg
*Lactobacillus plantarum*	2.8 × 106 cfu	2.8 × 106 cfu	2.8 × 106 cfu	2.8 × 106 cfu
*Lactobacillus rhamnosus*	2.7 × 106 cfu	2.7 × 106 cfu	2.7 × 106 cfu	2.7 × 106 cfu
*Lactobacillus casei*	2.9 × 106 cfu	2.9 × 106 cfu	2.9 × 106 cfu	2.79 × 106 cfu
*Bifidobacterium lactis*	2.8 × 106 cfu	2.98 × 106 cfu	2.8 × 106 cfu	2.8 × 106 cfu
Thaumatin	150 mg	150 mg	150 mg	150 mg
Ascorbyl palmitate	400 mg	300 mg	400 mg	400 mg

**Table 2:** Summarizes the probiotic strains with secondary metabolite concentrations.

Sr. no	Probiotic organism	CFU/gm	Concentration of metabolite produced
1.	*Lactobacillus plantarum*	17.5 × 10^6^	26.6 mg/mL
2.	*Lactobacillus rhamnosus*	16.8 × 10^6^	43.3 mg/mL
3.	*Lactobacillus casei*	18 × 10^6^	30 mg/mL
4.	*Bifidobacterium lactis*	17.8 × 10^6^	26.6 mg/mL

### 3.2 Powder morphology determined by its FE-SEM

**Figure 1:**
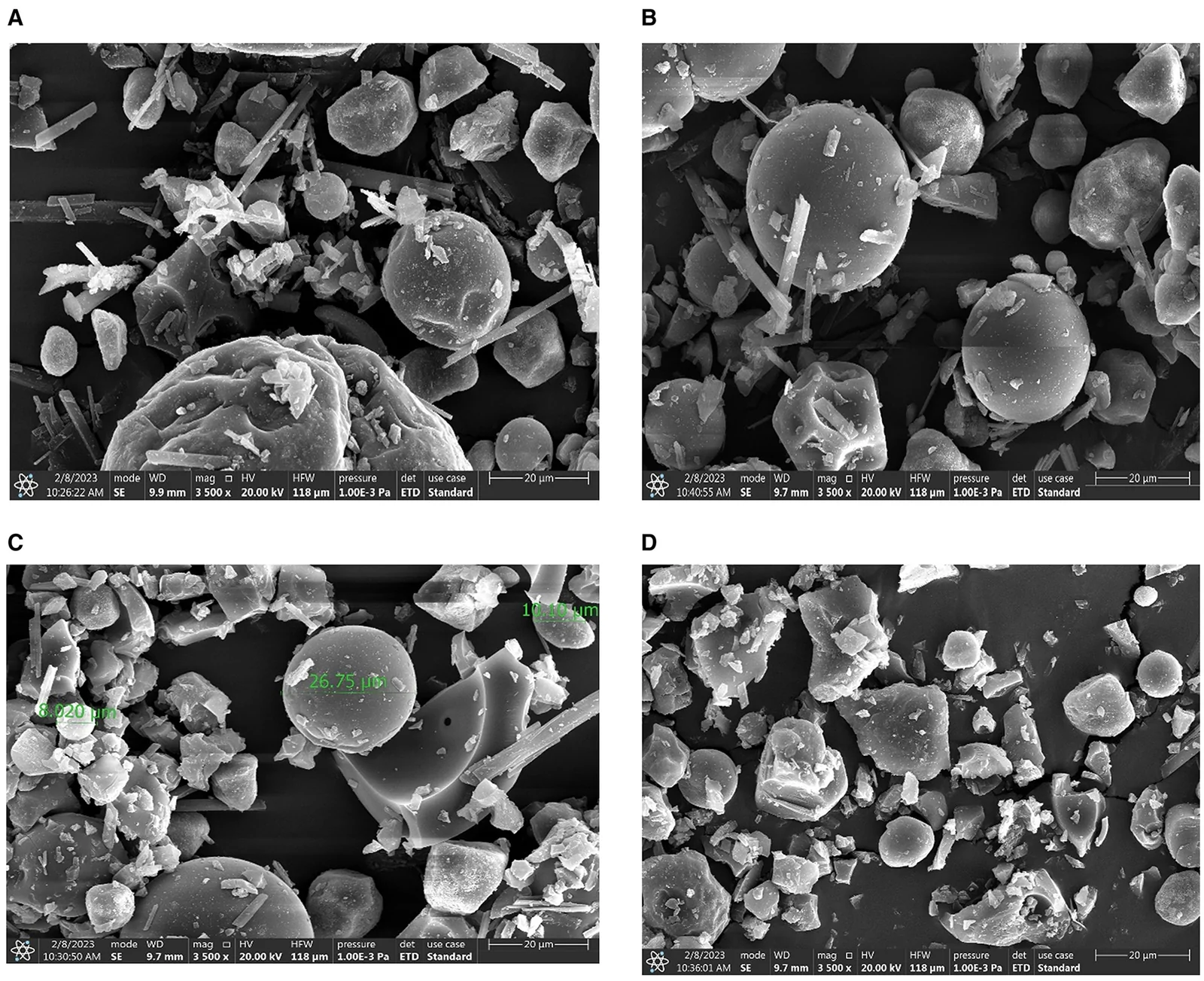
Size and morphology of powder formulations containing PNs. **(A)** F3A; **(B)** F3B; **(C)** F3C; and **(D)** F3D. All formulations exhibited predominantly spherical or near-spherical aggregate microparticles with a mean particle size within the 20 μm range, indicating uniform morphology and consistent particle distribution across samples. No significant morphological differences were observed among the formulations.

Field-emission scanning electron microscopy (FE-SEM) was used to elucidate the morphological characteristics of the powdered formulations. The present in formulations F3A, F3B, F3C, and F3D were analyzed for size and morphology using FE-SEM at high resolution with 20 μm and 50 μm scales. The aggregate microparticles showed mostly spherical morphology within a 20 μm range. Representative images are shown in Figure 1.

### 3.3 Identification of lead compounds using LC-MS/MS

To identify the active phytoconstituents present in the plant extracts incorporated into the formulation, we used LC-MS/MS, which is a combination of HPLC and mass spectrometry. The formulation variant F3B was used as the representative sample. The entire spectrum was collected and analyzed to identify the active compounds. As exhibited by the results, we identified apigenin in parsley extract at m/z 271, *quercetin* in apple extract at m/z 302.85, *silymarin* in *Silybum marianum* extract at m/z 484.05, *anthocyanidins* in grape seed extract at m/z 287.05, allicin in *Allium sativum* extract at m/z 164.95, epigallocatechin gallate (*EGCG) i*n green tea extract at m/z 443.4 and *ellagic acid* in pomegranate extract at m/z 204.85. The outcome was in concordance with the exact reference mass and previously observed fragmentation pattern (as indicated by ChemDraw software, version 15.1). The entire dataset, along with the structure of the active compound and representative mass spectra, are presented in Table 3. Representative mass spectra are presented in Supplementary Figures S1A–G.

**Table 3:** Biological lead compounds were identified using the LC/MS/MS method (m/z rang 90 to 800).

Biological lead compounds					Protein ligand binding score					
Lead compounds/ Structure		Reference exact Mass (g/ mol)	Fragmentation	Observed fragment (m/z)	BCL2: PDB– 2O2F	BAX: PDB–6EB6	IL6: PDB-−1ALU	IL10:PDB–2ILK	B-RAF: PDB–1UWH	K-RAS: PDB–8AZX
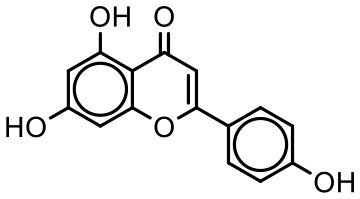	Apigenin	270.05	270.05, 271.06, and 272.06	**271**, and 269.1	−5.204	−4.540	−6.577	−4.182	−8.588	−5.409
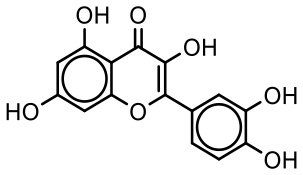	Quercetin	302.04	302.04, 303.05, and 304.05	302.85 and 301.05	−5.640	−4.131	−6.487	−5.915	−9.056	−7.142
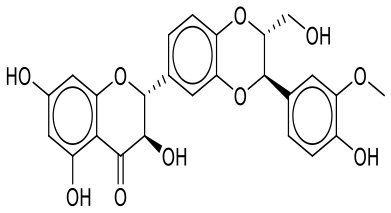	Silymarin	482.12	482.12, 483.12, and 484.13	484.05 and 486.8	−4.681	−4.540	−4.889	−4.282	−9.132	−5.814
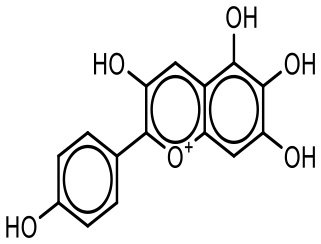	Anthocyanidins	287.06	287.06, 288.06 and 289.06	287.05, and 288.55	−5.817	−5.844	−5.343	−4.282	−8.866	−5.133
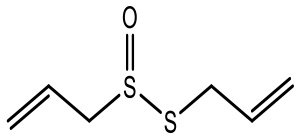	Allicin	162.06	162.06, 164.04 and 163.02	164.95 and 165.65	−3.297	−1.291	−1.720	−2.082	−3.780	−3.850
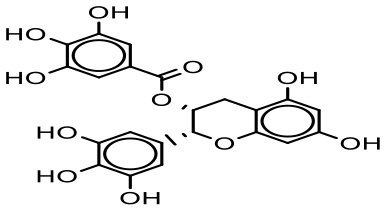	EGCG	458.08	443.10, 459.09 and 460.09	443.4, 463.15	−4.403	−3.877	−3.993	−2.156	−7.589	−2.418
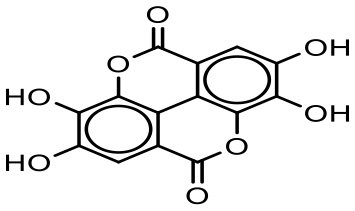	Ellagic acid	302.01	204.0, 302.01, and 303.01	204.85, and 300.1	−4.403	−4.114	−3.993	−2.983	−9.025	−3.674

These compounds include Apigenin from parsley extract, Quercetin from apple extract, Silymarin from Silybum marianum extract, Anthocyanidins from grape seed extract, Allicin from Allium sativum extract, EGCG from green tea extract, and Ellagic acid from pomegranate extract. The plant origins of these compounds were confirmed, and their representative mass spectra are provided in the Supplementary material. Subsequently, the identified phyto-compounds were subjected to protein–protein interaction analysis using molecular docking. This analysis involved various proteins, specifically BCL2 and BAX (apoptotic biomarkers), IL6 and IL10 (proinflammatory cytokines), and BRAF and KRAS (oncogenic protein biomarkers). Comprehensive details regarding the docking results and their discussion are summarized in the Supplementary material. The bold value (Quercetin) showed high docking score with all proteins target.

### 3.4 Bioinformatics (molecular interaction analysis of quercetin with target proteins)

**Figure 2:**
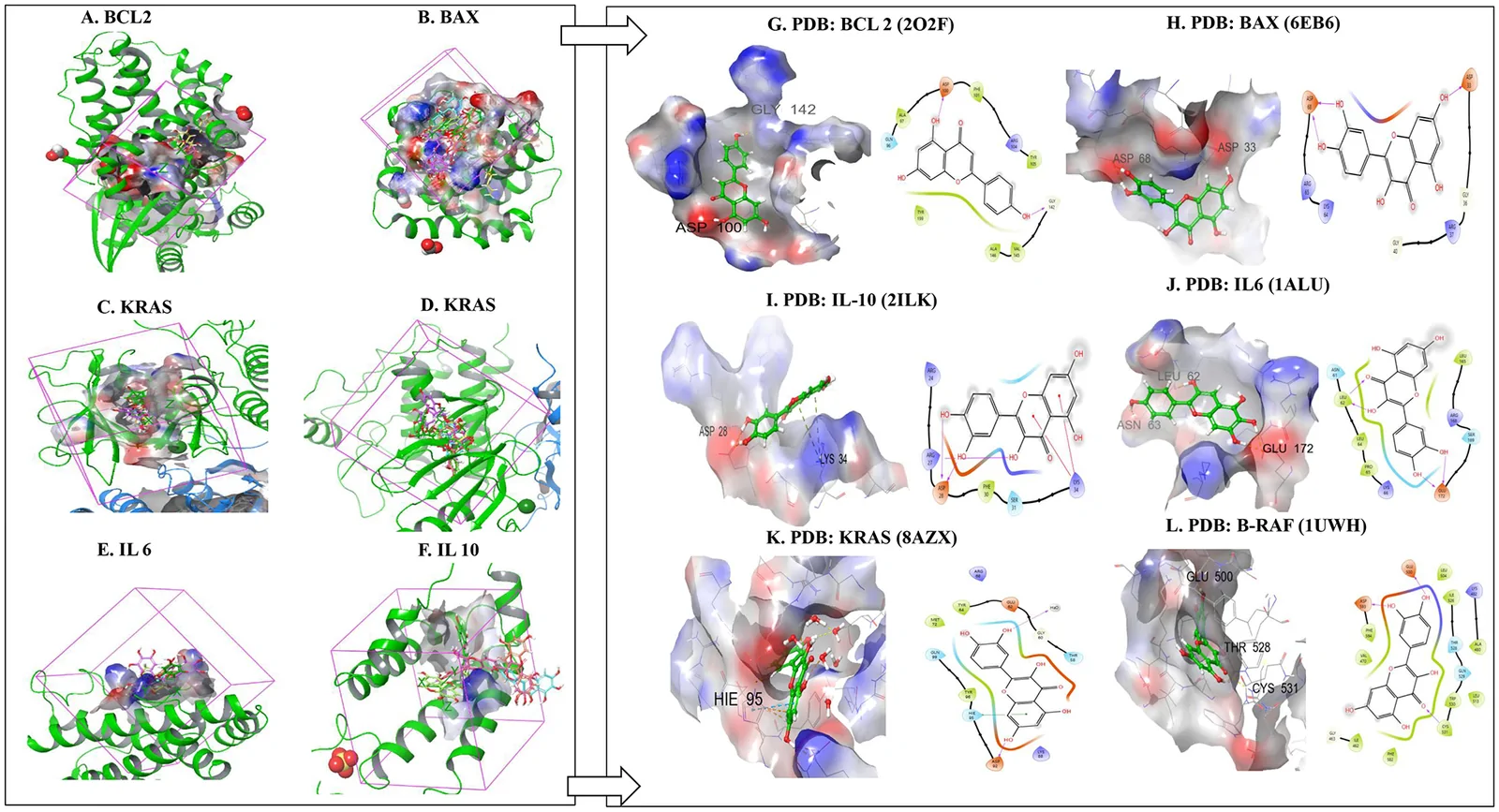
Molecular docking analysis of the lead phytochemical (Quercetin) target proteins. Upper panel **(A–F)**: Three-dimensional (3D) molecular docking conformations illustrating the binding orientation of the ligand within the active-site binding pocket of **(A)** BCL-2 (PDB ID: 2O2F), **(B)** BAX (PDB ID: 6EB6), **(C)** KRAS (PDB ID: 8AZX), **(D)** BRAF (PDB ID: 1UWH), **(E)** IL-6 (PDB ID: 1ALU), and **(F)** IL-10 (PDB ID: 2ILK). Protein structures are displayed in cartoon representation (green), while the ligand is shown in stick representation positioned within the predicted binding cavity. The molecular surface depicts the physicochemical properties of the binding pocket. Lower panel **(G–L)**: Enlarged views of the corresponding protein-ligand binding pockets together with two-dimensional (2D) interaction diagrams for BCL-2, BAX, KRAS, BRAF, IL-6, and IL-10. The interaction maps illustrate the major intermolecular contacts, including conventional hydrogen bonds, hydrophobic interactions, π-π stacking, π-alkyl interactions, van der Waals contacts, and electrostatic interactions, which collectively contribute to the stability and binding affinity of the ligand within the respective protein active sites.

Protein and molecular docking analyses revealed that quercetin exhibits high-affinity and specific binding to multiple cancer-associated targets. This interaction is facilitated by an intricate network of hydrogen bonding, hydrophobic, and electrostatic forces, underscoring the multitarget potential and mechanistic versatility of quercetin in modulating the key regulators of apoptosis, inflammation, and proliferation. Initially, all targeted proteins were mapped, and their binding pocket sites were identified, with representative visual protein structures showing active site pockets and docking sites (Figures 2A–F). Subsequent analyses yielded excellent docking scores for several biological lead compounds including quercetin, apigenin, silymarin, anthocyanidins, allicin, EGCG, and ellagic acid. Specifically, quercetin exhibited notable docking scores with BCL2 (PDB: 2O2F, −5.640), IL6 (PDB: 1ALU, −6.487), IL10 (PDB: 2ILK, −5.915), B-RAF (PDB: 1UWH, −9.056), K-RAS (PDB: 8AZX, −7.142), and BAX (PDB: 6EB6, −4.131). The remaining lead compounds also demonstrated promising docking scores, and the detailed results are summarized in Table 3. Further discussion is available in the Supplementary material. All compounds were docked with target proteins, such as BAX, BCL2, K-RAF, B-RAF, IL6, and IL10, and their 3D and 2D protein structures are illustrated in Supplementary Figures S2–S7. Additionally, among the identified phytoconstituents compounds such as allicin, apigenin, and quercetin exhibited the most favorable pharmacokinetic profiles, characterized by high predicted GI absorption and a bioavailability score of 0.55, suggesting good potential for oral administration. In contrast, silymarin, epigallocatechin gallate (EGCG), and ellagic acid were demonstrated comparatively poor oral absorption, primarily due to higher molecular polarity, greater hydrogen-bonding capacity. Notably, EGCG showed the lowest predicted bioavailability score 0.17, indicating substantial limitations in systemic exposure following oral administration (Supplementary Table S2).

### 3.5 Molecular dynamics (MD) simulation

**Figure 3:**
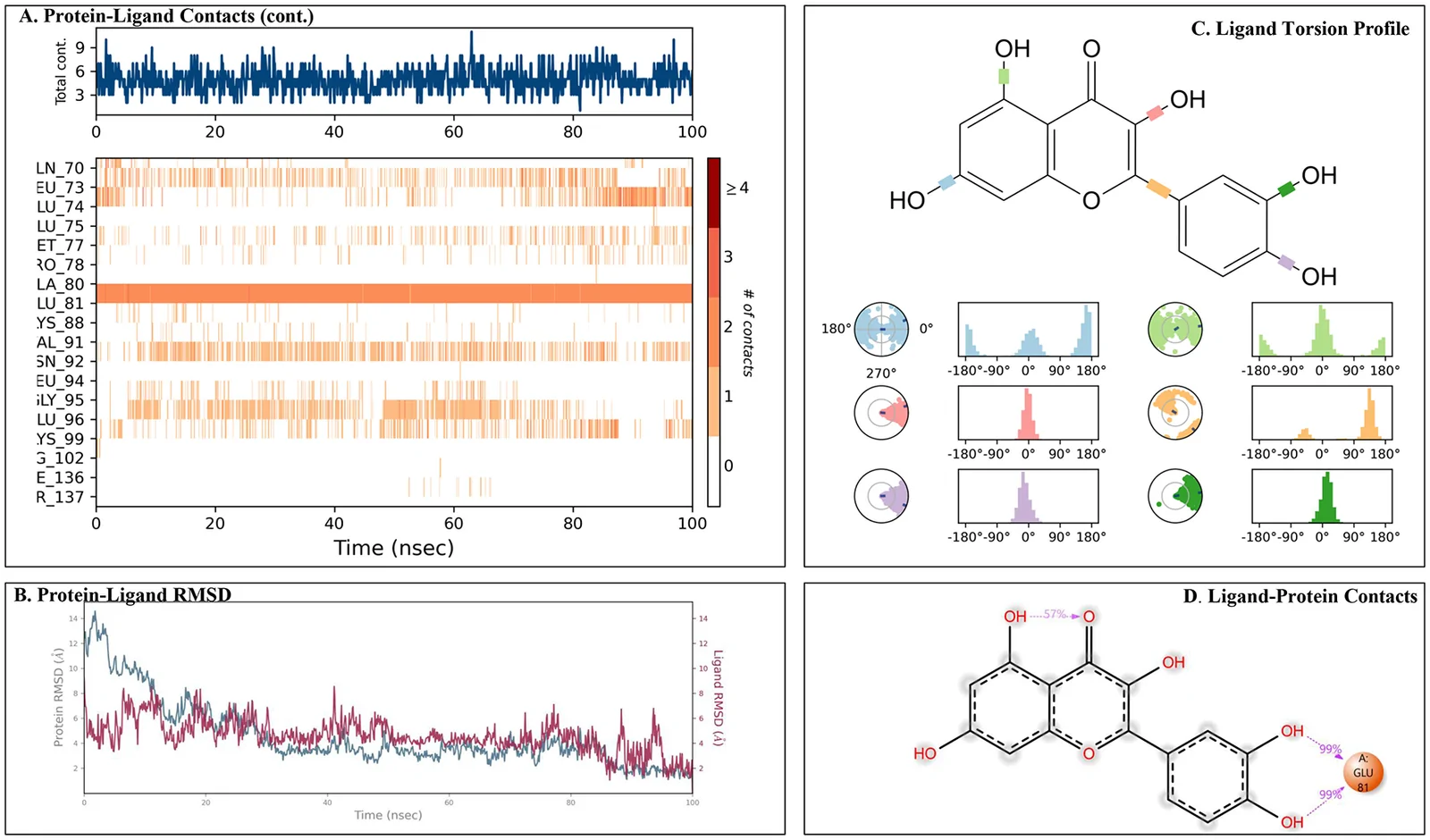
Molecular dynamics simulation and protein-ligand interaction analysis. **(A)** Protein-ligand contact profile over a 100-ns molecular dynamics (MD) simulation, showing the frequency and persistence of interactions between the ligand and key amino acid residues. **(B)** Protein and ligand RMSD profiles demonstrating the structural stability and conformational behavior of the complex throughout the simulation. **(C)** Ligand torsion profile illustrating the conformational flexibility of the ligand during the simulation. **(D)** Two-dimensional protein-ligand interaction diagram showing the major intermolecular contacts and their interaction frequencies, with GLU81 representing a prominent and persistent interaction site. Together, these analyses demonstrate the stability, conformational dynamics, and residue-level interaction pattern of the protein-ligand complex during the 100-ns MD simulation.

The molecular dynamics (MD) analysis presented in the figure illustrates the stability, interaction patterns, and conformational behavior of the ligand–protein complex over 100 ns of simulation. Figure 3 displays the timeline and frequency of ligand–residue contacts, revealing persistent interactions with key residues, such as L-alanine (ALA80) and L-glutamic acid (GLU8). These residues exhibited high contact occupancy, indicating their central role in stabilizing the ligand within the binding pocket. Fluctuating contact profiles across leucine (LEU73), glutamic acid (GLU74), and lysine (LYS99) suggest transient but supportive interactions that contribute to the overall stability. Additionally, the Protein-Ligand Root Mean Square Deviation (RMSD) maintained a low and stable profile across the full simulation time, confirming that the docked pose was persistent and the complex was structurally stable (Figure 3). Analysis of the interactions showed that binding was predominantly stabilized by a high fraction of hydrophobic interactions and several hydrogen bonds, indicating a specific and strong directional fit within the binding pocket. Key residue contacts were sustained throughout the trajectory. The role of MD in docking is critical for dynamic validation. While docking predicts a single static binding pose, the MD simulation confirms that this initial pose is thermodynamically stable and viable over time in a near-physiological environment. This dynamic confirmation is essential to ensure that quercetin does not drift or dissociate, thereby confirming its robust inhibitory potential in colon cancer cells. Throughout the simulation, the interactions between the protein and the ligand were observed. These interactions are classified, as depicted in the plot Figure 3. Protein-ligand interactions, also known as “contacts,” are divided into four categories: hydrogen bonds, hydrophobic, ionic, and water bridges. Each category includes more detailed subtypes, which was examined through the simulation interactions diagram' in Figure 3. The stacked normalized data over the trajectory indicates, for instance, that a value of 0.7 means the specific interaction is sustained for 70% of the simulation time.

### 3.6 Cell cytotoxicity assay

**Figure 4:**
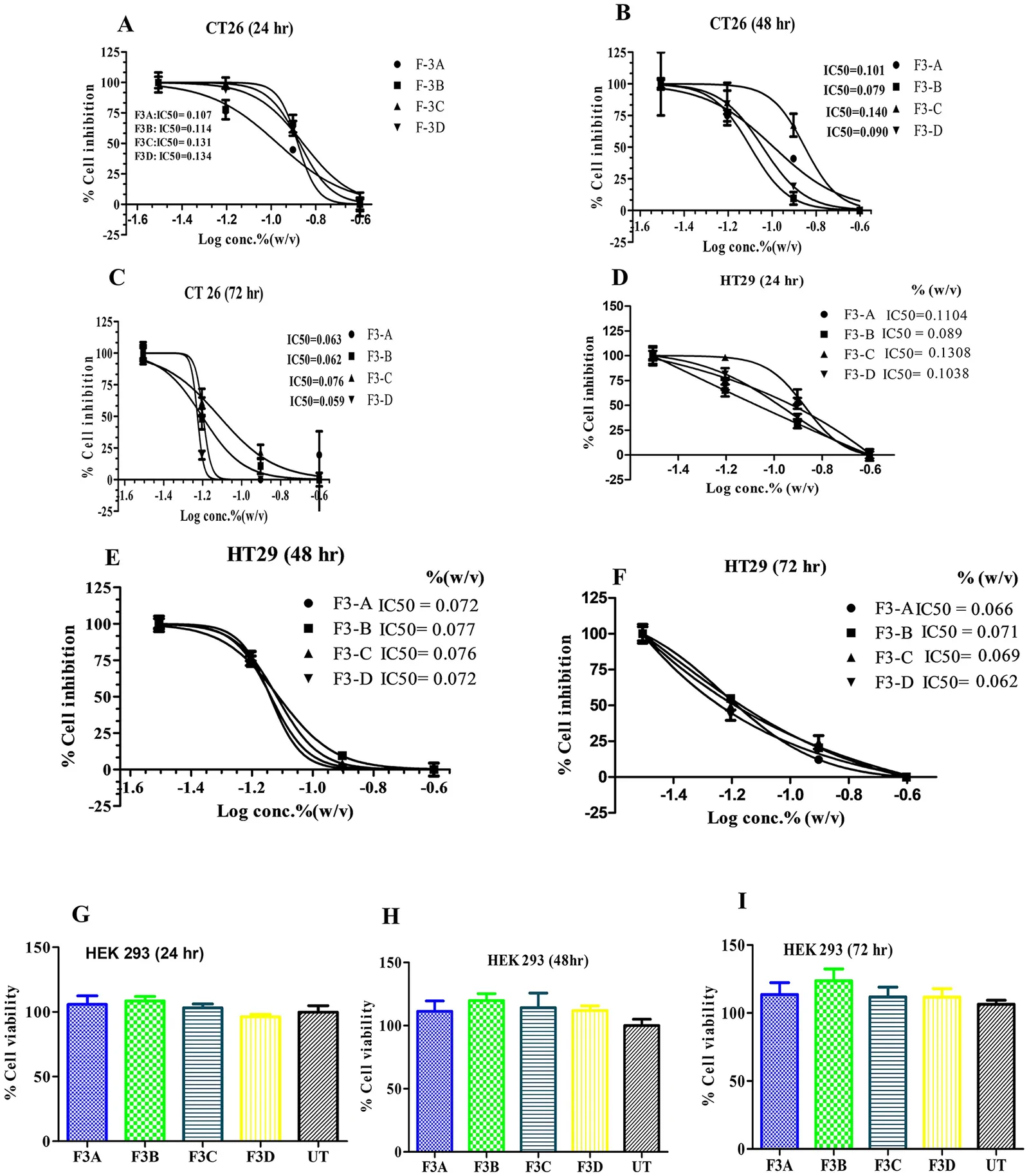
The depicted graphs illustrates the cytotoxic activity of the four formulations (F3A, F3B, F3C, and F3D) against CT26 cell line and human colorectal adenocarcinoma cells (HT29), as well as their effects on non-cancerous human embryonic kidney cells (HEK-293), as determined by MTT assay at 24, 48, and 72 h. **(A–C)** Depict the dose- and time-dependent reduction in cell viability of CT26 cells, while **(D–F)** show a similar inhibitory trend in HT29 cells. In contrast, **(G–I)** demonstrate that none of the formulations significantly affected HEK-293 cell viability, which remained comparable to the untreated control. Interesting all the formulations shown cytotoxicity effect on colon cancer cells and heling effect on normal cells. Statistical analysis was performed using nonlinear fit by GraphPad Prism software. Data represent the mean ± SEM (*n* = 3 independent experiments).

Cytotoxicity analysis of the four polyherbal formulations (F3A to F3D) demonstrated potent and inhibitory effects against the colorectal cancer cell line CT26 (murine colon) and human colorectal adenocarcinoma (HT-29). All formulations were exhibited significant concentration and time-dependent reductions in cell viability in both cancer lines. Notably, formulation F-3B consistently produced the strongest anti-proliferative effect, achieving the lowest IC_50_ value against CT26 cells at 72 h (IC_50_ = 0.062% w/v), closely followed by F-3A (Figures 4A–C). Additionally, all most similar pattern followed for HT-29 cell lines (Figures 4D–F). Importantly, none of the formulations induced cytotoxicity in non-cancerous human embryonic kidney cells (HEK-293), which maintained nearly 100% viability even at the maximum tested concentration (0.250% w/v), comparable to that of the untreated control (UT). These findings confirmed the selective toxicity of the formulations toward malignant cells without affecting normal cells (Figures 4G–I).

**Figure 5:**
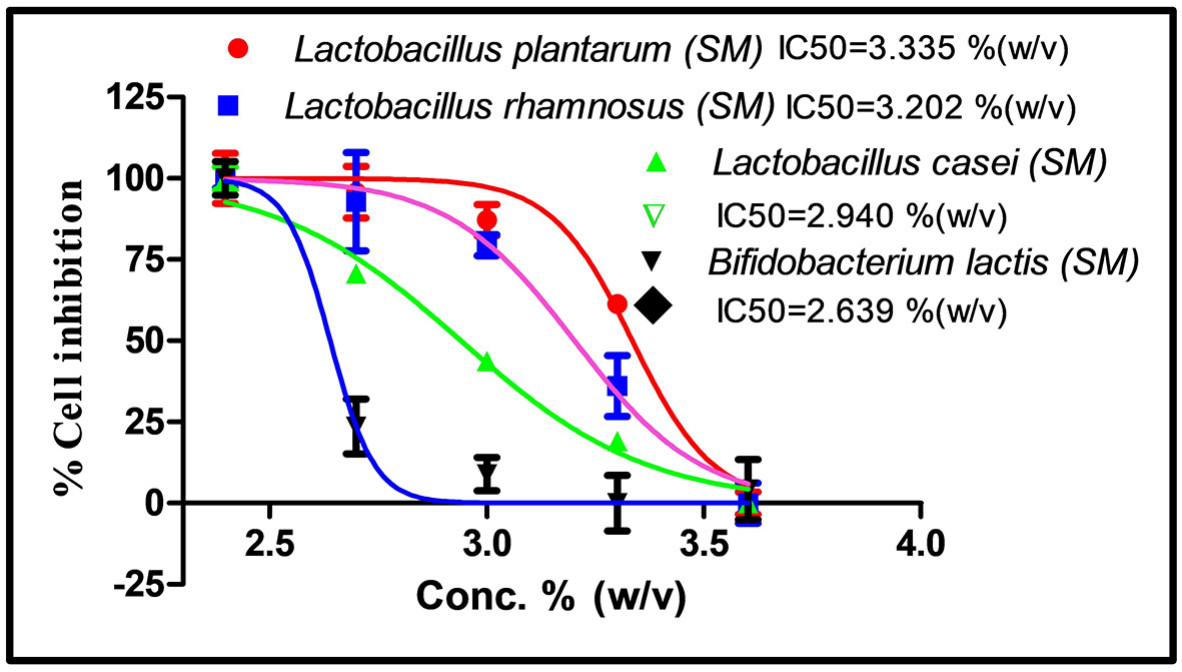
Dose-response inhibition of cell proliferation by probiotic-derived SM on CT26. The graph depicts concentration-dependent inhibitory effect (shown as % Cell Inhibition) of extracts (SM, likely supernatant or media) from four probiotic strains: *Lactobacillus plantarum* (red circle), *Lactobacillus rhamnosus* (blue square), *Lactobacillus casei* (green upright triangle), and *Bifidobacterium lactis* (black inverted triangle). Statistical analysis was performed using nonlinear fit by GraphPad Prism software, and experiment were repeated (*n* = 3) data are reported mean ± SEM.

Subsequently, the cytotoxic potential of the probiotic-derived secondary metabolites was evaluated in CT26 cells. At higher concentrations, these metabolites exhibited dose-dependent anti-proliferative effects, with IC50 values of 3.335% (w/v) for *L*. *plantarum*, 3.202% (w/v) for *L. rhamnosus*, 2.94% (w/v) for *L. casei*, and 2.639% (w/v) *for B. lactis*. Among these*, B. lactis* demonstrated the most pronounced inhibitory activity (Figure 5). Furthermore, combination studies of plant-nutrition-F3B (PN = F3B) and probiotic-derived secondary metabolites (SM) have revealed enhanced cytotoxicity against CT26 cells, depending on the SM:PN ratio. The SMs from *L. plantarum* and *L. rhamnosus* exhibited optimal activity at a 1:1 (SM:PN) ratio, whereas those from *L. casei* and *B. lactis* showed maximal inhibition at a 1:2 ratio. These ratios represent the most effective concentrations for suppressing colon cancer cell proliferation (Supplementary Figures S8A–D).

### 3.7 Apoptosis study flow cytometric

**Figure 6:**
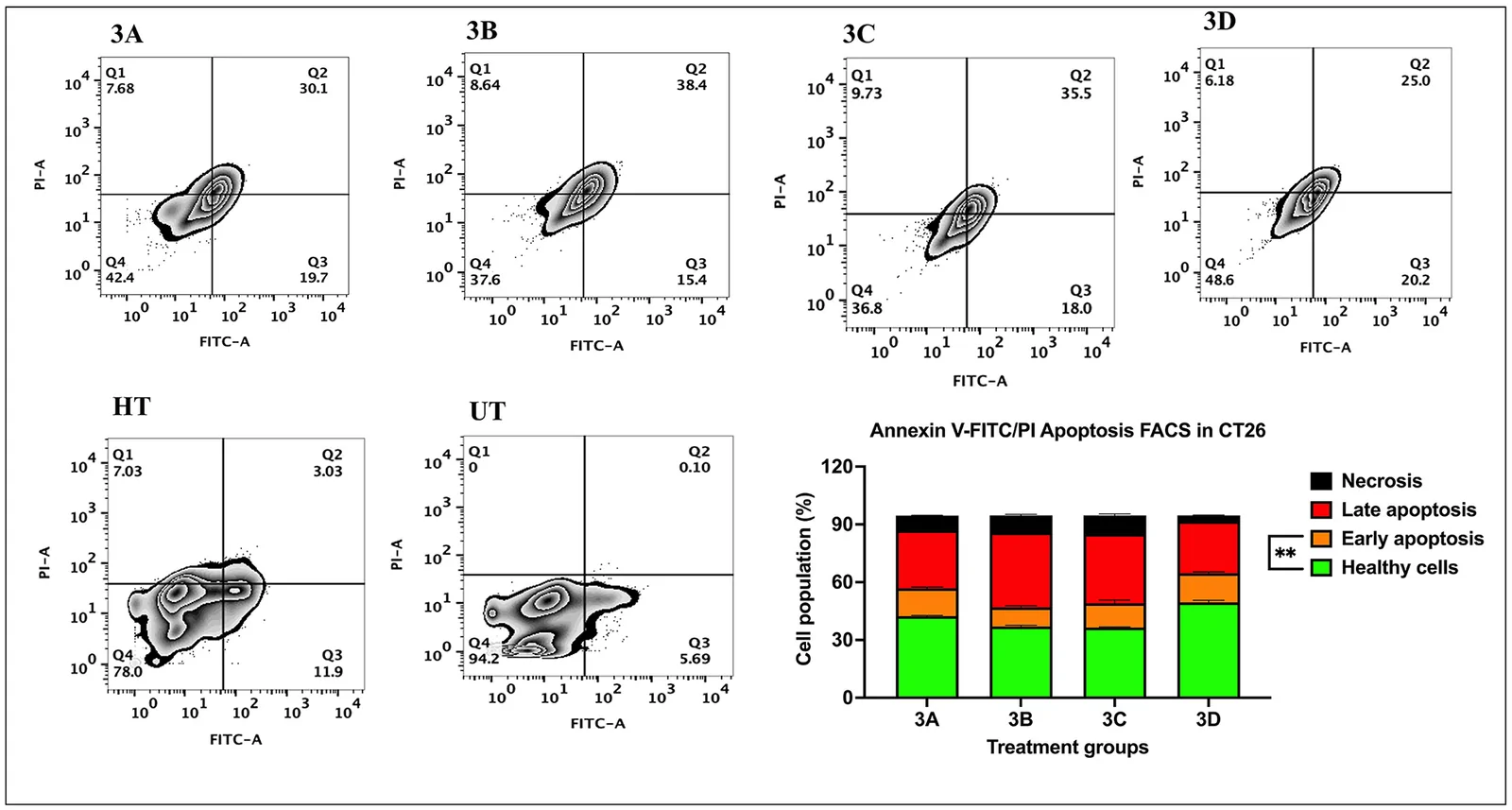
Flow cytometric analysis of apoptosis in CT26 Cells by Annexin V-FITC/PI assay. Flow cytometry quadrant plots (top and bottom left) and quantification bar graph (bottom right) depicts the effect of various treatments on CT26 cell viability. Cells were stained with Annexin V-FITC and Propidium Iodide (PI) to distinguish between cell populations. Untreated (UT), heat-treated (HT @ 60 °C), and experimental formulations F3A, F3B, F3C, and F3D. The bar graph illustrates a significant increase in total apoptosis (blue) in groups F3A–F3D compared to the UT control, while HT shows a distinct increase in necrosis (red). Data are presented as the mean percentage of the cell population ± SEM.

Following the observed inhibitory effects of the developed formulations on CT26 cell proliferation and recognizing that the anticancer activity predominantly arises from the phytonutrient components, further investigation was conducted to determine whether lower plant nutrition (PN) concentrations could induce apoptosis within a shorter incubation period. Quantification of apoptosis via Annexin V/PI flow cytometry demonstrated significant differences in cell death profiles across the treatment groups, with the results for all formulations (F3A, F3B, F3C, and F3D,) clearly summarized Figure 6. Formulation F3B elicited the strongest apoptotic response, with a total apoptotic cell population of 57.33%, which was statistically significant (*P* < 0.01; Supplementary Table S3). Representative bar plots showed a marked increase in the population of apoptotic cells (upper-right quadrants) following treatment with PNs from the formulations F3A-F3D, while untreated cells displayed minimal apoptosis. Notably, F3B and F3C exhibited the highest apoptosis-inducing potentials. Together, the formulations comprising the selected PNs induced apoptosis in the CT26 carcinoma cells Supplementary Table S3).

### 3.8 Proteomics study (western blot analysis)

**Figure 7:**
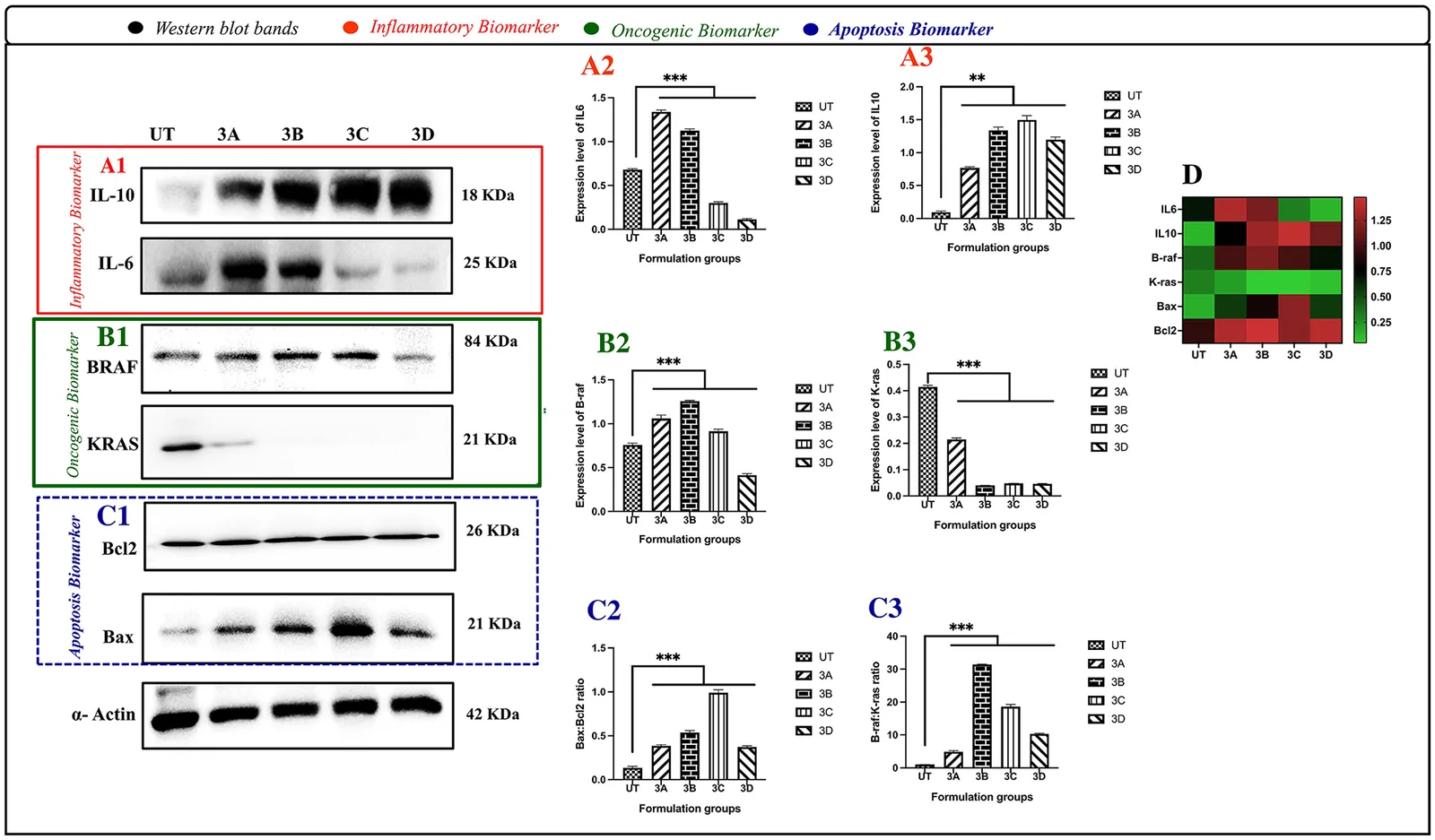
Western blot analysis of apoptotic & tumor marker proteins in CT26 colon cancer cells treated with plant formulations (F3A–F3D). **(A)** Shows the expression of cytokines IL-10 and IL-6 following treatment with formulations F3A–F3D. Treatment resulted in significant upregulation of the anti-inflammatory cytokine IL-10 and concurrent downregulation of the pro-inflammatory cytokine IL-6 after treatment with F3C and F3D compared to the untreated control, indicating modulation of inflammatory signaling. **(B)** Shows the tumor-associated markers BRAF and KRAS. Formulations F3A–F3D significantly downregulated KRAS oncogenic proteins, whereas BRAF was not effectively downregulated by the formulation (F3A–F3C), but F3D showed minimal downregulation. **(C)** Shows the apoptosis-related proteins Bax and Bcl-2. Treatment induced a marked increase in Bax expression and a decrease in Bcl-2 expression, leading to an elevated Bax/Bcl2 ratio, indicative of enhanced apoptotic activity. **(D)** The heatmap illustrates the expression levels of all the biomarkers, with deep red indicating upregulation and deep green downregulation. Densitometric quantification of all protein bands was performed using ImageJ software, normalized to actin, and expressed as mean ± SEM (*n* = 2 independent experiments). Data are presented as mean ± SEM (*n* = 3 independent experiments).

To elucidate the molecular mechanism of action, key protein markers were analyzed in CT 26 cells following treatment with the formulation. Western blot analyses focused on apoptotic (BAX and BCL2), oncogenic (K-RAS and B-RAF), and immunomodulatory (IL-6 and IL-10) markers. The results demonstrated differential regulation of immune-associated cytokines following treatment. IL-10 expression was significantly increased, while IL-6 expression was also elevated in some formulation groups, particularly F3A and F3B (Figures 7A1–A3). Because IL-6 is a pleiotropic cytokine involved in both inflammatory and immune regulatory processes, these findings are interpreted as evidence of cytokine modulation rather than direct anti-inflammatory activity. Conversely, the expression of oncogenic proteins, K-RAS and B-RAF, was markedly downregulated (Figures 7B1–B3), indicating the suppression of tumor progression. Additionally, a higher BAX/BCL2 ratio was observed, characterized by a significant increase in BAX expression with relatively stable BCL-2 levels (Figures 7C1–C3). This shift toward pro-apoptotic signaling suggests the activation of the intrinsic mitochondrial pathway, promoting cytochrome c release and caspase-mediated apoptosis. Overall, western blot analyses confirmed that the formulation F3B effectively downregulated oncogenic markers, enhanced apoptosis, and modulated inflammatory signaling, demonstrating strong therapeutic potential for managing colon cancer and gut dysbiosis. The heatmap illustrates the expression levels of all the biomarkers, with deep red indicating upregulation and deep green downregulation.

### 3.9 Inhibits *E. coli* growth *in-vitro*

**Figure 8:**
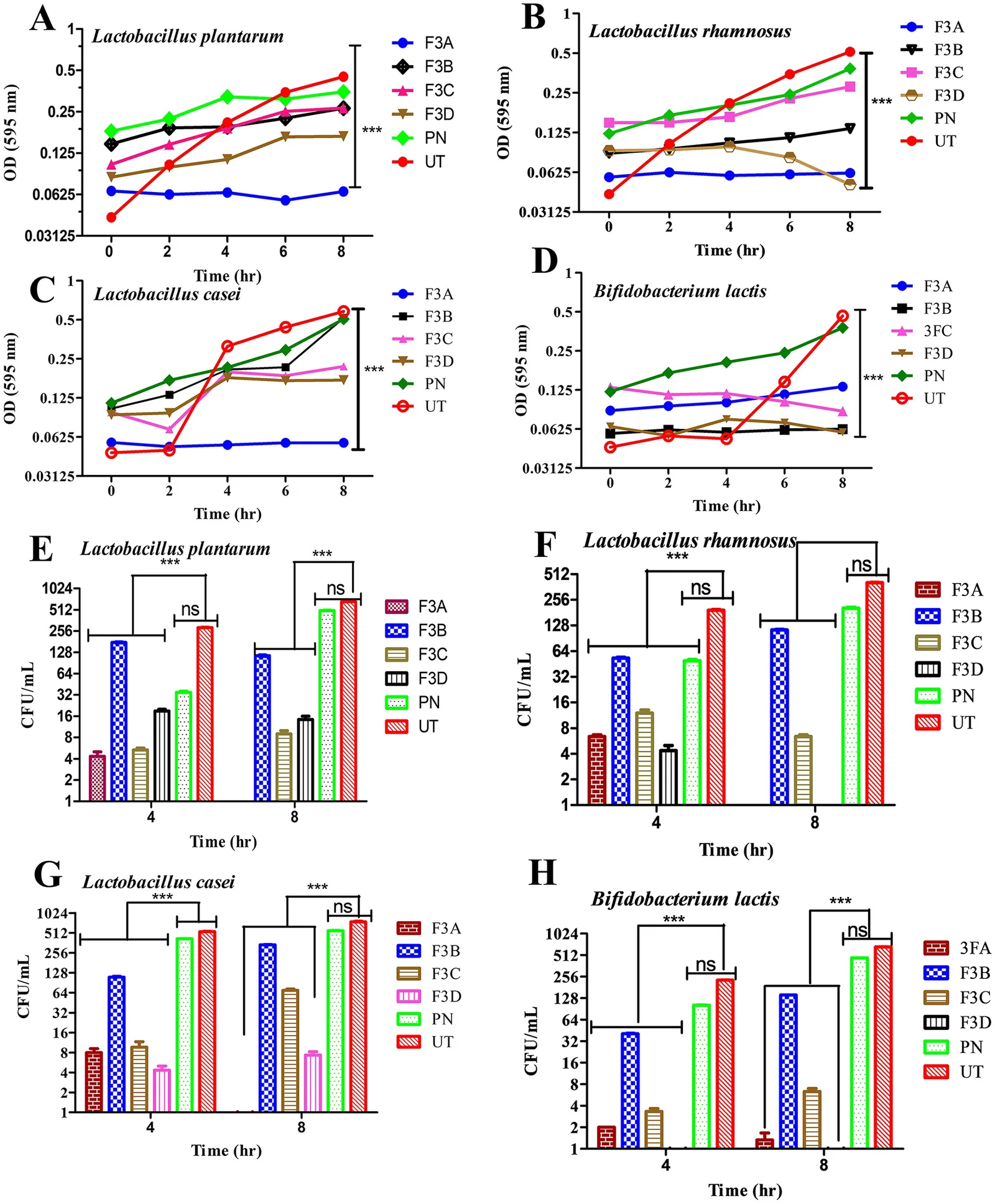
Growth-promoting effects of four polyherbal formulations (F3A–F3D) on probiotic bacterial strains. **(A–D)** Growth kinetics of *Lactobacillus plantarum, Lactobacillus rhamnosus, Lactobacillus casei*, and *Bifidobacterium lactis*, respectively, determined by optical density (OD at 595 nm) at different incubation time points (0–8 h). **(E–H)** Corresponding viable bacterial counts (CFU/mL) at 4 and 8 h demonstrating the influence of each formulation on probiotic proliferation. Data are presented as mean ± SEM from three independent experiments (*n* = 3). Statistical significance was determined using one-way ANOVA followed by Tukey's multiple comparison test, whereas * = *P* < 0.05; ** = *P* < 0.01; *** = *P* < 0.001; and not significant (ns) *P* > 0.05.

To evaluate the role of F3B in ameliorating gut dysbiosis associated with CRC, its antimicrobial potential was examined using *an in-vitro E. coli* model. Secondary metabolites (SMs) from four probiotic strains, namely, *L. plantarum, L. rhamnosus, L. casei*, and *B. lactis*, were isolated and quantified. These SMs were combined with phytonutrients (PNs) of F3B at varying concentration ratios and tested against *E. coli* cultures at dilutions of 10^−2^ and 10^−4^. Bacterial growth and survivability were assessed spectrophotometrically by measuring the optical density at 600 nm, and CFU/mL counts were determined at dilutions of 10^−6^ and 10^−8^ (Figure 8; Supplementary Figure S9). Treatment with probiotic-derived SMs alone resulted in the complete inhibition of *E. coli* growth, whereas untreated controls showed normal proliferation. In contrast, PNs alone had a minimal effect on bacterial growth, and mixed SM-PN treatment produced intermediate inhibition. These results indicate that the antimicrobial activity of F3B primarily stems from probiotic-derived SMs. Overall, the inclusion of four probiotic strains in F3B effectively suppressed *E. coli* growth *in-vitro*, suggesting its potential role in modulating the gut microbiota and preventing dysbiosis-linked colorectal carcinogenesis.

### 3.10 Splenocyte proliferation assay

**Figure 9:**
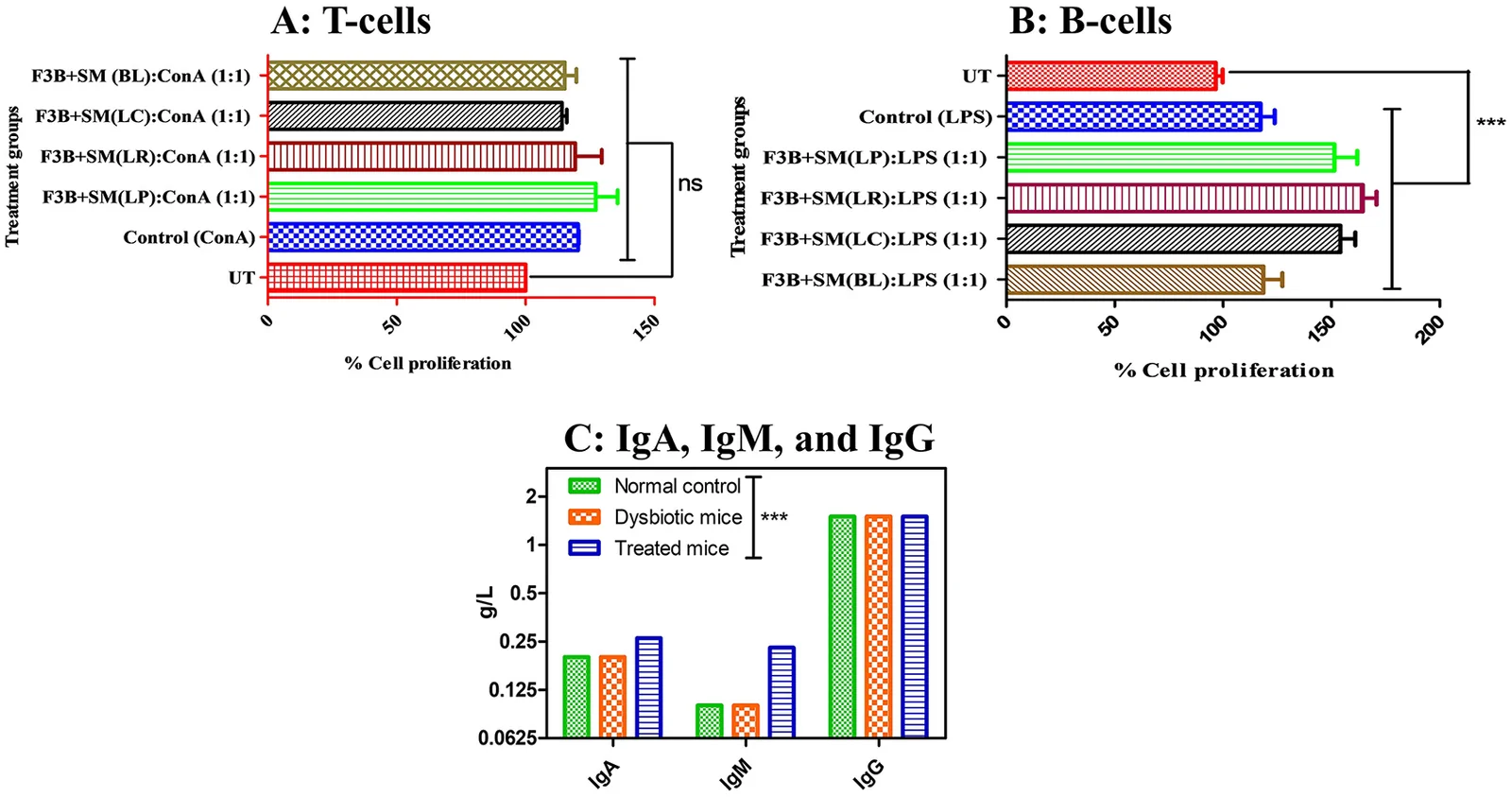
Immunomodulatory activity of the optimized polyherbal formulation (F3B) supplemented with probiotic-derived soluble metabolites (SMs). **(A)** *Ex-vivo* T-cell proliferative response of murine splenocytes stimulated with concanavalin A (ConA) following treatment with F3B combined with soluble metabolites derived from *Lactobacillus plantarum (LP), Lactobacillus rhamnosus (LR), Lactobacillus* casei *(LC)*, and *Bifidobacterium lactis (BL)* at a 1:1 ratio. **(B)** *Ex-vivo* B-cell proliferative response following lipopolysaccharide (LPS) stimulation under identical treatment conditions. **(C)** *In-vivo* serum immunoglobulin concentrations (IgA, IgM, and IgG) in normal control, dysbiotic, and F3B-treated mice, demonstrating restoration of humoral immune function following treatment. Data are presented as mean ± SEM (*n* = 3 independent experiments). Statistical analysis was performed using two-way analysis of variance (ANOVA) followed by Tukey's multiple comparisons test using GraphPad Prism. Statistical significance is indicated as *P* < 0.05 (*), *P* < 0.01 (**), and *P* < 0.001 (***), whereas ns denotes no statistically significant.

The T-cell proliferation assay (Figure 9A) shows that all treatment groups specifically the combinations involving various *Lactobacillus* strains like LP, LR, LC, and BL maintained consistent proliferation levels compared to the ConA control. The lack of statistical significance (ns) across these groups suggests that the nutraceutical treatment does not suppress the cellular immune response, maintaining homeostatic T-cell activity. In contrast, the B-cell proliferation results (Figure 9B) demonstrate a significant enhancement. The treatment groups (F3B+SM) induced a markedly higher percentage of cell proliferation compared to both the untreated (UT) and LPS control groups (*P* < 0.001). This indicates that the F3B+SM formulation acts as a potent stimulator of humoral immunity, potentially boosting the host's ability to mount an antibody-mediated defense against tumor progression. Figure 9C highlights the impact on systemic immunity through IgA, IgM, and IgG levels. While IgG levels remained stable across all groups, there is a visible increase in IgA and IgM concentrations in the Treated mice compared to the dysbiotic and normal control groups (*P* < 0.001). The elevation of IgA is particularly relevant for colon cancer, as it strengthens the mucosal barrier. These results collectively suggest that the poyherbal treated groupes facilitates a robust immune microenvironment, characterized by enhanced B-cell activation and increased mucosal antibody production, which are critical for inhibiting colorectal tumor growth.

### 3.11 Hematological test (RBC count, hemoglobin, neutrophils, lymphocytes)

#### 3.11.1 WBC count

**Figure 10:**
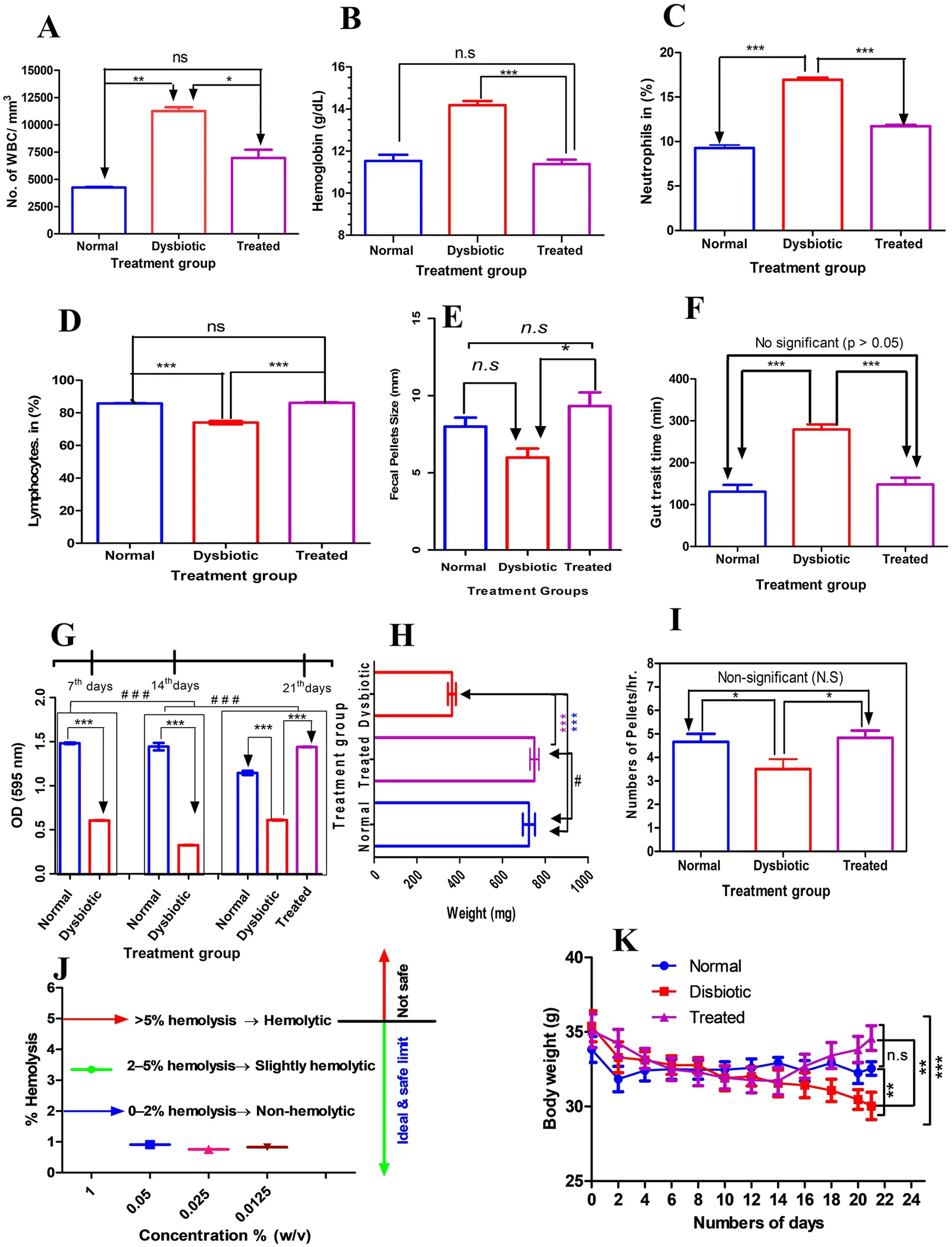
Remodulation of gut dysbiosis and restoration of hematological and gastrointestinal parameters in normal, dysbiotic, and treated mice. This figure illustrates the ameliorative effects of the treatment formulation on a murine model of gut dysbiosis across hematological and gastrointestinal systems. Hematological analyses demonstrated that treatment significantly increased the white blood cell (WBC) count **(A)** and hemoglobin (Hb) levels **(B)** while restoring the percentages of neutrophils **(C)** and lymphocytes **(D)** to the values observed in the normal control group, thereby reversing the dysbiosis-induced alterations. Gastrointestinal function was evaluated based on fecal pellet size **(E)** and whole-gut transit time **(F)**. Dysbiotic mice exhibited smaller fecal pellets and markedly prolonged transit times, both of which normalized following treatment, indicating improved gut motility and functions. Additionally, treatment enhanced overall gut health by significantly increasing bacterial diversity, as measured by optical density at 600 nm **(G)**, over a 21-day period compared to the dysbiotic group. Pellet weight **(H)** and pellet discharge frequency **(I)** were restored to control levels. Hemolysis testing **(J)** confirmed that the formulations were non-hemolytic and safe for consumption. Body weight monitoring throughout the study period **(K)** revealed that the treated mice maintained a stable weight, preventing the significant weight loss observed in dysbiotic animals. All the experiments repat (*n* = 3). Statistical analysis was performed using two-way analysis of variance (ANOVA and *t*-test) with GraphPad Prism software. Significance levels are indicated as *, ** & *** respectively. ### are used to indicate significance relative to a different groups at different time interval 7th, 14th, and 21th days. ns, not significant.

The total number of White Blood Cells (WBCs) showed a dramatic inflammatory response induced by gut dysbiosis. Normal mice maintained a low baseline WBC count (approximately 4,000 WB\Cumm). In contrast, dysbiotic mice exhibited a highly significant increase (*P* < 0.001), increasing the WBC count to approximately 110,000. This elevation was indicative of a profound systemic inflammatory state. The Treatment group had a significantly lower WBC count than the dysbiotic group (*P* < 0.05), lowering it to approximately 70,000 WBC/cumm. While this represents a successful anti-inflammatory effect, the count remained above the normal baseline; however, it indicates that the proposed formula (F3B) could be used to ameliorate gut hemostasis (Figure 10A).

#### 3.11.2 Hemoglobin

The hemoglobin (HB) concentration (g/dL) was significantly altered in the dysbiosis group. Both the normal and treated mice maintained similar, statistically non-significant (ns) HB levels, averaging approximately 11.5 g\dL. However, dysbiotic mice showed a highly significant elevation in HB (*P* < 0.001), reaching approximately 14.2 g/dL compared to both the normal and treated groups. This perturbation in HB levels could be related to complex inflammatory or fluid dynamic changes associated with severe dysbiosis. The therapeutic intervention of F3B completely protected the treated mice, indicating that thetreatment effectively preserved hematological balance (Figure 10B).

#### 3.11.3 Neutrophils

The percentage of circulating neutrophils, a critical marker of acute and chronic inflammation, is significantly affected by dysbiosis. Normal mice maintained a baseline of 9.3%. The dysbiotic group displayed significant alterations in neutrophilia (*P* < 0.001), reaching 16.9% ± 0.599%. Other hand, treatment group significantly reduced the percentage of neutrophils (*P* < 0.001 vs. the dysbiotic group) to 11.7% ± 0.402%. However, elevated neutrophil levels in the dysbiotic groups caused chronic inflammation, leaky gut, and amplified cytokine release, although F3B intervention, the level of neutrophils, confirming that the proposed formula could be used for the management of chronicinflammation and gut homeostasis (Figure 10C).

#### 3.11.4 Lymphocytes

In contrast to neutrophils, the lymphocyte percentage exhibited a significant decrease (lymphopenia) in the dysbiotic group, falling to approximately 73% from the baseline of approximately 86% in the normal group (*P* < 0.001). This relative lymphopenia is often accompanied by a severe inflammatory state. Crucially, the Treatment group successfully restored the lymphocyte percentage to levels that were not statistically significant (ns) from those of the normal group, maintaining a value of approximately 86%. This full restoration suggests that the treatment effectively reversed the immune suppression orredistribution caused by gut dysbiosis (Figure 10D).

#### 3.11.5 Modulation of gut dysbiosis to symbiotics

As discussed in the above section, we successfully developed a gut dysbiosis model in BALB/c mice by the continuous administration of antibiotics till 14th day.

#### 3.11.6 Body weight variation

All experimental groups started with comparable initial body weights (day 0). Over the 22-day observation period, the dysbiotic group exhibited a progressive decrease in body weight, resulting in statistically significant weight loss compared to the normal control group at the endpoint (*P* < 0.01). Conversely, mice in the treated (F3B) group initially decreased in weight up to the 14th day, and thereafter, increased in body weight throughout the experiment. The body weight of the forulation treated group was significantly higher than that of the dysbiotic group (*P* < 0.001) and showed no significant difference (ns) when compared to the normal group, indicating that our formulation treatment effectively prevented dysbiosis-associated weightloss (Figure 10K).

#### 3.11.7 Pellet size

While the pellet Size: While the Dysbiotic group was slightly smaller than that of the normal group, this difference was not significant (ns). However, the F3B treatment significantly increased the pellet size in the treated group compared to the dysbiotic group (*P* < 0.05), suggesting an improvement in fecal bulk and consistency (Figure 10E). The collected pellet size representative image is shown in Supplementary Figure S10.

#### 3.11.8 Whole gut transit time

Gut dysbiosis significantly impaired intestinal motility, as evidenced by a substantial increase in Whole Gut Transit Time (WGTT) in the dysbiotic group compared with that in the normal group (*P* < 0.001). Treatment with probiotic and prebiotic-based immunostimulatory formulation (F3B) was highly effective in restoring motility, causing a significant reduction in WGTT compared to the dysbiotic group (*P* < 0.001). The WGTT in the Treated (F3B) group was comparable to that in the normal control group, indicating complete restoration of normal intestinal transit speed (Figure 10F).

#### 3.11.9 Modulation of gut dysbiosis to symbiotic by spectroscopic method

The normal control group maintained consistently high OD values throughout the study, ranging from approximately 1.45 at day 7 to 1.15 at day 21, indicating a stable and dense microbial population, which was further confirmed by FE-SEM analysis. In contrast, the dysbiotic control group exhibited significant reduction in OD at all time points (*P* < 0.001), with values remaining low (~0.55 at day 7 and 0.57 at day 21), confirming a persistent state of microbial depletion and dysbiosis. Following treatment with F3B, a remarkable restoration of microbial growth was observed by day 21. The treated group displayed a significant increase in OD (~1.40) compared with the dysbiotic control group (~0.57) (*P* < 0.001). The restored OD values were statistically indistinguishable from those of the normal control, demonstrating that F3B effectively restored gut microbial biomass and diversity to symbiotic and homeostatic levels (Figure 10G).

#### 3.11.10 Pellet weight

The dysbiotic group showed a markedly lower mean pellet weight (in mg) than the normal group, a difference that was highly significant (*P* < 0.001). F3B treatment effectively ameliorated this effect, significantly increasing pellet weight in the Treated (F3B) group compared to that in the dysbiotic group (*P* < 0.001). The final pellet weight in the treated group was restored and was equal to that of the normal group. However, no significant differences were observed. Our findings revealed that the proposed formulation could be used forthe management of gut dysbiosis and associated cancers (Figure 10H).

#### 3.11.11 Number of pellets discharged

The number of pellets discharged was recorded in pellets/h. However, our experimental results revealed that there was no significant difference between the normal and treated group mice, while compared with normal vs. dysbiosis and dysbiosis vs. treated mice, we observed a significant difference (*P* < 0.05^*^). Although the dysbiotic group exhibited a trend toward reduced pellet discharge, and the treated group showed a slight increase, these differences were statistically significant (Figure 10I).

#### 3.11.12 Hemolysis

A safety evaluation assessing hemolysis percentages at different concentrations of the treatment of formulation F3B (ranging from 0.1% to 0.0125% w/v) verified its biocompatibility. All concentrations tested and under 1% of hemolysis was observed. Based on established criteria, a hemolysis value between 0% and 2% is considered non-hemolytic and falls within the “Ideal & safe limit.” This finding confirmed that the prosed formula is safe for systemic use and does not damage red blood cells at the tested concentrations (Figure 10J).

#### 3.11.13 Body weight variation

All experimental groups started with comparable initial body weights (day 0). Over the 22-day observation period, the dysbiotic group exhibited a progressive decrease in body weight, resulting in statistically significant weight loss compared to the normal control group at the endpoint (*P* < 0.01). Conversely, mice in the treated (F3B) group initially decreased in weight up to the 14th day, and thereafter, increased in body weight throughout the experiment. The body weight of the forulation treated group was significantly higher than that of the dysbiotic group (*P* < 0.001) and showed no significant difference (ns) when compared to the normal group, indicating that our formulation treatment effectively prevented dysbiosis-associated weightloss (Figure 10K).

### 3.12 Validation of gut dysbiosis to symbiosis by FE-SEM

**Figure 11:**
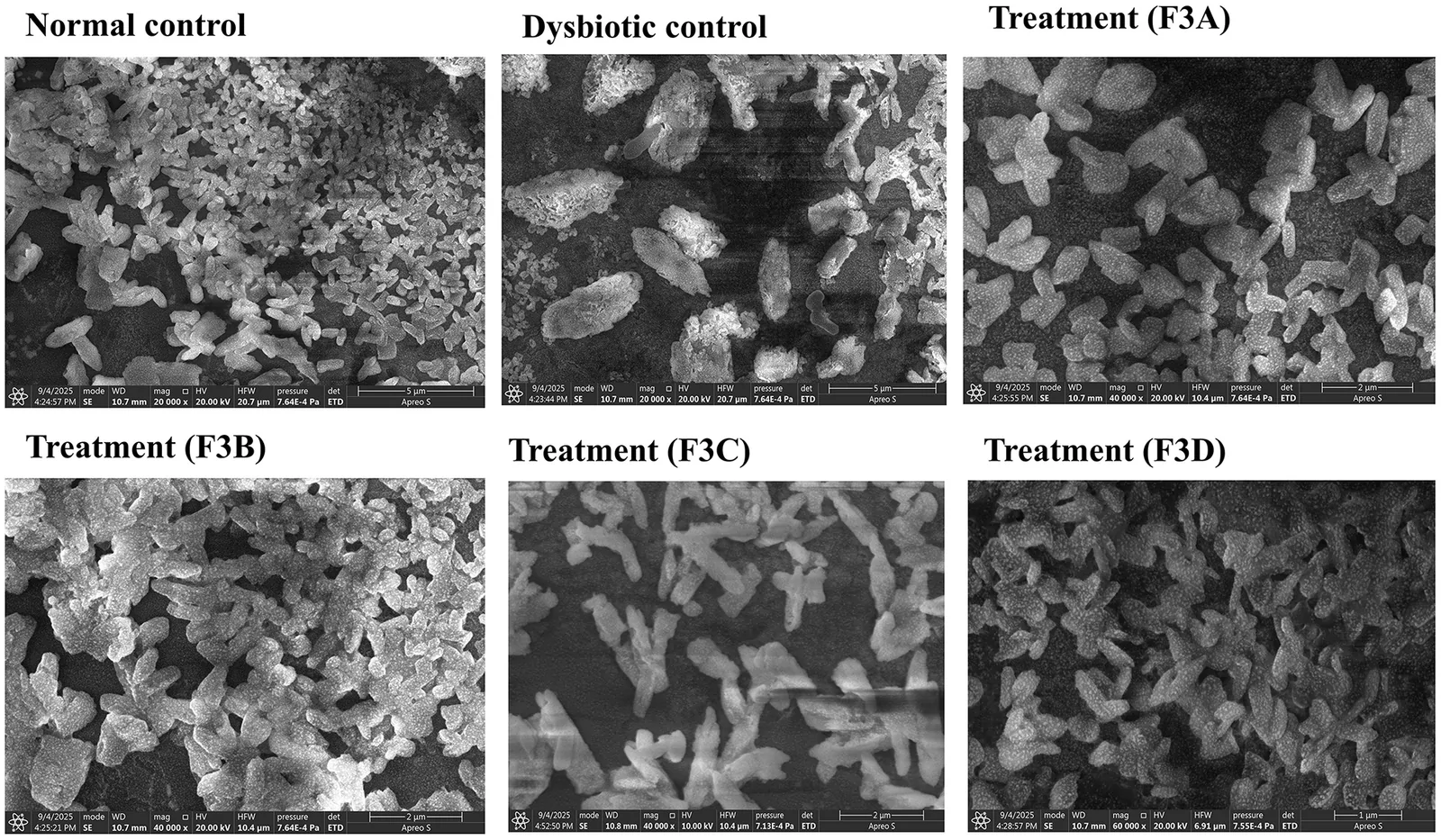
Field-emission scanning electron microscopy (FE-SEM) images showing the effects of polyherbal formulations on the gut bacterial architecture in BALB/c mice. Representative micrographs of the normal control, dysbiotic control, and treatment groups (F3A–F3D) are presented. The normal control group exhibited abundant, densely populated, and morphologically intact bacterial communities, whereas the dysbiotic control displayed a marked reduction in bacterial abundance together with disrupted bacterial morphology and compromised structural integrity. Treatment with formulations F3A–F3D markedly restored bacterial density, preserved cellular morphology, and improved the overall structural organization of the gut bacterial community, with F3B exhibiting the most pronounced restoration, closely resembling the normal control group. The FE-SEM observations provide qualitative visual evidence supporting the ability of the polyherbal formulations to alleviate antibiotic-induced gut dysbiosis and promote recovery of the intestinal microbial ecosystem. Representative images are shown from three independent biological replicates (*n* = 3).

FE-SEM analysis demonstrated the restoration of the gut microbiota in BALB/c mice following treatment with all four formulations **(F3A-D)**. The normal control micrographs (Figure 11, top left) showed a dense, morphologically dense microbial community comprising both Gram-positive and gram-negative bacteria, which is characteristic of a healthy gut (Supplementary Figure S11). Conversely, the dysbiotic control (Figure 11, top right) revealed markedly reduced bacterial density, structural damage, and compromised membrane integrity, which is consistent with severe dysbiosis. In the treated group (Figures 3A–D, 11) significantly restore bacterial diversity was significantly restored in the gut microbiome. In contrast, FE-SEM demonstrated substantial restoration of bacterial density and morphological diversity, closely resembling that of the normal control. These observations provide direct visual and quantitative evidence that F3B treatment effectively reversed gut dysbiosis, restored bacterial diversity, and preserved the structural and functional integrity of the intestinal microbiota. Notably, the microbial profile of the F3B-treated mice was allmost equal bacterial diversity compare to normal control group.

### 3.13 16S rRNA sequencing

**Figure 12:**
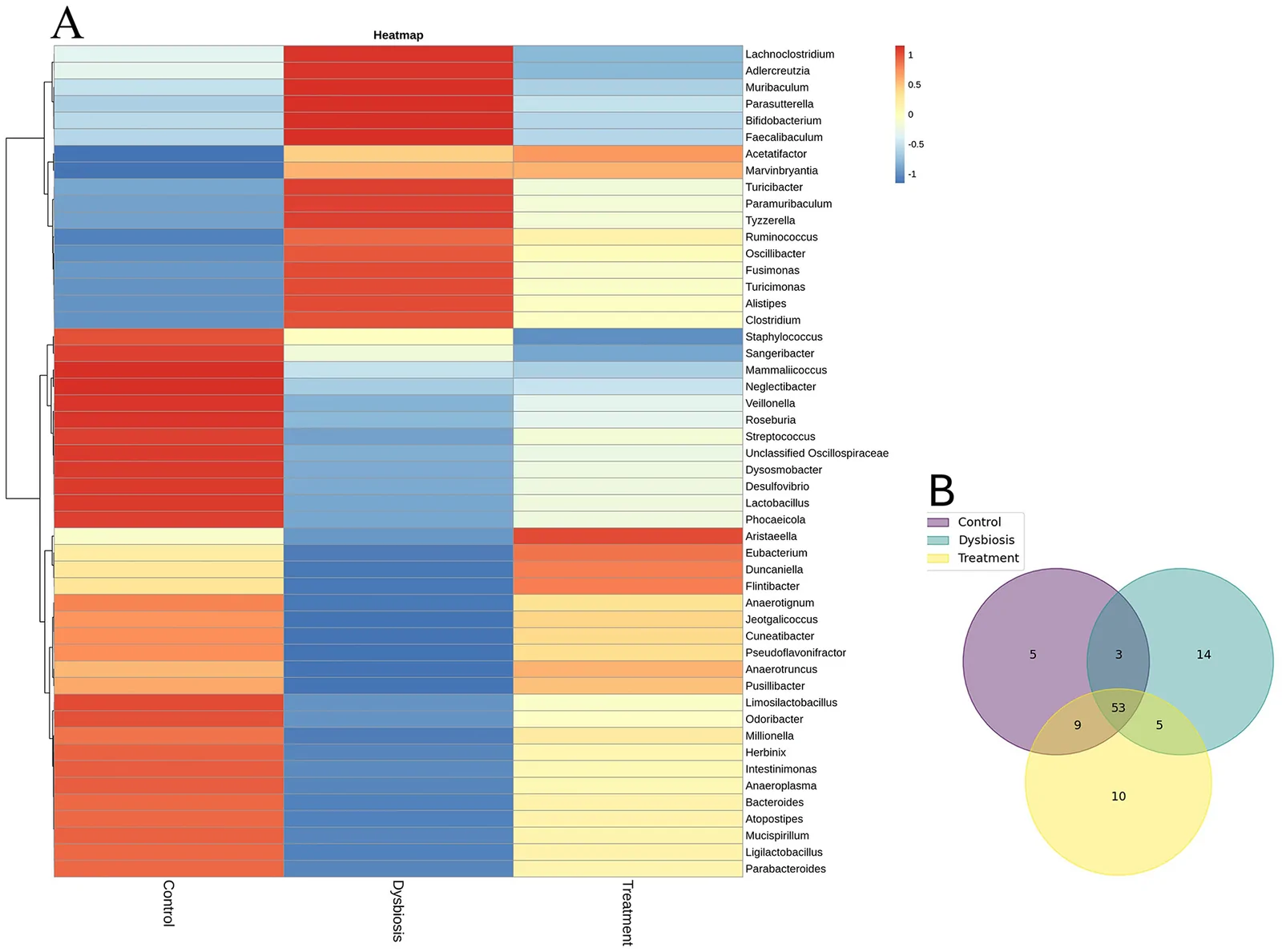
16S rRNA results: **(A)** Heat map: Unit variance scaling was applied to the sequence abundance. Taxon rows were clustered using correlation distance and average linkage. The top 50 genera based on average abundance were considered for heatmap construction**. (B)** Venn diagram: Visually represents the diversity and overlap of bacterial communities (measured as OTUs) identified through 16S rRNA gene sequencing of fecal samples from three experimental groups: control (purple), dysbiosis (teal), and treatment (yellow). A total of 84 unique OTUs were identified across all conditions. The central overlap of the 53 OTUs indicated that the core microbial community was shared by all three groups. The dysbiosis group had the highest number of unique OTUs (14), which were not present in the other groups, potentially representing the bloom species. Conversely, the treatment group showed a significant overlap with the control group (nine OTUs shared only between them) and a smaller set of unique OTUs (10), demonstrating the capacity of the treatment to modulate the gut microbiota toward a profile that shares characteristics with the healthy control state Statistical analysis was performed by origin pro-25 latest version software.

The 16S rRNA gene sequencing results, visualized through a heatmap (A) and Venn diagram (B), confirmed the capacity of the treatment to modulate the gut microbiota composition. The heatmap, based on the top 50 genera, clearly demonstrated that the normal control and treated groups clustered closely together, exhibiting a high relative abundance of beneficial taxa (red/yellow regions). Conversely, the dysbiotic control group displayed a distinct signature characterized by the proliferation of specific bloom species and a significant depletion of many core genera (blue regions) (Figure 12A). Venn diagram analysis of 84 total Operational Taxonomic Units (OTUs) showed a significant overlap of 53 OTUs shared by all three groups. Crucially, the treated group shared nine OTUs exclusively with the normal control, whereas the dysbiotic control group possessed the highest number of unique OTUs (14), indicating a loss of core diversity and expansion of niche taxa. This gene-level evidence confirmed that the treatment successfully shifted the dysbiotic microbial profile toward the profile characteristic of healthy normal controls (Figure 12B).

### 3.14 Gram staining

Gram staining was conducted on bacterial populations from fecal samples of healthy, dysbiotic, and treated mice. Microscopic examination revealed that fecal samples from dysbiotic mice had less bacterial diversity compared to those from treated and normal control mice. Representative microscopic images display the results of gram staining. Fecal smears from healthy mice showed a dense and varied bacterial population, while those from dysbiotic mice had a significantly reduced bacterial load, indicating a depletion of microbiota. In treated mice, the bacterial abundance was nearly restored to normal levels, suggesting a recovery of the intestinal microbial community (Supplementary Figure S11).

### 3.15 Histopathology

**Figure 13:**
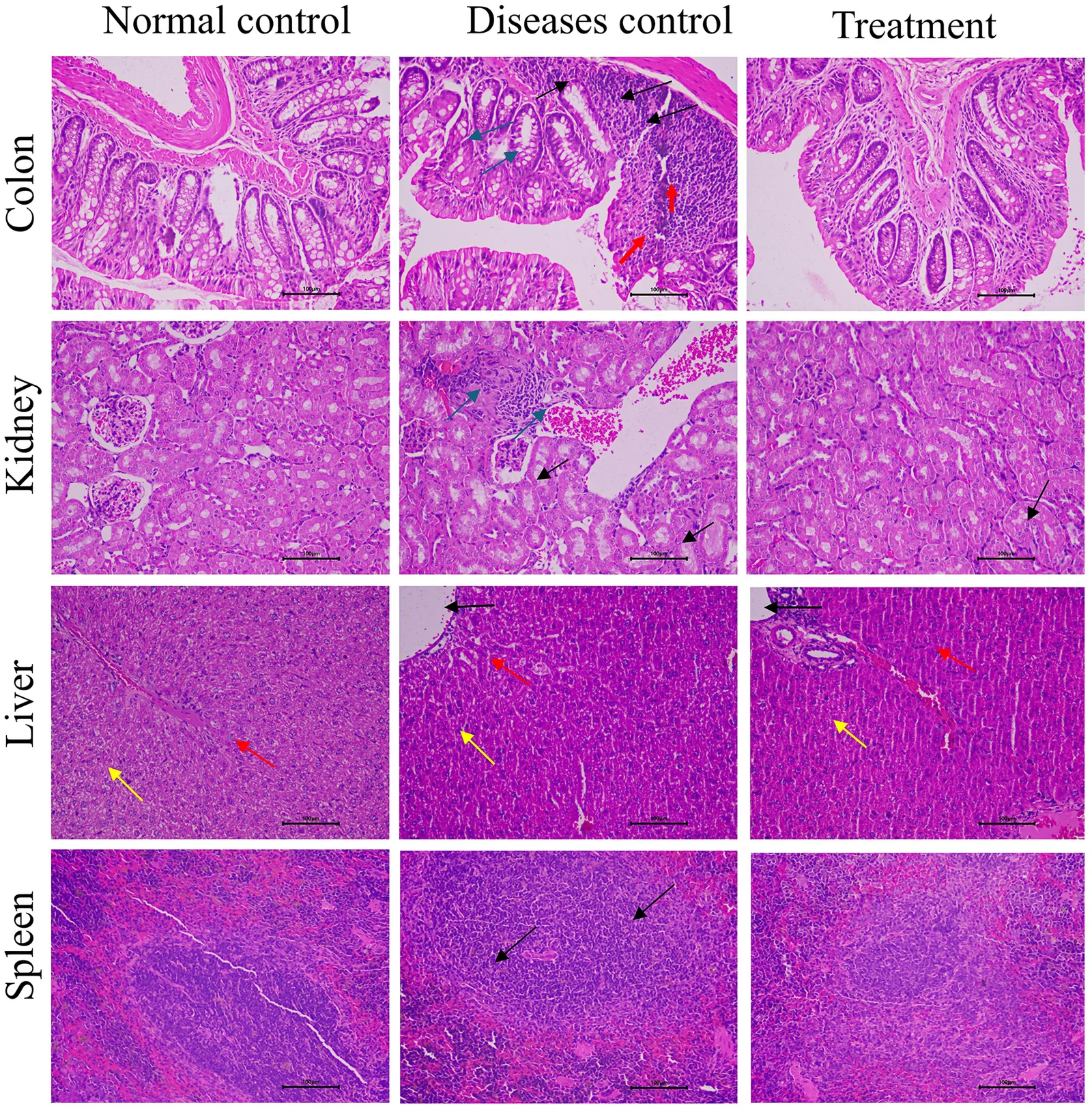
Comparative histopathological assessment of colon, kidney, liver, and spleen by H&E staining. Representative H&E-stained sections from the normal control, disease control, and treatment groups of experimental mice at 20 × magnification (scale bar = 100 μm). The normal control showed preserved tissue architecture, whereas the disease-control group exhibited marked pathological alterations, including inflammatory-cell infiltration, tissue disorganization, crypt/tubular damage, and disruption of hepatic and splenic architecture. The treatment group showed comparatively improved preservation of tissue architecture with reduced pathological changes. In the colon, black arrows indicate crypt disruption, cyan arrows indicate epithelial/inflammatory alterations, and red arrows indicate inflammatory-cell infiltration. In the kidney, cyan arrows indicate inflammatory infiltration and black arrows indicate tubular/glomerular alterations. In the liver, yellow arrows indicate sinusoidal spaces, red arrows indicate hepatic cords/plates, and black arrows indicate architectural alterations. In the spleen, black arrows indicate alterations in white- and red-pulp organization. Histopathological changes were scored as 0 = no effect, 1 = mild, 2 = moderate, and 3 = severe.

Histopathological evaluation was conducted on the H&E-stained colon, liver, kidney, and spleen tissues from the normal, dysbiosis, and treatment groups to assess the impact of dysbiosis and the restorative effect of treatment. In the colon, the normal group exhibited intact mucosal architecture with well-preserved villi, normal crypt structures, and abundant goblet cells. The dysbiosis group showed multifocal moderate lymphoid hyperplasia, moderate degeneration of crypts and villi, infiltration of inflammatory cells, and shortening of villi, indicating inflammatory and degenerative alterations in the colonic mucosa. In contrast, the treatment group displayed normal villous and crypt structures, with preserved goblet cells and no evidence of inflammation or degeneration, comparable to the normal control. In the liver, all three groups showed normal hepatic histology with well-preserved hepatocytes, intact sinusoids, and normal vascular structures, without any inflammatory or degenerative changes. In the kidney, the normal group exhibited intact renal architecture with normal glomeruli and tubules. The dysbiosis group demonstrated multifocal moderate tubular degeneration with loss of brush borders and tubular epithelial cells, moderate glomerular degeneration, and mild interstitial lymphocytic infiltration. The treatment group, however, showed only minimal to mild tubular epithelial loss with preserved glomeruli and absence of interstitial inflammation, suggesting restoration of normal renal morphology. In the spleen, the normal group displayed normal white and red pulp organization. The dysbiosis group showed mild lymphoid follicular hyperplasia without changes in germinal centre activity or congestion, reflecting mild immune activation. The treatment group exhibited normal splenic architecture comparable to the control group, indicating normalization of lymphoid tissue following treatment. The proposed nutracutical formula (F3B) showed promising potential for managing gut dysbiosis. Overall, dysbiosis induced moderate inflammatory and degenerative changes in the colon and kidneys, with mild immune activation in the spleen. Treatment effectively ameliorated these alterations, restoring near-normal histological featuresacross the examined organs (Figure 13 and Supplementary Table S4).

## 4 Biostatistics

All data are presented as mean ± SEM. Data normality was assessed using the Shapiro–Wilk test. For normally distributed data, statistical significance among groups was determined using one-way ANOVA followed by Tukey's multiple comparison test. For non-normally distributed data, the Kruskal–Wallis test followed by Dunn's multiple comparison test was applied. A *P-value* < 0.05 was considered statistically significant.

## 5 Discussion

The widely recognized proverb “*prevention is better than cure*,” emphasizes the significance of preventive measures in addressing gut dysbiosis, which is effective in reducing the risk of colorectal cancer (CRC). These measures play a crucial role in preventing the formation of aberrant crypt foci (ACF), the development of adenomas, and their progression to adenocarcinomas. They also contribute to delaying the onset of the disease, reversing the early stages of carcinogenesis, and reducing the likelihood of CRC recurrence. Our key findings indicate that modulating microbial diversity in the gut microbiome through nutritional interventions, particularly a prebiotic-probiotic-based formulation, effectively restores gut dysbiosis to symbiosis. The plant derived extracts included parsley, *apigenin*; apple, quercetin; *Silybum marianum*, silymarin; grape seed, *anthocyanidins*; *Allium sativum*, allicin; green tea, EGCG; and pomegranate, ellagic acid. These phytoextracts were selected based on evidence reported in peer-reviewed scientific publications, The plant-derived phytoconstituents incorporated in the present formulation were selected based on their complementary mechanisms against colorectal cancer (CRC) and gut dysbiosis. Apigenin (parsley) exerts antiproliferative activity by suppressing the PI3K/AKT/mTOR and NF-κB pathways, inducing G2/M cell-cycle arrest, and promoting mitochondrial apoptosis through an increased BAX/BCL2 ratio and caspase activation (25). Quercetin (apple) restores intestinal microbial balance by suppressing pathogenic *Escherichia coli*, enhancing *Lactobacillus* abundance, improving epithelial barrier integrity, and inhibiting Wnt/β-catenin and STAT3 signaling (26). Silymarin (*Silybum marianum*) exhibits potent antioxidant and hepatoprotective effects by activating the Nrf2/HO-1 pathway while attenuating NF-κB-mediated inflammation and oxidative stress (27). Grape seed anthocyanidins reduce reactive oxygen species, inhibit angiogenesis and epithelial–mesenchymal transition through modulation of VEGF, MAPK, and PI3K/AKT signaling, thereby limiting tumor invasion and metastasis (28). Allicin (*Allium sativum*) induces ROS-mediated mitochondrial apoptosis, activates caspase-3/-9, and suppresses inflammatory mediators, including COX-2 and NF-κB (29). Epigallocatechin-3-gallate (EGCG) from green tea inhibits EGFR, MAPK/ERK, and Wnt/β-catenin signaling while promoting apoptosis and regulating gut microbial composition (30). Ellagic acid (pomegranate) is metabolized by gut microbiota into urolithins, which strengthen intestinal barrier function, reduce IL-6- and TNF-α-mediated inflammation, promote short-chain fatty acid-producing bacteria, and induce apoptosis through p53-dependent pathways (31). Collectively, these phytoconstituents act synergistically to restore gut eubiosis, suppress chronic inflammation, enhance immune homeostasis, and inhibit colorectal carcinogenesis, which is consistent with the superior biological activity observed for the optimized formulation.

The supporting literature on their anticancer properties is summarized in Supplementary Tables S1a, b. These compounds are increasingly recognized as a modulator of host-microbiota interactions due to their prebiotic, antioxidant, anti-inflammatory properties. These polyphenols and flavonoids are poorly absorbed in the upper gut, allowing them to reach the colon where they serve as substrates for microbial metabolize, selectively promoting beneficial taxa and inhibiting pathogens, thus shaping microbial community composition and function. These plant metabolites also enhance intestinal barrier integrity and attenuate inflammatory signaling via modulating of tight junction proteins and cytokine profiles, which is fundamental in maintaining gut homeostasis and preventing dysbiosis associated pathologies. Whereas, Quercetin has been shown to reduce pathogenic *E. coli* while increasing *Lactobacillus populations* and reinforcing epithelial barrier function which is active lead compound in present formula. Similarly, pomegranate extracts display prebiotic effects by stimulating probiotic growth and inhibiting enteric pathogens. EGCG and anthocyanidins exhibit potent antioxidant and immunomodulatory actions, further supporting mucosal health. Collectively, these compounds modulate gut ecology and host physiology through microbiota-mediated biotransformation and direct bioactivity, positioning them as promising adjuncts in probiotic and prebiotic-based immunostimulatory strategies targeting dysbiosis and associated disorders (32). The predicated molecular docking and dynamic simulation of the among phyto lead compound results revealed that that H-bonds play a significant role in ligand binding. Consideration of hydrogen-bonding properties in drug design is important because of their strong influence on drug specificity, metabolization and adsorption. Hydrogen bonds between a protein and a ligand can be further broken down into four subtypes backbone acceptor; backbone donor; side-chain acceptor; side-chain donor. The current geometric criteria for protein-ligand H-bond is: distance of 2.5 Å between the donor and acceptor atoms (D—H···A); a donor angle of 120° between the donor-hydrogen-acceptor atoms (D—H···A); and an acceptor angle of 90° between the hydrogen-acceptor-bonded atom atoms (H···A—X Next). Notably, EGCG showed the lowest predicted bioavailability score (0.17), indicating substantial limitations in systemic exposure following oral administration. These findings highlight that optimal oral bioavailability depends on the interplay of multiple physicochemical parameters rather than any single property. Overall, the ADMET predictions provide valuable insights into the pharmacokinetic behavior of these phytochemicals and support the development of advanced formulation approaches, such as lipid-based carriers, phytosomes, and polymeric nanoparticles, to overcome bioavailability limitations and enhance their therapeutic efficacy. The probiotic strains such as *Lactobacillus plantarum, Lactobacillus rhamnosus, Lactobacillus casei*, and *Bifidobacterium lactis* were included in the formulation to restore the healthy bacterial diversity in the gut microbiome. These strains have been reported to help manage gut dysbiosis and contribute to the prevention of colon cancer (33–37). Finally, we developed an efficacious, safe, and cost-effective immunomodulatory formulation to prevent CRC, reduce recurrence, and manage gut dysbiosis-associated CRC. Probiotic-derived metabolites have been shown to effectively inhibit colon cancer cell growth and suppress cell proliferation, and similar results were observed in our present study using CT-26 and HT-29 cell lines. Other research groups have demonstrated that *L. plantarum* metabolites enhance the effects of butyrate and regulate SMCT1 expression in 5-FU-resistant CRC cells (38). “Similarly, secondary metabolites of *L. rhamnosus, L. casei*, and *Bifidobacterium lactis* inhibit HT-29 colon cancer cell growth in a dose-dependent manner and subsequently increase the expression of pro-apoptotic genes, including caspase-3, caspase-9, and BAX (35). In the present study, the prebiotic-probiotic-derived formulation also inhibited the growth of CT 26 and HT-29 colon cancer cells in a dose-dependent manner (Figures 4, 5). Accumulating evidence over the past decade has elucidated the role of several enteropathogenic bacteria, including *Fusobacterium nucleatum, Bacteroides fragilis, and Escherichia coli*, in the development and progression of CRC. In clinical settings, it has been demonstrated that CRC patients harbor a higher abundance of mucosa-associated, *cyclomodulin-positive, E. coli* belonging to the B2 and D phylogroups, which exhibited strong biofilm-forming ability, compared to healthy controls (*P* < 0.05) (39). Our results revealed near-complete eradication of *E. coli* growth following treatment with secondary metabolites of the individual probiotic strains (Figure 8; Supplementary Figure S9).

Importantly, no cytotoxicity was observed in normal human embryonic kidney cells (HEK-293), indicating that the proposed formulation is safe for use in patients with colon cancer (Figures 4E–G). The scientific rationale behind this observation is that cancer cells exhibit chronically elevated reactive oxygen species (ROS) levels and altered metabolic profiles, including enhanced glycolysis, which increases their energy demands to sustain survival and proliferation (the Warburg effect). In contrast, plant-derived polyphenols and probiotic metabolites, such as butyrate, flavonoids, bacteriocins, and short-chain fatty acids (SCFAs), increase ROS levels in cancer cells beyond their oxidative stress threshold, triggering apoptosis (40). Normal cells, such as HEK-293 cells, maintain low basal ROS levels and have strong antioxidant systems, including SOD, catalase, and glutathione. Therefore, these compounds do not cause harmful oxidative stress in normal cells and may even improve the antioxidant enzyme activity (41). Furthermore, the majority of plant polyphenols and probiotic metabolites influence their effects by modulating redox processes, signaling anti-inflammatory pathways, and promoting cellular balance, all while maintaining the integrity of normal cell membranes and mitochondrial function at concentrations relevant to physiological conditions (42). Therefore, formulation 3A selectively kills cancer cells while protecting or supporting normal cell survival. Additionally, *in-silico* docking analysis showed that apigenin and quercetin had favorable binding affinities for interleukin-6 (IL6) and interleukin-10 (IL10), with docking scores indicating stable interactions. Modulation of these cytokines can suppress IL-6 mediated pro-tumor inflammation while enhancing IL-10 associated immune regulation. These active phytochemicals are the key components of our current formulation. Collectively, these findings suggest that our formulation may modulate cancer progression, reduce pro-metastatic signaling, and support immune homeostasis. The included phytocompounds could inhibit tumor cell proliferation and enhance the anti-cancer immune response. The subsequent sections investigated the anti-proliferative and immunomodulatory properties of this formulation, including its constituent compounds, using the CT26 colon cancer cell line and B and T lymphocytes derived from BALB/c mouse spleens, respectively. Furthermore, western blot analysis revealed that formulations 3A-D induced apoptosis in CT-26 cells via MAPK signaling and the mitochondrial intrinsic pathway, as confirmed by the increased BAX/BCL2 ratio. Although IL-6 is classically regarded as a pro-inflammatory cytokine and contributes to CRC progression through STAT3 activation, it also participates in immune activation, tissue repair and host defense depending on the biological context. Therefore, the observed increase in IL-6 should not be interpreted as direct evidence of an anti-inflammatory effect. Rather, the simultaneous upregulation of IL-10, together with enhanced B-cell proliferation, increased IgA/IgM production, and restoration of gut microbial diversity, indicates that the formulation exerts an overall immunomodulatory effect. Additional studies evaluating STAT3 phosphorylation, TNF-α, IL-1β, IL-17 or NF-κB signaling would be required to establish whether the formulation possesses direct anti-inflammatory activity (Figure 7). However, more investigation needed to check the expression level of BRAF on others formulation (F3A-F3C). Moreover, FSC-based flow cytometry analysis showed that formulation 3 B (a combination of Q3 and Q2) induced the highest level of apoptosis, with ~57 % total apoptotic cells, comprising ~40% late apoptosis and ~17% early apoptosis. Interestingly, plant nutrition predominantly exhibited immunostimulatory activity, whereas probiotic secondary metabolites (SMs) showed immunoinhibitory effects in splenocyte proliferation assays stimulated by ConA and LPS for T and B lymphocytes, respectively (MTT-based assay) (43–45). Overall, the formulation demonstrated immunostimulatory effects (Figure 9; Supplementary Figure S10), and exopolysaccharides produced by *Lactobacillus rhamnosus* KL37 inhibited T-lymphocyte proliferation and reduced IFN-γ expression (45, 46).

Furthermore, our *in-vivo* investigation in BALB/c mice demonstrated that antibiotic treatment (ciprofloxacin and metronidazole) induced gut dysbiosis, accompanied by chronic gut inflammation and increased gut permeability, creating a pro-tumorigenic microenvironment characterized by “inflammaging.” Treatment with the formulation improved the microbial diversity and increased the production of SCFA, particularly butyrate, which supports epithelial health (47). Additionally, probiotics strains such as *L. plantarum, L. rhamnosus, L. casei*, and *B. lactis* can transiently colonize the gut, suppress pathogenic bacteria by producing lactic acid, SCFAs, bacteriocins and EPS and ultimately stimulate mucosal immunity. However, intervention with formulation 3A successfully reversed gut dysbiosis into symbiosis (Figures 10A–I). Moreover, the restoration of bacterial diversity was confirmed by 16s rRNA metagenomic sequencing. Several beneficial bacterial genera, including *Lactobacillus, Bifidobacterium*, and *Streptococcus* were restored. Histopathological analysis was performed on the major organs, such as the liver, kidney, spleen, and colon, which were collected for microscopic examination. The findings revealed no tissue toxicity in normal control mice, mild toxicity in the treatment group, and severe inflammation and crypt distortion in the dysbiotic group. A limitation of this formulation is its susceptibility to microbial contamination during long-term storage at room temperature. Therefore, further investigation is required to evaluate and improve stability under such conditions. Overall, the developed probiotic and prebiotic-based immunostimulatory formulation is novel and represents an optimized combination of plant nutrition and probiotics. This formulation appears to be safe and effective for managing gut dysbiosis and associated chronic inflammation, colorectal cancer, and IBD.

## 6 Conclusion

In summary, the findings suggest that the proposed formulation plays a significant preventive role in mitigating gut dysbiosis and manages gut health. The formulation effectively restored gut microbial diversity, thereby promoting gut homeostasis and enhancing immune function through the activation of T- and B-lymphocyte-mediated responses. Overall, formulations F3A–F3D exhibited varying degrees of anticancer, antimicrobial, and immunostimulatory activities. Among them, formulation F3B demonstrated the most promising performance in both *in-vitro* and *in-vivo* studies, which may be attributed to the synergistic effects of its complete phytoconstituent composition, as described in the formulation Table 1.

## 7 Limitations

This study investigated the modulation of gut dysbiosis toward eubiosis in an *in-vivo* animal model using healthy mice rather than tumor-bearing mice. While the findings provide valuable insights into microbiota modulation, evaluating this preventive and/or therapeutics strategy in tumor-bearing animal models would offer a more clinically relevant understanding of the interactions between the gut microbiome, tumor progression, and therapeutic response. Furthermore, the immunological assessment was limited. A more comprehensive evaluation of the host immune response could be achieved by incorporating immune cell profiling using established immunological markers. Future studies should include the characterization of major immune cell populations, including T lymphocytes (CD4 and CD8), B lymphocytes (CD20), macrophages (CD68), and dendritic cells (CD11c), through techniques such as flow cytometry or immunohistochemistry. These analyses would provide deeper mechanistic insights into the immunomodulatory effects of the proposed polyherbal and probiotics-based formulations. Other hand, a deep investigation of molecular docking would be significantly help for understating the protein interaction toward above phytoconstituent. The present study performed docking study with limited PDB IDs.

## Data Availability

The data presented in the study are deposited in the NCBI repository, accession number PRJNA1507241 (ULR: https://www.ncbi.nlm.nih.gov/sra/PRJNA1507241).
