# Supplementary material for: A polyherbal and probiotic-based immunostimulatory formulation for managing gut dysbiosis in colorectal cancer
Source: Front Nutr. 2026 Sep 9;13:1854998. doi: 10.3389/fnut.2026.1854998 (PMC13599618; doi:10.3389/fnut.2026.1854998)
Supplement: Supplementary file 1 [file Data_Sheet_1.docx]

**A Polyherbal and Probiotic-Based Immunostimulatory Formulation for Managing Gut Dysbiosis in Colorectal Cancer**

## Abhiram Kumar^1,2,3*^, Chhavi Dhiman^1^, Vijaykumar Dayaram Nimbarte^2^, Priyanka Erukulla^1^ Madhu Rani Bharati^1^, Raktim Chattopadhyay^2^, Anubhab Mukherjee^3^, Akash Chaurasiya^2^, Kumar Pranav Narayan^1^*

*^1^ Laboratory of Molecular Medicine, Department of Biological Sciences, Birla Institute of Technology and Science Pilani, Hyderabad Campus, India*

*^2^ Department of Pharmacy, Birla Institute of Technology and Science Pilani, Hyderabad Campus, India*

***^3^*** *Esperer Institute of Oncology and Metabolic Sciences (EIOMS), Mumbai, India*

***SUPPLEMENTARY MATERIALS***

**Preparation of formulation sample for treatment:**

As mentioned above, we prepared different Formulations, F3A, F3B, F3C, and F3D). We accurately weighed 50 mg powder, transferred it into a 15 ml centrifuge tube, added 5 ml of phosphate buffer (PBS), vortexed for 5 min, and then made up the volume to 10 ml with PBS. The resulting tube was sonicated for 10 min with continuous shaking while maintaining a temperature of 30 ± 2 °C. Thereafter, we filtered the resulting solution through 0.22 μM PVDF sterile filter under a biosafety level II B cabinet and finally diluted it to four different concentrations i.e., 0.25 %, 0.125 %, 0.063 %, and 0.0312 %). with (RPMI) culture medium. All the experiments were performed under aseptic conditions.

**Animal ethical clearance**

Ethical approval for the study was obtained from the Animal Institute Ethical Committee (AIEC), with registration no. **BITS-HYD/IACE/2022-20** Birla Institute of Technology and Sciences (Bits-Pilani), Hyderabad Campus. Animals were taken care of by a 12-hour night and day cycle. Sterile food and water were provided by the Bits animal house facility. However, we declared that the collection of spleen from mice as per guidelines and the presence of an expert pharmacologist. We also declare that the experimental procedure and animal were taken care of as per animal research: reporting of in vivo experiments (ARRIVE) guidelines.

**Protocols for collection of spleen from BAL/C mice**

The spleen was collected from BAL/C mice in a normal control group by other experiments. Initially, the animal was treated with exposure of 1.5 % to 3 % of isoflurane in 100 % oxygen for Anesthetized. Further, sacrifice the animals under biosafety cabinet level 2 using sterile surgical accessories, followed by removing the spleen and transferring it into a 15 mL sterile tube containing cold PBS buffer under an ice bath.

**Powder morphology by Field Emission Scanning Electron Microscopy (FE-SEM):**

~1cm^2^ carbon tape was cut and fixed to the previously cleaned sample holder. Approx ~ 5 mg of powder formulation (3A to 3D) was weighed in batter paper and fixed with the sample holder with a slight touch. Next, the resulting sample holder was placed inside the ions removal chamber of the instrument (LEICA EM ACE200) for 20 minutes. Once the process was over, the resulting sample holder was placed into EF-SEM (Oxford Instruments) and processed the sample individually for size and morphology. The images were recorded with high resolution in different scales such as 20 µm and 50 µm.

**Lead compound identification by Liquid Chromatography Mass Spectrometry (LCMS) :**

The biologically active compound was identified from the plant extract using Shimadzu LCMS/MS (model no. SIL-20AC, detector LCMS-8040).(1) The mass spectrophotometer is equipped with an ionization chamber, ESI, and APCI  mode, nebulizer gas flow of 3 mL/minute, interface temperature of 250°C, heat block temperature of 400°C, drying gas of 10 L/minutes. Further, 5 µL of samples was introduced in a mass detector using an LC injector with mobile phase 0.1 % formic acid and LCMS grade methanol (60:40) flow rate of 0.6 mL/min. The mass spectra were recorded in ESI mode with positive and negative (+/-) scanning starting at m/z 50 to 800 range with 5000 µ/second. The recorded m/z and absolute intensity were confirmed with ChemDraw professional software (version 15.1).

**Bacterial culture conditions and preparation of secondary metabolite extracts:**

100 mg of powdered sample was inoculated in 5 mL of MRS broth and incubated overnight at 37℃ without agitation. After a significant level of turbidity was achieved, 1 mL of this suspension was inoculated in 100 mL of fresh MRS medium and incubated under similar conditions until the OD reached 0.6 (10^6^ CFU/mL). This bacterial suspension was used to prepare the secondary metabolite extracts. The cell count was determined using the CFU/mL technique. For the extraction, 100 mL of fresh MRS broth was inoculated with 5 % bacterial culture and incubated for 18-20 hours (until the culture reached its late-log or stationary phase) and centrifuged at 5000g for 20 min. The supernatant was collected and filtered through a 0.22 µm PVDF membrane. The resultant crude secondary metabolite extract was analyzed using the filter disc method.

**Table: S1a:** Active ingredient phyto-extract chosen in final formula based on proven therapeutic efficacy, S1b**.** Nutrients present in per/100 g of plant extract

| *Compound Name* | | | *Cell line* | | *IC50* | | *Time in hr.* | *Reference* | |  |  |
| --- | --- | --- | --- | --- | --- | --- | --- | --- | --- | --- | --- |
|  | | |  | |  | |  |  | |  |  |
| Apigenin | | | HT29  HL60 | | 2.82 µM  30 µM | | 48 | (2, 3) | |  |  |
| Quercetin (Apple extract) | | | CT26  HT116 | | 32 µM  39 µM | | 24 | (4) | |  |  |
| Silymarin (Silybum Marianum extract) | | | CT26 | | 50 µM | | 24 | (5) | |  |  |
| Anthocyanidins (Grape seed extract) | | | HCT-116 | | 2.4 µM | | 24 | (6) | |  |  |
| Allicin (Allium Sativum extract) | | | HT-29 | | 10–25 µM | | 24 | (7) | |  |  |
| EGCG (Green Tea extract) | | | HCT-116 | | 5 µM | | 24 | (8) | |  |  |
| Ellagic acid (Pomegranate extract) | | | Caco-2 | | 24.67 uM | | 48 | (9) | | |  |
| S2b. Nutrients present in per/100 g of plant extract | | | | | | | | | | | |
| Lead component | | *Allium Sativum*  *Extract* | *Malus domestica*  *(Apple extract)* | *Silybum marianum*  *Extract* | | [*Petroselinum crisped*](https://www.google.com/search?q=Petroselinum+crispum+extract&oq=synomanes+Parsley+Extract&gs_lcrp=EgZjaHJvbWUyBggAEEUYOdIBCTQ2NTBqMGoxNagCCLACAQ&sourceid=chrome&ie=UTF-8&mstk=AUtExfDRKBdjbJVwhZ5I8-8zcNWn9ckKDjEb7lOnWUrv60WSoLnYAQHf2H-UeUt-Yo3eSohXQoEoTqdEkSOf_sBxrQMEbfooBm3b632O6V7WIkwlj2bnU0wZC6OdLFcpeXSKp1v3s7R2174zO_9Y7Fx4T3oMDfG7i0lefGdQHXHb2vPC3TOim73BiB2qXR9UlZPS59URKbvBO4l_-8Wq4aD8emzBsSKlJAJo84TQiNKW5eOXUdNi0T32_S4XRctXbub6gxTWn9adsjlDJPcIzGpxP-L_&csui=3&ved=2ahUKEwjblOGH1fGSAxXDUGcHHQc7BmwQgK4QegQIARAB)  *(Parsley Extract)* | *Vitis vinifera*  *(Grape seed extract)* | *Camellia sinensis*  *(Green Tea extract)* | *Punica granatum*  *(Pomegranate extract)* | | |
| Water/Moisture | | 63.1 g | 83.6 g | 8.32 | | 87.71 | 3.23 g | *--* | 77.9 g | | |
| Energy | | 143 kcal | 65 kcal | 480 kcal | | 36 kcal | 384 kcal | 340 kcal | 83 kcal | | |
| Nitrogen | | 1.06 g | 0.02 g | -- | | -- | -- | -- | -- | | |
| Protein | | 6.62 g | 0.15 g | 22.17 | | 2.97 | 7.06 | 22.5 g | 1.67 g | | |
| Total lipid (Fat) | | 0.38 g | 0.16 g | 26.7 | | 0.79 | 2.86 | 0.2 g | 1.17 g | | |
| Ash | | 1.71 g | 0.43 g | 2.83 | | 2.2 | 4.33 | - | 0.53 g | | |
| Carbohydrates | | 28.2 g | 15.6 g | 67.21 g | | 6.33 | 82.6 | 60.4 g | 18.7 g | | |
| Fiber | | 2.7 g | 2.1 g | 7.17 | | -- | -- | 3.5 g | 4 g | | |
| Selenium | | 9.8 µg | -- g | -- | | 0.1µg | -- | -- | 0.5 mg | | |
| Calcium | | -- | 6 mg | -- | | 138 mg | 41.76 mg | -- | 10 mg | | |
| Iron | | -- | 0.02 mg | -- | | 6.2 | 4.13 mg | -- | 0.3 mg | | |
| Zinc | | -- | 0.02 mg | -- | | 1.07 mg | 1.66 mg | -- | 0.35 mg | | |
| Copper | | -- | 0.033 mg | -- | | 0.149 mg | 0.63 mg | -- | 0.158 mg | | |
| Manganese | | -- | 0.033 mg | -- | | 0.16 mg |  | -- | 0.119 mg | | |
| Magnesium | | -- | 4.7 mg | -- | | 50 mg | 67.90 mg | -- | 12 mg | | |
| Phosphorus | | -- | 10 mg | -- | | 58 mg | -- | -- | 36 mg | | |
| Potassium | | -- | 104 mg | -- | | 554 mg | -- | -- | 236 mg | | |
| Sodium | | -- | 1 mg | -- | | 56 mg | 0.83 mg | 0.05 g | 3 mg | | |
| Vitamin B-6 | | -- | 0.035 mg | -- | | 0.09 mg | -- | -- | 0.075 mg | | |
| Vitamins C | | 10 mg | -- | -- | | 133 mg | -- | 111 mg | -- | | |
| Thiamin | | -- | 0.006 g | -- | | 0.086 mg | -- | -- | 0.067 mg | | |
| Riboflavin | | -- | 0.068 g | -- | | 0.098 mg | -- | -- | 0.053 mg | | |
| Glucose | Total Sugar (g) | -- | 3.04 g | -- | | -- | -- | 8 g | 13.7 g | | |
| Fructose |  | -- | 8.59 g | -- | | -- | -- |  |  |  |  |
| Lactose |  | -- | 0.15 g | -- | | -- | -- |  |  |  |  |
| Sucrose |  | -- | 1.7 g | -- | | -- | -- |  |  |  |  |
| Maltose |  | -- | 0.15 g | -- | | -- | -- |  |  |  |  |
| Reference | | (10) | (11) | (12) | | (13) | (14) | (15) | (16) | | |

A

B

C

D

E

F

G

**Figure S1 | LCMS/MS spectra of biological lead identified from various plant origin samples:** such as (A)Apigenin from parsley extract, (B) Quercetin from apple extract, (C) Silymarin from Silybum Marianum extract, (D) Anthocyanidins from grape seed extract, (E) Allicin from Allium Sativum extract, (F) EGCG from green tea extract, and (G) Ellagic acid from pomegranate extract.

| *3D &2D view of Apigenin* | | *3D &2D view of Quercetin* | |
| --- | --- | --- | --- |
|  |  |  |  |
| *3D &2D view of Silymarin* | | ***3D &2D*** ***view of Anthocyanidin*** | |
|  |  |  |  |
| *3D &2D view of Allicin* | | ***3D &2D*** ***view of EGCG*** | |
|  |  |  |  |
| *3D &2D view of Ellagic acid* | | | |
|  | |  | |

**Figure S2 |** **Molecular Docking Analysis of Selected Phytocompounds with the BCL2 Anti-Apoptotic Protein**: The figure illustrates the predicted binding modes for seven phytochemical compounds docked into the active site of the BCL2 protein (PDB ID: O2F). For each compound, the left panel presents the 3D interaction within the BCL2 binding pocket, and the right panel shows the corresponding 2D ligand-residue interaction map. Specific interactions, including hydrogen bonds and hydrophobic contacts, are detailed in the 2D maps. The calculated molecular docking scores (binding energy in kcal/mol) for the tested compounds against BCL2 are as follows: Apigenin (-5.204), Quercetin (-5.640), Silymarin (-4.681), Anthocyanidins (-5.817), Allicin (-3.297), EGCG (-4.403), Ellagic acid (-4.403), and the results indicate that Anthocyanidins possess the highest predicted binding affinity for the BCL2 target.

| *3D &2D view of Apigenin* | | *3D &2D view of Quercetin* | |
| --- | --- | --- | --- |
|  |  |  |  |
| *3D &2D view of Silymarin* | | *3D &2D view of Anthocyanidin* | |
|  |  |  |  |
| *3D &2D view of Allicin* | | *3D &2D view of EGCG* | |
|  |  |  |  |
| *3D &2D view of Ellagic acid* | | | |
|  | |  | |

**Figure S3 | Molecular Docking Analysis of Selected Phytocompounds with the BAX Apoptotic Protein**: The figure illustrates the predicted binding modes for seven phytochemical compounds docked into the active site of the BAX protein. For each compound, the left panel presents the 3D interaction within the BAX binding pocket, and the right panel shows the corresponding 2D ligand-residue interaction map. Specific interactions, including hydrogen bonds and hydrophobic contacts, are detailed in the 2D maps. The calculated molecular docking scores (binding energy in kcal/mol) for the tested compounds against BAX are as follows: Apigenin (-4.540), Quercetin (-4.131), Silymarin (-4.540), Anthocyanidins (-5.844), Allicin (-1.291), EGCG (-3.877), and Ellagic acid (-4.114).The results indicate that Anthocyanidins possess the highest predicted binding affinity (lowest binding energy) for the BAX target.

| *3D &2D view of Apigenin* | | *3D &2D view of Quercetin* | |
| --- | --- | --- | --- |
|  |  |  |  |
| *3D &2D view of Silymarin* | | ***3D &2D view of Anthocyanidin*** | |
|  |  |  |  |
| *3D &2D view of Allicin* | | ***3D &2D*** ***view of EGCG*** | |
|  |  |  |  |
| *3D &2D view of Ellagic acid* | | | |
|  | |  | |

**Figure S4 | Molecular Docking Analysis of Selected Phytocompounds with the Interleukin-6 (IL6) Protein**: The figure illustrates the predicted binding modes for seven phytochemical compounds docked into the active site of the IL6 protein. The left panel shows the 3D interaction, where the compound is positioned within the electrostatic surface of the protein's binding pocket, indicating shape complementarity and residue proximity. The right panel shows the 2D ligand-residue interaction map, detailing specific interactions such as hydrogen bonds (represented by dashed lines) and hydrophobic contacts. The calculated molecular docking scores (binding energy in kcal/mol) for the tested compounds against IL6 are as follows: Apigenin (-6.577), Quercetin (-6.487), Silymarin (-4.889), Anthocyanidins (-5.343), Allicin (-1.720), EGCG (-3.993), and Ellagic acid (-3.993).The results indicate that Apigenin possesses the highest predicted binding affinity (lowest binding energy) for the IL6 target.

| *3D &2D view of Apigenin* | | *3D &2D view of Quercetin* | |
| --- | --- | --- | --- |
|  |  |  |  |
| *3D &2D view of Silymarin* | | ***3D &2D view of Anthocyanidin*** | |
|  |  |  |  |
| *3D &2D view of Allicin* | | ***3D &2D view of EGCG*** | |
|  |  |  |  |
| *3D &2D view of Ellagic acid* | | | |
|  | |  | |

**Figure S5 | Molecular Docking Analysis of Selected Phytocompounds with the Interleukin-10 (IL10) Protein**: The figure illustrates the predicted binding modes for seven phytochemical compounds docked into the active site of the IL10 protein. The left panel shows the 3D interaction, where the compound is positioned within the electrostatic surface of the protein's binding pocket, demonstrating shape complementarity and binding site topography. The right panel shows the corresponding 2D ligand-residue interaction map, detailing non-covalent contacts such as hydrogen bonds (dashed lines with arrows) and crucial stabilizing interactions like $\pi-\pi$ stacking. The calculated molecular docking scores (binding energy in kcal/mol) for the tested compounds against IL10 are as follows: Apigenin (-4.182), Quercetin (-5.915), Silymarin (-4.282), Anthocyanidins (-4.282), Allicin (-2.082), EGCG (-2.156), and Ellagic acid (-2.983). The results indicate that **Quercetin** possesses the highest predicted binding affinity (lowest binding energy) for the IL10 target.

| *3D &2D view of Apigenin* | | *3D &2D view of Quercetin* | |
| --- | --- | --- | --- |
|  |  |  |  |
| *3D &2D view of Silymarin* | | ***3D &2D view of Anthocyanidin*** | |
|  |  |  |  |
| *3D &2D view of Allicin* | | ***3D &2D*** ***view of EGCG*** | |
|  |  |  |  |
| *3D &2D view of Ellagic acid* | | | |
|  | |  | |

**Figure S6 | Molecular Docking Analysis of Selected Phytocompounds with the B-RAF Kinase Protein**.
The figure illustrates the predicted binding modes for seven phytochemical compounds docked into the active site of the B-RAF protein. For each compound, the left panel presents the 3D interaction within the B-RAF binding pocket, and the right panel shows the corresponding 2D ligand-residue interaction map. Specific non-covalent interactions, including hydrogen bonds and hydrophobic contacts, are detailed in the 2D maps. The newly calculated molecular docking scores (binding energy in kcal/mol) for the tested compounds against B-RAF are as follows: Apigenin (-8.588), Quercetin (-9.056), Silymarin (-9.132), Anthocyanidins (-8.866), Allicin (-3.780), EGCG (-7.589), and Ellagic acid (-9.025). The results indicate that Silymarin possesses the highest predicted binding affinity (lowest binding energy) for the B-RAF target in this analysis.

| *3D &2D Apigenin* | | *3D &2D Quercetin* | |
| --- | --- | --- | --- |
|  |  |  |  |
| *3D &2D Silymarin* | | ***3D &2D Anthocyanidin*** | |
|  |  |  |  |
| *3D &2D Allicin* | | ***3D &2D EGCG*** | |
|  |  |  |  |
| *3D &2D Ellagic acid* | | | |
|  | |  | |

**Figure S7** | **Detailed Molecular Docking Analysis of Selected Phytocompounds Against the K-RAS** Oncoprotein. This figure provides a visual and quantitative analysis of the predicted binding interactions between seven natural phytochemicals and the active site of the K-RAS protein. Each quadrant presents a pair of results for a single compound: the left side displays the 3D interaction showing the ligand accommodated within the electrostatic surface of the protein's binding pocket, while the right side provides the corresponding 2D map detailing key stabilizing non-covalent interactions. These 2D representations specifically highlight hydrogen bonding (dashed lines) and crucial hydrophobic/pi-pi stacking interactions necessary for binding stability. The molecular docking scores (binding energy in kcal/mol) for the tested compounds against K-RAS are: Apigenin (-5.409), Quercetin (-7.142), Silymarin (-5.814), Anthocyanidins (-5.133), Allicin (-3.850), EGCG (-2.418), and Ellagic acid (-3.674). Based on the lowest predicted binding energy, Quercetin demonstrates the strongest predicted affinity for the K-RAS target.

**Table: S2 ADMET profile of phytoconstituents**

| ADMT Predication stracture | Physicochemical/ Lipophilicity/ Water Solubility/ Pharmacokinetics and Druglikeness |
| --- | --- |
|   Quercetin | Formula: C15H10O7  Molecular weight: 302.24 g/mol  Num. heavy atoms: 22  Num. H-bond acceptors: 7  Num. H-bond donors:5  Log *P*_o/w:_ 0.79  Log *S* : -3.19  Class: Soluble  GI absorption: High  **Bioavailability Score: 0.55** |
|   Apigenin | Formula: C15H10O5  Molecular weight: 270.24 g/mol  Num. heavy atoms: 20  Num. H-bond acceptors: 5  Num. H-bond donors:3  Log *P*_o/w:_ 1.53  Log *S* : -3.23  Class: Soluble  GI absorption: High  **Bioavailability Score: 0.55** |
|   Silymarin | Formula: C25H22O10  Molecular weight: 482.44 g/mol  Num. heavy atoms: 18  Num. H-bond acceptors: 10  Num. H-bond donors:5  Log *P*_o/w:_ 1.28  Log *S*: -4.14  Class: Moderately soluble  GI absorption: Low  **Bioavailability Score: 0.55** |
|   Allicin | Formula: C6H10OS2  Molecular weight: 162.27 g/mol  Num. heavy atoms: 18  Num. H-bond acceptors: 5  Num. H-bond donors:1  Log *P*_o/w:_ 1.52  Log *S*: -1.34  Class: Very soluble  GI absorption: High  **Bioavailability Score: 0.55** |
|   **EGCG** | Formula: C22H18O11  Molecular weight: 458.37 g/mol  Num. heavy atoms: 33  Num. H-bond acceptors: 11  Num. H-bond donors:8  Log *P*_o/w:_ 0.87  Log *S*: -1.34  Class: Soluble  GI absorption: Low  **Bioavailability Score:** 0.17 |
|   Ellagic acid | Formula: C14H6O8  Molecular weight: 302.19 g/mol  Num. heavy atoms:22  Num. H-bond acceptors: 8  Num. H-bond donors:4  Log *P*_o/w:_ 1.12  Log *S*: -4.36  Class: Moderately soluble  GI absorption: Low  **Bioavailability Score: 0.55** |

A

******

*******

*******

*******

B

C

D

**Figure S8 |** MTT cytotoxicity assay on colon cancer cell lines (CT26), while formulation F3B and probiotics secondary metabolite (SM) treated on CT26 cells in various concentration in different combinations of PN & (SM) at 48h. (A) SM of *Lactobacillus plantarum* used in various combinations with PN, SM:PN 1:1 showed maximum growth inhibition of CT26 cells (B) SM of *Lactobacillus rhamnosus* used in various combinations with PN and SM, SM:PN 1:1 showed maximum growth inhibition on CT26 cells (C) SM of *Lactobacillus casei* used in various combinations with PN and SM, SM:PN 1:2 showed maximum growth inhibition on CT26 cells (D) SM of *Bifidobacterium lactis* used in various combinations with PN and SM, SM:PN 1:1 showed maximum growth inhibition on CT26 cells.

| **A**   | **B**   |
| --- | --- |
| **C**   | **D**   |
| **E**   | **F**   |
| **G**   | **H**   |

**Figure S9|** Effect of secondary metabolites along with Plant nutraceuticals (PN) combined in various ratios over the growth of *E. coli* cells studied through OD taken at 600 nm and CFU/mL method, (A) *Lactobacillus plantarum* Secondary metabolites (SM) along with PN over *E. coli* culture at a dilution of 10^-2^, (B) *Lactobacillus plantarum* SM along with PN over *E. coli* culture at a dilution of 10^-6^, (C) *Lactobacillus rhamnosus* SM along with PN over *E. coli* culture at a dilution of 10^-2^, (D) *Lactobacillus rhamnosus* SM along with PN over *E. coli* culture at a dilution of 10^-6^, (E) *Lactobacillus casei* SM along with PN over *E. coli* culture at a dilution of 10^-2^, (F) *Lactobacillus casei* SM along with PN over *E. coli* culture at a dilution of 10^-6^, (G) *Bifidobacterium lactis* SM along with PN over *E. coli* culture at a dilution of 10^-2^, (H) *Bifidobacterium lactis* SM along with PN over *E. coli* culture at a dilution of 10^-6^; A: SM alone; SM and PN in the ratios of B- 1:2; C- 1:1; D- 2:1; PN- Plant nutraceutical alone; U- Untreated. *All the produced data is statistically significant with a p value of <0.001. Note: The dilutions refer to those made to the bacterial culture and is serially diluted from the mother culture.*

**Figure S10|** Fecal pellet size comparison across normal, dysbiotic control, and treatment group mice.
visual representation and dimensional analysis of fecal pellets from the three experimental groups. The Dysbiotic control mice exhibited significantly smaller fecal pellet sizes compared to both the normal control mice and the treatment group mice. Crucially, statistical analysis revealed no significant difference in pellet size between the normal control mice and the treatment group mice, indicating that the intervention successfully restored fecal morphology.

**Figure S11| Gram staining of bacterial populations from fecal samples of healthy, dysbiotic, and treated mice:** Representative microscopic images showing Gram staining. Fecal smears from healthy mice exhibited a dense and diverse bacterial population, whereas dysbiotic mice showed markedly reduced bacterial load, indicating microbiota depletion. In treated mice, bacterial abundance was restored to near-normal levels, suggesting recovery of the intestinal microbial community.

**Table S3:** The results of apoptosis assessed by flow cytometry are summarized in the table below, with all four different quadrants.

**Table S4:** Histopathological evaluation was conducted on the H&E-stained colon, liver, kidney, and spleen tissues from the normal, dysbiosis, and treatment groups

| Parameters | Normal Control Group | Dysbiosis Group | Treatment Group |
| --- | --- | --- | --- |
| Colon |  |  |  |
| Inflammatory cells infiltrate | 0 | 2^++^ | 0 |
| Crypt architecture | 0 | 2^++^ | 0 |
| Goblet cells depletion | 0 | 1^+^ | 0 |
| Kidney |  |  |  |
| Tubular degeneration/ necrosis | 0 | 2^++^ | 1 |
| Glomerulus degeneration/ necrosis | 0 | 2^++^ | 0 |
| Interstitial Inflammation | 0 | 1^+^ | 0 |
| Spleen |  |  |  |
| Lymphoid follicles change (Hyperplasia/atrophy) | 0 | 1^+^ | 0 |

# ***Note:*** Scoring table (0-3), None – 0, Mild -1^+^ Moderate -2^++^, Severe-3^+++^. Liver: there is no nay significant difference was observed for normal control, dysbiosis control and treatment group. The liver from all three groups appeared histologically normal, with well-preserved hepatocytes, intact sinusoidal and vascular structures, and no evidence of inflammatory, steatosis, fibrosis or degenerative changes.

**Results and discussion**

1. **Bioinformatics**

BCL-2 is an anti-apoptotic protein that inhibits mitochondrial outer membrane permeabilization, thereby preventing cytochrome-c release and suppressing the intrinsic apoptotic pathways(17). While BAX is a pro-apoptotic protein that promotes mitochondrial outer membrane permeabilization, facilitating cytochrome-c release and activating the intrinsic apoptotic pathways(18). Other hand IL6 and IL10 is a pro-inflammatory cytokine that plays a key role in immunomodulation by regulating immune cells activation, differentiation, and the valance between pro ad anti-tumor immune responses(19). Following to B RAF and K RFA, is an oncogenic protein that mostly over express in colon cancer cells via MAPK/ERK signaling pathway, promoting uncontrolled cell proliferation and survival(20). Mutations in these genes enhance tumor progression by sustaining constitutive signaling and enabling resistance and enabling resistance to apoptosis. However, in present investigation, all the phytocompounds such as *Anthocyanidin, Apigenin, Quercetin, EGCG, Silymarin, Ellagic acid* and *Allicin* were docking with above mentioned proteins. Further, molecular dynamics study was performed based on their protein and ligand interaction score. The optimal score was recorded with quercetin. The following results and discussion cover all the phytobiological lead compounds in relation to their respective target proteins

1.1 **Molecular docking with BCL2—PDB ID: 2O2F)(21)**
    1.1.1 ***Apigenin (Binding energy* -5.204**)***:*** The binding interaction of apigenin with BCL2 (PDB ID: 2O2F) demonstrates a favorable engagement within the protein’s ligand-binding pocket, stabilized by a combination of hydrogen bonding and hydrophobic contacts. In the 2D interaction diagram, apigenin forms two key hydrogen bonds with ASP100, a critical residue that anchors the ligand through interactions with its hydroxyl groups. Hydrophobic interactions involving PHE101, TYR105, TYR199, ALA146, and VAL145 further stabilize the ligand by reinforcing its position within the non-polar environment of the binding groove. Additional surrounding residues such as GLN96, ALA97, ARG104, and GLY142 contribute to the overall shape and electrostatic profile of the pocket, promoting optimal ligand fit. The 3D electrostatic surface representation confirms that apigenin is deeply embedded within a pocket surrounded by both polar and hydrophobic surfaces, where negatively charged ASP100 and neutral residues like GLY142 form complementary interactions with the ligand. These findings suggest that apigenin binds stably within the BCL2 active site, primarily driven by hydrogen bonding and hydrophobic interactions **( Fig. S2).**
    1.1.2 ***Quercetin (Binding energy* -****5.640**)***:*** The binding interaction of quercetin with the anti-apoptotic protein BCL2 (PDB ID: 2O2F) is characterized by a network of hydrogen bonds and hydrophobic interactions that stabilize the ligand within the binding pocket. In the 2D interaction diagram, quercetin forms key hydrogen bonds with ASP137 and ARG143, which are crucial for anchoring the molecule through its hydroxyl groups. Additional polar contacts with GLU133, ARG136, and ARG143 further enhance ligand stabilization. Hydrophobic residues such as PHE101, PHE109, PHE150, MET112, and VAL130 contribute to van der Waals interactions, reinforcing the ligand’s positioning within the lipophilic environment of the pocket. Residues LEU134, ALA146, and GLU133 also shape the overall geometry of the binding site. The 3D electrostatic surface representation reveals quercetin deeply buried within a pocket lined with both positively and negatively charged residues, notably ASP137 and ARG143, which form directional hydrogen bonds with the ligand. These observations indicate that quercetin exhibits strong binding affinity toward BCL2 through a combination of hydrogen bonding and hydrophobic contacts, suggesting its potential as a modulator of BCL2-mediated apoptotic signaling**( Fig. S2).**.
    1.1.3 ***Silymarin (Binding energy* -4.681**)***:*** The molecular interaction analysis of silymarin with the BCL2 protein (PDB ID: 2O2F) reveals a complex and stabilized binding mode, driven by a combination of hydrogen bonding, electrostatic interactions, and hydrophobic contacts. The 2D interaction map shows that silymarin establishes key hydrogen bonds with ASP100, ASP108, ARG104, ARG143, and GLU133, mediated by its multiple hydroxyl and oxygen-containing functional groups. These interactions are crucial for anchoring silymarin within the binding groove of BCL2. Additionally, the ligand is surrounded by a hydrophobic environment formed by residues such as PHE101, TYR105, TYR199, VAL145, LEU134, ALA145, and MET112, contributing to van der Waals interactions and further stabilizing the binding conformation. Residues such as ALA97, ALA146, GLY142, and TRP141 also play supportive roles in pocket architecture and ligand orientation. The 3D electrostatic surface representation confirms that silymarin fits snugly within the binding cavity, with notable interactions involving ARG143, TYR105, and ALA97, highlighting the electrostatic and spatial complementarity between the ligand and the protein. These findings suggest that silymarin exhibits strong and stable binding to BCL2 through synergistic hydrogen bonding and hydrophobic interactions, positioning it as a promising candidate for modulating BCL2-mediated apoptotic pathways**( Fig. S2).**
    1.1.4 ***Anthocyanidin (Binding energy* -5.817**)***:*** The interaction analysis of anthocyanidin with the BCL2 protein (PDB ID: 2O2F) reveals a well-defined binding mode characterized by multiple stabilizing contacts within the ligand-binding pocket. In the 2D interaction diagram, anthocyanidin forms critical hydrogen bonds with GLU133 and ASP137, which play a key role in anchoring the ligand through polar interactions. Additionally, hydrophobic contacts with residues such as PHE101, TYR105, PHE109, and MET112 contribute to ligand stabilization within the predominantly non-polar environment of the pocket. Surrounding residues including ASP108, GLN115, LEU134, ARG136, ARG143, and ALA146 further define the pocket architecture and may aid in maintaining the ligand-bound conformation of the protein. The 3D electrostatic surface view illustrates that anthocyanidin is deeply embedded within the BCL2 binding cleft, where it interacts with a network of negatively charged residues (ASP108, GLU133, ASP137) through its polyhydroxyl groups. The presence of complementary positively charged and neutral regions enhances the overall binding complementarity, suggesting that electrostatic and hydrophobic forces collectively drive the stable interaction of anthocyanidin with BCL2**( Fig. S2).**
    1.1.5 ***Allicin (Binding energy* -3.297**): The binding interaction of allicin with BCL2 (PDB ID: 2O2F) reveals a compact and hydrophobically driven binding mode within a narrow pocket of the protein. The 3D electrostatic surface view shows allicin (rendered in green with sulfur atoms in yellow and oxygen in red) nestled within a hydrophobic cavity bordered by key residues, notably VAL130, which appears to be in close proximity and may engage in van der Waals interactions with the aliphatic backbone of allicin. The binding site is surrounded by both negatively charged (red) and positively charged (blue) electrostatic regions, but allicin primarily interacts through nonpolar contacts, indicating a dominant role for hydrophobic interactions. The sulfur-containing moiety of allicin may also allow for weak dipolar or polarizable contacts with nearby residues, although no strong hydrogen bonding is evident from the current view. These interactions suggest that allicin binds stably to BCL2 via shape complementarity and hydrophobic interactions, which may contribute to modulation of BCL2 function by occupying key regulatory sites involved in anti-apoptotic activity.
    1.1.6 ***EGCG (Binding energy* -4.403**)***:*** The binding interaction of epigallocatechin gallate (EGCG) with BCL2 (PDB ID: 2O2F) reveals a well-defined and multi-faceted engagement within the protein’s binding pocket, characterized by a dense network of hydrogen bonds and hydrophobic contacts. In the 2D interaction diagram, EGCG forms strong hydrogen bonds with GLU133, ASP137, and ARG136, primarily mediated by its multiple hydroxyl groups. These polar interactions are key contributors to ligand stabilization and orientation within the pocket. An additional hydrogen bond with ARG143 further reinforces binding, while TYR105 participates in π-π stacking and hydrophobic interactions, enhancing ligand affinity. The interaction is further stabilized by van der Waals contacts with several hydrophobic and non-polar residues including PHE101, PHE109, PHE150, VAL130, MET112, LEU134, and ALA146, which collectively contribute to a snug fit of EGCG in the hydrophobic groove. The 3D electrostatic surface view illustrates EGCG occupying a deep pocket lined by both acidic and basic residues, with prominent interactions involving GLU133, ARG143, and TYR105, emphasizing the electrostatic complementarity of the ligand. These findings suggest that EGCG achieves stable and high-affinity binding to BCL2 through a synergy of hydrogen bonding, electrostatic interactions, and hydrophobic contacts, highlighting its potential as an effective modulator of BCL2 activity**( Fig. S2).**
    1.1.7 ***Ellagic acid (Binding energy*** -**4.403**)***:***The interaction analysis of ellagic acid with the anti-apoptotic protein BCL2 (PDB ID: 2O2F) demonstrates a highly polar and stable binding mode driven primarily by hydrogen bonding and electrostatic interactions. In the 2D interaction diagram, ellagic acid forms strong hydrogen bonds with the negatively charged residue ASP137, facilitated by its hydroxyl groups located on the polyphenolic rings. Additional surrounding residues such as ARG136, GLU133, LEU134, ARG143, and MET112 contribute to shaping the electrostatic and structural environment of the binding pocket. Notably, the extended conjugated system and multiple hydroxyl functionalities of ellagic acid allow it to participate in additional potential polar contacts and pi interactions. The 3D electrostatic surface representation confirms that ellagic acid is well accommodated in a polar cavity lined by both acidic (ASP137, ASP108) and basic (ARG136, ARG143) residues, providing a strong electrostatic complementarity. These interactions suggest that ellagic acid binds in a favorable orientation within the BCL2 binding pocket, potentially modulating its anti-apoptotic activity by interfering with protein-protein interactions involved in apoptotic regulation**(Fig. S2).**
1.2 **Molecular docking with *BAX— PDB: 6EB6(22)***
    1.2.1 ***Apigenin (Binding energy -4.540):*** The interaction of apigenin with the BAX protein (PDB: 6EB6) reveals a modest yet specific binding pattern, primarily stabilized by hydrogen bonding and electrostatic interactions. In the 3D docking image (left panel), apigenin (green) is nestled within a moderately polar pocket of BAX, forming a key hydrogen bond with GLU41, a negatively charged residue critical for ligand anchoring. The 2D interaction diagram (right panel) confirms this interaction and shows apigenin positioned adjacent to several important residues, including ASP33, GLN32, ARG37, and GLY36, though direct bonding is limited to GLU41. The compound’s hydroxyl groups play a pivotal role in facilitating its binding, contributing to hydrogen bonding potential and partial stabilization within the pocket. While the binding profile is less extensive than more complex polyphenols, the specific interaction with GLU41 suggests apigenin may still influence BAX conformational dynamics and could serve as a lead scaffold for further optimization in apoptosis-targeted therapeutic development **(Fig. S3).**
    1.2.2 ***Quercetin (Binding energy -4.540 Kcal/mol):*** The binding interaction of quercetin with the BAX protein (PDB: 6EB6) reveals a strong affinity mediated through multiple hydrogen bonds and electrostatic interactions within the protein’s active cleft. The 3D binding pose (left panel) shows quercetin (green) deeply embedded within a negatively charged pocket, forming direct hydrogen bonds with key residues ASP33 and ASP68. This is further supported by the 2D interaction diagram (right panel), which highlights additional polar interactions with nearby residues such as ARG65 and LYS64, likely contributing to electrostatic stabilization. The hydroxyl groups on both phenyl rings of quercetin play a crucial role in anchoring the molecule via hydrogen bonding, especially with the carboxylate groups of ASP33 and ASP68. These molecular interactions suggest that quercetin binds favorably to BAX, potentially modulating its conformational dynamics and apoptotic function, supporting its promise as a natural compound with therapeutic relevance in apoptosis-driven pathologies such as cancer**(Fig. S3).**
    1.2.3 ***Silymarin (Binding Energy -4.540 Kcal/mol):*** The interaction of silymarin with the BAX protein (PDB: 6EB6) demonstrates a robust binding profile characterized by an extensive network of hydrogen bonds and polar contacts, suggesting a high affinity and potential functional modulation of the protein. In the 3D binding model (left panel), silymarin (in green) spans a large surface of the BAX binding cleft, interacting with several key residues including GLU41, GLU61, LYS57, and PRO43. These interactions are further detailed in the 2D interaction map (right panel), where silymarin forms hydrogen bonds through its hydroxyl and ether oxygen groups with residues such as ASP33, GLU41, GLU61, and LYS57, alongside contacts with hydrophobic and polar residues like PRO49, GLN52, and ALA42. The polyphenolic structure of silymarin facilitates its strong anchoring within the pocket, stabilized by both electrostatic and hydrophobic interactions. This binding mode suggests that silymarin may influence BAX conformational states, thereby modulating its pro-apoptotic activity and supporting its potential as a therapeutic agent targeting apoptosis pathways **(Fig. S3).**
    1.2.4 ***Anthocyanidin (Binding energy -5.844 Kcal/mol):*** The interaction of anthocyanidins with the pro-apoptotic protein BAX (PDB: 6EB6) is characterized by a strong network of hydrogen bonds and polar interactions that stabilize the ligand within the binding groove. The 3D docking visualization (right panel) shows anthocyanidins (in green) fitting snugly into a hydrophilic pocket formed by key residues such as ASP33, GLU41, and PRO43. These interactions are corroborated by the 2D interaction map (left panel), which reveals multiple hydrogen bonds between the hydroxyl groups of the anthocyanidin scaffold and residues GLU41, PRO43, and ASP33, with additional stabilizing contacts involving ALA35, GLY36, and GLN32. The presence of a positively charged oxygen (O⁺) in the anthocyanidin core may enhance electrostatic attraction with negatively charged residues like ASP33 and GLU41. These molecular interactions suggest that anthocyanidins may modulate BAX activity by stabilizing conformations associated with its active or inactive state, supporting their potential role in apoptosis regulation and cancer therapeutics **(Fig. S3).**
    1.2.5 ***Allicin ( Binding Energy = -1.291 Kcal/mol):*** The interaction of allicin with the BAX protein (PDB: 6EB6) exhibits a focused and specific binding mode, characterized by key polar interactions within a predominantly hydrophobic pocket. In the 3D docking visualization (left panel), allicin (green) binds close to ARG34, forming a hydrogen bond with its guanidinium group, as confirmed by the 2D interaction map (right panel). This key interaction is complemented by proximity to additional residues such as ARG37, MET38, and LEU122, which contribute hydrophobic stabilization. The sulfoxide group in allicin plays a central role in its binding, facilitating the hydrogen bond with ARG34, while the diallyl groups may engage in van der Waals interactions with surrounding nonpolar residues. The relatively small and flexible structure of allicin allows it to fit into the binding pocket without steric hindrance. This interaction pattern suggests that allicin may subtly influence BAX’s conformational dynamics, potentially modulating its pro-apoptotic activity, and highlights its promise as a natural compound in apoptosis-related therapeutic strategies**(Fig. S3).**
    1.2.6 ***EGCG (Binding energy -3.877 Kcal/mol):*** The interaction of Epigallocatechin gallate (EGCG) with the BAX protein (PDB: 6EB6) demonstrates a highly stable and multivalent binding profile, primarily driven by extensive hydrogen bonding and electrostatic interactions. The 3D docking image (left panel) shows EGCG (in green) occupying a prominent surface pocket flanked by key charged and polar residues such as GLU61, LYS64, and ASP68. The 2D interaction diagram (right panel) highlights a dense network of hydrogen bonds formed by EGCG’s multiple hydroxyl groups with GLU61, ASP33, ASP68, and LYS64, with additional stabilizing contacts involving ARG65 and LYS57. The gallate and catechin moieties contribute to the ligand's high polarity and flexibility, allowing it to engage multiple residues simultaneously and achieve a snug fit in the binding cleft. These interactions suggest that EGCG may effectively modulate BAX’s pro-apoptotic function by stabilizing specific conformations, supporting its potential role as a natural modulator of apoptosis in therapeutic contexts such as cancer prevention and treatment**(Fig. S3)**.
    1.2.7 ***Ellagic acid (Binding Energy -4.114 Kcal/mol):*** The interaction of ellagic acid with the BAX protein (PDB: 6EB6) displays a strong binding affinity, primarily mediated through multiple hydrogen bonds within a polar and electrostatically active binding pocket. The 3D docking visualization (left panel) shows ellagic acid (green) deeply embedded in a cavity lined by key residues such as GLU61 and LYS64, with the compound oriented to maximize polar contacts. The corresponding 2D interaction map (right panel) highlights crucial hydrogen bonding interactions between the hydroxyl groups of ellagic acid and residues GLU61, SER60, and LYS64, while also positioning it near ASP33 and GLN32, which may contribute to secondary electrostatic stabilization. The conjugated ring system of ellagic acid likely enhances π–π stacking interactions and contributes to molecular rigidity, aiding its fit within the BAX pocket. These interactions suggest that ellagic acid may influence the conformational state of BAX, potentially modulate its apoptotic activity, and underscores its potential as a natural apoptotic regulator in cancer therapy**(Fig. S3)**.
1.3 **Molecular docking with *IL6 — PDB: 1ALU*(23)**
    1.3.1 ***Apigenin:*** The molecular interaction analysis of Apigenin with Interleukin-6 (IL-6), based on the crystal structure PDB ID: 1ALU, reveals a stable and specific binding orientation within the IL-6 active site. The 2D interaction diagram highlights key hydrogen bonds formed between Apigenin’s hydroxyl groups and crucial residues including ASN63 and GLU172, indicating strong polar interactions that contribute to binding stability. Additional residues such as ASN61, LEU62, LEU64, LYS66, ARG168, and SER169 contribute to a network of van der Waals and hydrophobic contacts, further anchoring Apigenin within the binding cleft. The flavone core of Apigenin fits snugly between both polar and nonpolar regions of the binding site, suggesting its dual capacity to engage in electrostatic as well as hydrophobic interactions. These molecular contacts support Apigenin's potential as a promising natural IL-6 modulator, with implications for anti-inflammatory and cytokine-regulatory therapeutic strategies**(Fig. S4)**.
    1.3.2 ***Quercetin :*** The interaction analysis of Quercetin with Interleukin-6 (IL-6), derived from the crystal structure PDB ID: 1ALU, demonstrates a stable and well-oriented binding conformation within the cytokine's active site. The surface view shows Quercetin nestled within a pocket formed by several key residues including ARG168, GLU172, and LEU62. The 2D interaction diagram reveals extensive hydrogen bonding interactions involving the hydroxyl groups of Quercetin with polar residues such as GLU172, ARG168, SER169, and ASN61, highlighting the compound’s strong binding affinity. Additional stabilization is provided through hydrophobic interactions with residues LEU62, LEU64, LEU165, and PRO65, suggesting a snug fit within the IL-6 pocket. The molecular interactions observed underscore Quercetin’s potential as an anti-inflammatory agent, capable of modulating IL-6 activity through direct binding**(Fig. S4)**.
    1.3.3 ***Silymarin:*** The molecular docking analysis reveals that Silymarin binds within the active site of IL-6, forming a stable complex through multiple non-covalent interactions. The 3D surface representation (left panel) shows Silymarin (in green stick representation) nestled within a hydrophilic and partially charged pocket, with key interacting residues including Glu172, Lys66, Asn63, and Leu62. The 2D interaction diagram (right panel) highlights extensive hydrogen bonding and van der Waals interactions that contribute to binding stability. Notable interactions include—Hydrogen bonds between hydroxyl groups of Silymarin and residues Glu172, Lys66, and Glu93, enhancing specificity and affinity; Polar contacts with Asn61, Asn63, and Ser169, contributing to electrostatic stabilization; Hydrophobic interactions with residues Leu62, Leu64, Pro65, and Ala68, helping anchor the compound in the binding pocket; Additional involvement of Arg168 and Lys86 suggesting a possible role in ligand orientation and stabilization. These findings suggest that Silymarin has strong binding potential with IL-6, driven by a combination of hydrogen bonding, polar interactions, and hydrophobic contacts, which may contribute to its proposed anti-inflammatory or immunomodulatory effects **(Fig. S4)**.
    1.3.4 ***Anthocyanin***: The interaction of anthocyanin with Interleukin-6 (IL-6), as depicted using PDB structure 1ALU, reveals a stable binding conformation within the IL-6 binding pocket. The 3D visualization on the left shows’ anthocyanin (in green stick form) nestled within a surface-rendered cavity of IL-6, establishing multiple non-covalent interactions. Key residues involved in stabilizing the ligand include LEU62, ASN63, and GLU172, which are in close proximity and form significant contacts. In the 2D interaction map on the right, detailed interactions between anthocyanin and IL-6 residues are illustrated. Anthocyanin forms several hydrogen bonds with polar residues such as ASN61, ASN63, and GLU172, contributing to the specificity and stability of the binding. Notably, hydroxyl groups on the anthocyanin molecule are involved in direct hydrogen bonding with these residues. Additional van der Waals interactions with non-polar residues like LEU62 and LEU64 enhance hydrophobic contacts, further anchoring the ligand in the binding site. These interactions suggest that anthocyanin exhibits a strong binding affinity towards IL-6, possibly modulating its activity. This molecular binding insight supports the potential of anthocyanin as a natural modulator of IL-6, which could be valuable in developing anti-inflammatory or immune-regulating therapeutics **(Fig. S4)**.
    1.3.5 ***Allicin (IL6 (PDB: 1ALU*):** Th**e** binding interaction of allicin with Interleukin-6 (PDB ID: 1ALU) reveals a well-oriented fit within a hydrophilic pocket lined with both polar and charged amino acid residues. The 3D electrostatic surface representation shows that allicin occupies a groove surrounded by ARG168 and GLU172, which contribute to the electrostatic stabilization of the ligand. The electrophilic sulfoxide moiety of allicin forms a hydrogen bond interaction with ARG168, as evident in the 2D interaction map. Additionally, the molecule is further stabilized by close spatial contacts with SER169, LYS66, and GLU172, suggesting a combination of electrostatic and polar interactions driving the binding affinity. The surrounding nonpolar residues, including LEU62, LEU64, and LEU165, likely contribute to hydrophobic interactions with the allyl groups of allicin, enhancing the ligand’s accommodation and anchoring within the pocket. Overall, the interaction profile indicates that allicin is favourably bound through a synergistic interplay of hydrogen bonding, electrostatic complementarity, and hydrophobic contacts, supporting its potential to modulate IL-6 activity through direct molecular engagement**(Fig. S4)**.
    1.3.6 ***EGCG:*** Molecular interaction of Epigallocatechin gallate (EGCG) with interleukin-6 (IL-6, PDB ID: 1ALU), EGCG binds within a prominent surface pocket of IL-6, forming a network of stabilizing interactions. The 2D interaction diagram reveals multiple hydrogen bonds between the hydroxyl groups of EGCG and key residues, including GLU172, SER169, ARG168, LYS66, and ASN61. Additionally, hydrophobic interactions are observed with residues LEU62, LEU64, and LEU165, which may further stabilize the binding conformation. The electrostatic surface map in the 3D view illustrates that EGCG resides in a negatively charged pocket, consistent with the predominance of its polar functional groups, suggesting that hydrogen bonding and polar contacts are central to its mode of interaction. These findings support the potential of EGCG to modulate IL-6 activity through direct binding at this surface-exposed pocket **(Fig. S4)**.
    1.3.7 ***Ellagic acid:*** The binding interaction of Ellagic acid with Interleukin-6 (IL-6), based on the crystal structure from PDB ID: 1ALU, reveals a favorable binding mode within the IL-6 binding pocket. The molecular surface representation highlights the key interacting residues including ARG168, GLU172, and LEU62, which form critical contacts with the polyphenolic core of Ellagic acid. The 2D interaction map shows multiple hydrogen bonds between Ellagic acid and polar residues such as GLU172, SER169, ARG168, ASN61, and LYS66, stabilizing the ligand within the active site. These interactions, particularly the hydrogen bonds with hydroxyl groups of Ellagic acid, suggest strong affinity and potential inhibitory activity. Additionally, hydrophobic contacts with residues such as LEU62, LEU64, and LEU165 further reinforce ligand binding. This interaction profile supports Ellagic acid’s potential as a natural anti-inflammatory agent targeting IL-6**(Fig. S4)**.
1.4 **Molecular docking with IL-10—PDB ID: 2ILK(24)**
    1.4.1 ***Apigenin (Binding energy = -5.915 Kcal/mol)****:* The interaction of apigenin with Interleukin-10 (IL-10), based on the crystallographic structure PDB ID: 2ILK, demonstrates a well-defined binding orientation within the IL-10 binding cleft. The 3D visualization highlights that apigenin (in green stick form) is positioned snugly within a hydrophobic pocket, surrounded by residues such as PHE30, TYR72, and LEU94. These residues form stabilizing van der Waals interactions, anchoring the ligand within the protein structure. The 2D interaction map further illustrates key interactions, including hydrogen bonding between the oxygen of apigenin and the hydroxyl group of TYR72, suggesting a crucial role in ligand recognition and stabilization. Additional hydrophobic contacts are observed with residues such as LEU26, VAL33, VAL76, MET77, and ALA80, enhancing the non-polar interactions that stabilize the apigenin-IL-10 complex. Notably, π-π stacking with aromatic residues such as PHE30 contributes to the strong binding affinity and orientation of the flavonoid core. These findings underscore apigenin's potential to modulate IL-10 signaling through stable binding, offering a promising scaffold for the development of immunomodulatory agents targeting anti-inflammatory pathways **(Fig. S5)**.
    1.4.2 ***Quercetin (-4.108 Kcal/mol):*** The interaction of quercetin with Interleukin-10 (IL-10), as derived from the 3D structural data of PDB ID: 2ILK, highlights a strong and spatially optimized binding conformation within the cytokine’s receptor-binding pocket. In the 3D binding visualization, quercetin (green stick model) is deeply embedded within the IL-10 surface groove, forming critical polar interactions with several key amino acid residues. Specifically, quercetin establishes multiple hydrogen bonds with ASP28 and LYS34, contributing to the anchoring and stabilization of the ligand. The hydroxyl groups of quercetin play a central role in mediating these hydrogen bonds, enhancing both specificity and affinity. The 2D interaction map further reveals additional contacts with ARG27, PHE30, and SER31. ARG27 and ASP28 form a polar cluster with quercetin’s hydroxyl groups, while LYS34 engages in hydrogen bonding with the carbonyl and hydroxyl moieties of the flavonoid core. Furthermore, π-π stacking with PHE30 and van der Waals interactions with surrounding residues enhance hydrophobic stabilization. The extended conjugated ring system of quercetin facilitates favorable spatial interactions across the IL-10 surface, aligning effectively with the electrostatic and topological features of the binding site. These findings underscore quercetin's potential as a natural immunomodulator with specific affinity for IL-10, capable of influencing its anti-inflammatory signaling. Its well-defined binding mode supports its candidacy as a lead compound for therapeutic modulation of IL-10 pathways**(Fig. S5)**..
    1.4.3 ***Silymarin (-4.286 Kcal/mol)*** The interaction of silymarin with Interleukin-10 (IL-10), based on the structural analysis using PDB ID: 2ILK, reveals a robust and multi-faceted binding engagement within the IL-10 binding groove. The 3D visualization illustrates that silymarin (green stick model) is comfortably situated within a well-defined hydrophobic pocket, forming multiple key interactions with surrounding amino acid residues. Crucial hydrogen bonds are observed between silymarin and polar residues such as ARG27, ASP28, and SER31, stabilizing the ligand within the pocket. These interactions are complemented by electrostatic attractions and orientation-specific contacts that reinforce the ligand's conformation. The 2D interaction diagram further delineates these interactions, highlighting hydrogen bonding with ASP28 and ARG27, along with π-π stacking and van der Waals interactions involving PHE30. Nonpolar residues like LEU26, LEU94, LEU98, and MET77 provide a hydrophobic environment that further supports ligand accommodation through van der Waals forces. The multiple hydroxyl and methoxy substituents on the silymarin scaffold enable extensive interaction with the IL-10 binding site, enhancing binding affinity and specificity. These results suggest that silymarin forms a strong and specific complex with IL-10, potentially modulating its anti-inflammatory activity. The diverse interaction profile of silymarin underscores its potential as a natural immunomodulatory agent and a promising lead for the development of IL-10-targeted therapeutics **(Fig. S5)**.
    1.4.4 ***Anthocyanin (-4.282 Kcal/mol):*** The molecular docking analysis of anthocyanin with Interleukin-10 (IL-10), based on the structural conformation of PDB ID: 2ILK, reveals a well-anchored interaction primarily governed by polar contacts within the cytokine's binding pocket. As visualized in the surface-rendered 3D model, anthocyanin (shown in green stick format) is embedded deeply within the hydrophilic groove, forming key hydrogen bonds with two crucial residues: ASP28 and ARG27. These interactions are characterized by the donation and acceptance of hydrogen bonds between the hydroxyl groups of anthocyanin and the side chains of these polar residues. ASP28 engages in strong hydrogen bonding via its carboxylate moiety, contributing to both electrostatic stabilization and spatial orientation of the ligand, while ARG27 forms additional hydrogen bonding interactions, reinforcing the molecular affinity. This positioning of anthocyanin within the active cleft of IL-10 implies a potential to modulate the cytokine’s activity, possibly by interfering with its receptor binding or signaling interface. The predominance of polar interactions, along with the tight spatial fit observed in the binding cavity, suggests a high degree of specificity and binding affinity. Collectively, these findings support the therapeutic potential of anthocyanin as a natural modulator of IL-10’s anti-inflammatory functions, and its candidacy for further development in immunomodulatory drug discovery **(Fig. S5)**.
    1.4.5 ***Allicin (binding score: -2.082 Kcal/mol):*** The docking interaction of allicin with Interleukin-10 (IL-10), analysed using the structural model from PDB ID: 2ILK, reveals a compact and hydrophobic mode of binding, driven primarily by non-polar contacts. The 3D visualization shows allicin (green stick form) residing within a hydrophobic cleft on the IL-10 surface, engaging closely with residues such as LEU23, LEU26, LEU94, and PHE30. The 2D interaction map confirms this van der Waals interactions, suggesting that allicin is stabilized in the pocket through hydrophobic association rather than extensive hydrogen bonding. A notable polar contact is observed with ARG27, likely mediated via the oxygen atom of allicin’s sulfoxide group, which helps orient the molecule within the binding site. The overall interaction profile reflects allicin’s small, flexible structure, allowing it to adapt to the IL-10 groove while maintaining a low-energy conformation. The lack of extensive polar interactions is compensated by deep hydrophobic embedding and potential electrostatic alignment with ARG27. These findings indicate that allicin may modulate IL-10 function through conformational stabilization or mild allosteric influence, supporting its relevance as a dietary-derived immunomodulator with potential therapeutic applications**(Fig. S5)**.
    1.4.6 ***EGCG (Binding energy = -0.595 Kcal***/mol): The interaction analysis of Epigallocatechin gallate (EGCG) with Interleukin-10 (IL-10), based on docking studies using the crystal structure PDB ID: 2ILK, reveals a highly intricate and stable binding pattern within the cytokine’s active site. The 3D binding model shows EGCG (green stick form) deeply embedded in a well-defined polar pocket of IL-10, forming a dense network of hydrogen bonds with several key amino acid residues. Notably, EGCG establishes strong polar interactions with ASP25, ASP28, ARG24, ARG27, and SER31, which collectively create a robust electrostatic environment anchoring the polyphenolic scaffold. The 2D interaction diagram highlights multiple hydrogen bonds formed between the hydroxyl-rich regions of EGCG and the side chains of ASP28 and ARG27, while additional hydrogen bonding is observed with PRO20 and LEU23, indicating extensive polar complementarity. The planar aromatic rings of EGCG also facilitate van der Waals contacts and potential π-stacking interactions within the hydrophobic patches of the binding pocket. This dense interaction profile underscores EGCG’s high affinity and specificity toward IL-10, suggesting it may effectively modulate IL-10’s immunosuppressive functions. These findings position EGCG as a potent natural candidate for IL-10-targeted therapeutic strategies, especially in inflammation and immune regulation contexts**(Fig. S5)**.
    1.4.7 ***Ellagic acid (-2.983 Kcal/mol):*** The molecular docking analysis of ellagic acid with Interleukin-10 (IL-10), using structural insights from PDB ID: 2ILK, reveals a compact and highly stabilized interaction within the cytokine’s receptor-binding groove. The 3D visualization shows ellagic acid (in green stick form) fitting snugly into a polar and hydrophobic cavity, anchored predominantly by a key hydrogen bond with ARG27, which plays a critical role in ligand orientation. The 2D interaction diagram highlights additional non-covalent interactions, including hydrophobic contacts with LEU23, LEU26, and LEU98, as well as PHE56 and MET68. These residues create a favourable hydrophobic environment that complements the planar polyphenolic structure of ellagic acid. The extensive hydroxylation pattern of ellagic acid allows for multiple hydrogen bond donor/acceptor opportunities, although the primary strong polar contact is with ARG27. This interaction profile suggests that ellagic acid can stably associate with IL-10, potentially influencing its biological activity, and supports its therapeutic promise as a natural modulator of anti-inflammatory cytokine signaling **(Fig. S5)**.
1.5 **Molecular docking with B-RAF—PDB ID:1UWH (25)**
    1.5.1 ***Apigenin (Binding energy: -9.056 Kcal/mol):*** The molecular interaction analysis of Apigenin with B-RAF kinase (PDB ID: 1UWH) reveals a stable binding conformation within the ATP-binding pocket of the kinase, supported by multiple non-covalent interactions with key active site residues. Apigenin is well-anchored in the catalytic cleft, primarily through hydrogen bonding with GLU 500, CYS 531, and GLN 529, which enhance the ligand’s stabilization and specificity. The hydroxyl groups on the flavone scaffold play a critical role in these polar interactions. Additionally, π–π stacking interactions between the aromatic rings of Apigenin and residues such as TRP 530 and PHE 582 further contribute to the stabilization of the ligand within the hydrophobic regions of the binding site. The 3D visualization indicates deep accommodation of Apigenin in the pocket, showing optimal complementarity with the surrounding protein surface, particularly near GLU 500 and CYS 531. The 2D interaction diagram highlights the ligand’s extended conformation and widespread interaction network, including contact with hydrophobic residues like ILE 462, ILE 526, and LEU 504. Overall, these findings suggest that Apigenin binds effectively to B-RAF, demonstrating its potential as a natural kinase modulator for anticancer therapeutic development **(Fig. S6)**.
    1.5.2 **Silymarin (Binding score: -9.132 Kcal/mol)** The interaction analysis of Silymarin with B-RAF kinase (PDB ID: 1UWH) demonstrates a stable and favorable binding conformation within the ATP-binding pocket of the kinase domain. Silymarin forms a network of hydrogen bonds with key residues such as SER 535, ASN 579, GLU 500, SER 534, and PHE 594, which help anchor the ligand deeply within the catalytic cleft. The polyhydroxylated chromone core facilitates additional hydrogen bond donor–acceptor interactions with surrounding polar residues. π–π stacking interactions between the aromatic rings of Silymarin and residues like PHE 594 and TRP 530, along with hydrophobic contacts involving ILE 526, LEU 504, and PHE 582, further stabilize the ligand-protein complex. The 3D visualization highlights Silymarin’s deep accommodation and favorable electrostatic complementarity with the binding site, while the 2D interaction map confirms an extended conformation that optimally spans the active site tunnel. Collectively, these interactions suggest that Silymarin has a strong binding affinity for B-RAF and may serve as a promising natural lead for the development of kinase-targeted anticancer therapeutics(25) **(Fig. S6)**.
    1.5.3 ***Ellagic acid (Binding energy: -9.025 Kcal/mol):*** Ellagic acid exhibits stable binding within the ATP-binding pocket of the B-RAF kinase (PDB ID: 1UWH) through a combination of hydrogen bonding and hydrophobic interactions. The 3D visualization shows the ligand (green sticks) well accommodated in the active site groove, flanked by key residues Thr528, Cys531, and Glu532, which form critical hydrogen bonds with the hydroxyl and carbonyl groups of Ellagic acid. The 2D interaction diagram further highlights additional polar and hydrophobic contacts with residues such as Gln529, Leu513, Trp530, and Ala480, contributing to ligand stabilization. Notably, Lys482 and Glu532 are involved in charge-assisted hydrogen bonding, while non-polar residues like Phe582, Phe594, and Val470 offer π-π stacking and van der Waals interactions. These interactions collectively suggest that Ellagic acid has a strong affinity for the B-RAF binding site, potentially interfering with its kinase activity and downstream signaling, indicating its potential as an anti-cancer agent targeting RAF-MEK-ERK pathway **(Fig. S6)**.
    1.5.4 ***Anthocyanidin* (Binding energy -8.866 kcal/mol):** The interaction analysis of Anthocyanidins with B-RAF kinase (PDB ID: 1UWH) reveals a strong binding affinity within the ATP-binding pocket, primarily stabilized through hydrogen bonding and hydrophobic contacts. The 3D binding pose shows the molecule (in green) occupying the catalytic cleft, forming key hydrogen bonds with Thr528, Gln529, and Cys531, which are critical residues in the B-RAF activation segment. The 2D interaction diagram highlights additional polar interactions with Glu500 and Lys482, where the latter may contribute electrostatic stabilization via a salt bridge. Hydrophobic residues such as Phe582, Trp530, Leu504, Ala480, and Val470 offer π-π stacking and van der Waals interactions, enhancing the ligand’s fit within the pocket. These multiple stabilizing contacts suggest that Anthocyanidins can effectively inhibit B-RAF by interfering with its ATP binding and subsequent kinase activity, highlighting their potential as natural therapeutic agents targeting MAPK signaling in cancer **(Fig. S6)**.
    1.5.5 ***EGCG (Binding Energy: -7.589 Kcal/mol):*** The interaction of Epigallocatechin gallate (EGCG) with B-RAF kinase (PDB ID: 1UWH) reveals a strong and stable binding within the ATP-binding pocket of the kinase. The compound forms multiple hydrogen bonds with key amino acid residues including Cys531, His538, Tyr537, Ser534, Thr528, and Asn579, anchoring the ligand deeply within the active site. Additional polar interactions involve Gly533, Ser535, and Gln529, highlighting EGCG’s ability to establish an extensive hydrogen bonding network due to its multiple hydroxyl groups. Hydrophobic interactions with residues such as Trp530, Phe582, Phe594, Ile462, and Val470 further stabilize the complex. The gallate moiety of EGCG fits into a hydrophobic cleft, while its polyphenolic rings interact with both polar and nonpolar residues, supporting favorable van der Waals contacts and potential π-stacking. These interactions suggest EGCG as a promising B-RAF inhibitor by mimicking ATP binding, potentially disrupting the kinase’s phosphorylation function in oncogenic signaling pathways **(Fig. S6)**.
    1.5.6 ***Allicin (Binding energy -3.780 kcal/mol)***:The binding interaction of Allicin with B-RAF kinase (PDB ID: 1UWH) demonstrates a distinctive covalent or near-covalent interaction pattern. Allicin positions itself deeply within the ATP-binding cleft, forming a key interaction with the thiol group of Cys531, suggesting potential covalent modification via its electrophilic sulfoxide group. The disulfide core of Allicin is stabilized by surrounding residues, including Glu532, Gln529, Trp530, and Thr528, which contribute to polar and van der Waals interactions. Additionally, hydrophobic contacts with residues such as Phe582, Leu513, Ala480, Val470, and Ile462 enhance the stability of the ligand within the pocket. Notably, the sulfinyl group may engage in dipole interactions or weak hydrogen bonding with nearby residues like Glu532. These interactions imply that Allicin can effectively occupy the B-RAF active site and possibly exert inhibitory effects through both covalent and non-covalent mechanisms, making it a potential candidate for B-RAF targeting in anticancer strategies **(Fig. S6)**.
1.6 **Molecular docking with K-RAS—PDB: 8AZX (26)**
    1.6.1 **Apigenin (Binding energy: -7.142 Kcal/mol):** The binding interaction of Apigenin with KRAS (PDB ID: 8AZX) reveals a well-oriented fit within the active site pocket, engaging in several key interactions that stabilize its conformation. Apigenin forms critical hydrogen bonds with Glu63, Tyr64, and Asp69, contributing significantly to its binding affinity. Additional hydrogen bonding occurs with Ser65 and Arg68, as mediated through a water molecule, enhancing the stability of the ligand within the hydrophilic region of the pocket. The aromatic ring of Apigenin is nestled in a hydrophobic sub-pocket formed by residues such as Tyr96, Met72, Val9, and Ile100, allowing for favorable π-π stacking and van der Waals interactions. The combination of polar and hydrophobic interactions, along with the molecule’s ability to form a stable hydrogen-bonding network, underscores its potential as a modulator of KRAS function. These findings highlight Apigenin as a promising candidate for targeting KRAS in cancer therapeutics **(Fig. S7)**.
    1.6.2 ***Quercetin (Binding energy: -5.409 Kcal/mol):*** The interaction profile of Quercetin with KRAS (PDB ID: 8AZX) demonstrates a stable and well-coordinated binding within the protein’s active site, predominantly through hydrogen bonding and hydrophobic interactions. Quercetin forms strong hydrogen bonds with critical polar residues, including Glu62, Asp92, His95, and Tyr96, anchoring the molecule firmly in the pocket. Additional water-mediated hydrogen bonding is observed with Gly60, further enhancing binding stability. The ligand’s polyphenolic scaffold is nestled within a hydrophobic environment composed of residues like Met72, Gln99, and Tyr64, allowing for van der Waals and π-π stacking interactions. The combination of multiple direct and water-bridged hydrogen bonds, along with nonpolar interactions, suggests that Quercetin exhibits high affinity for KRAS, supporting its potential as a natural scaffold for anti-KRAS therapeutic development **(Fig. S7)**.
    1.6.3 ***Silymarin (Binding energy -5.834 Kcal/mol):*** The interaction analysis of Silymarin with KRAS (PDB ID: 8AZX) indicates strong and multifaceted binding within the protein’s active site. Silymarin forms multiple hydrogen bonds with key residues such as Glu63, Tyr64, and Asp69, establishing a robust polar interaction network. Additional stabilization is achieved through water-mediated hydrogen bonds involving Asp57 and His95, enhancing its anchoring within the binding pocket. The planar polyphenolic structure of Silymarin is favorably accommodated in the hydrophobic cavity formed by Val9, Val7, Tyr96, Met72, and Ile100, promoting van der Waals and hydrophobic interactions. The ligand also interacts with Arg68 and Arg102, contributing to its binding specificity. These combined interactions—both polar and nonpolar—suggest that Silymarin has strong affinity for KRAS and may serve as a promising candidate for KRAS-targeted therapeutic development **(Fig. S7)**.
    1.6.4 ***Anthocyanidins (Binding energy = -5.133 Kcal/mol):*** The binding interaction of Anthocyanidins with KRAS (PDB ID: 8AZX) reveals a strong and specific engagement within the protein’s active site, characterized by multiple hydrogen bonds and polar contacts. The ligand forms key hydrogen bonds with Glu62, His95, and Asp92, which are crucial residues in the KRAS switch regions, potentially disrupting its function. A notable electrostatic interaction is observed between the negatively charged oxygen on the anthocyanidin scaffold and the protonated side chain of His95, contributing to enhanced stability. Water-mediated interactions further support binding, especially in coordination with Glu62 and Asp92. Additionally, hydrophobic residues such as Tyr64, Tyr96, and Met72 flank the ligand, providing π-π and van der Waals interactions that reinforce the overall binding affinity. This interaction profile suggests that anthocyanidins are well-positioned to act as allosteric modulators of KRAS, making them promising candidates for further development in anti-KRAS therapeutic strategies **(Fig. S7)**.
    1.6.5 ***Allicin (Binding energy = -3.850 Kcal/mol):*** Allicin exhibits specific binding interactions with KRAS (PDB ID: 8AZX) by engaging key residues within the switch II groove of the protein. The ligand forms a critical hydrogen bond with Glu63, contributing significantly to the stability of the ligand–protein complex. The sulfur-containing functional groups of allicin are nestled within a largely hydrophobic pocket lined by residues such as Val9, Val103, Ile100, and Tyr96, facilitating van der Waals and hydrophobic interactions. Additionally, proximity to polar and charged residues like Glu62, Arg68, and Asp69 supports potential electrostatic interactions that further anchor the ligand. The interaction with Tyr64 and His95 may also influence local conformational dynamics, possibly interfering with KRAS activation or effector binding. Overall, the binding profile of allicin suggests a well-oriented interaction within a regulatory site of KRAS, indicative of its potential as a natural covalent or non-covalent modulator of KRAS function **(Fig. S7)**.
    1.6.6 ***EGCG (Binding energy -2.418 Kcal/mol):*** The interaction of Epigallocatechin gallate (EGCG) with KRAS (PDB: 8AZX) reveals a dense network of hydrogen bonds and polar interactions, positioning EGCG as a strong binder within the KRAS pocket. The 3D molecular docking image (left) shows EGCG (in green) engaging deeply with the binding cleft, primarily through its multiple hydroxyl groups. Key residues involved include GLU62, HIE95, LYS88, and TYR64, as confirmed by the 2D interaction map (right), where EGCG forms hydrogen bonds with GLU62, LYS88, and HIE95. The gallate and catechin moieties contribute several polar contacts, anchoring the molecule via extensive hydrogen bonding and possible π–π stacking or hydrophobic interactions with the surrounding aromatic residues like TYR96. These interactions suggest that EGCG effectively occupies the binding site, potentially interfering with KRAS function and signaling pathways, thereby supporting its role as a promising natural inhibitor in anti-cancer therapy **(Fig. S7)**.
    1.6.7 ***Ellagic acid (Binding energy -3.674 Kcal/mol***): The interaction of ellagic acid with KRAS (PDB: 8AZX) involves key hydrogen bonding and polar contacts with several amino acid residues within the binding pocket. The molecular docking visualization (left panel) shows ellagic acid (in green) nestled in a surface-exposed pocket surrounded by polar residues, notably GLU62, LYS88, and HIE95. These interactions are further clarified in the 2D interaction diagram (right panel), which indicates that ellagic acid forms strong hydrogen bonds through its hydroxyl and carbonyl groups with GLU62 and LYS88, and interacts with HIE95 and TYR96. The hydroxyl groups of ellagic acid are crucial for these interactions, stabilizing the compound within the KRAS binding site and potentially disrupting KRAS activity. These observations suggest that ellagic acid may function as a KRAS modulator by binding at a key regulatory site, contributing to its potential as an anti-cancer agent **(Fig. S7)**.

**Reference**

1.	Peixoto Araujo NM, Arruda HS, dos Santos FN, de Morais DR, Pereira GA, Pastore GM. LC-MS/MS screening and identification of bioactive compounds in leaves, pulp and seed from Eugenia calycina Cambess. Food Research International. 2020;137:109556.

2.	Cicek M, Unsal V, Emre A, Doganer A. Investigation of the Effects of Apigenin, a Possible Therapeutic Agent, on Cytotoxic and SWH Pathway in Colorectal Cancer (HT29) Cells. Adv Pharm Bull. 2023;13(1):188-95.

3.	Ruela-de-Sousa RR, Fuhler GM, Blom N, Ferreira CV, Aoyama H, Peppelenbosch MP. Cytotoxicity of apigenin on leukemia cell lines: implications for prevention and therapy. Cell Death Dis. 2010;1(1):e19.

4.	Sun D, Zou Y, Song L, Han S, Yang H, Chu D, et al. A cyclodextrin-based nanoformulation achieves co-delivery of ginsenoside Rg3 and quercetin for chemo-immunotherapy in colorectal cancer. Acta Pharm Sin B. 2022;12(1):378-93.

5.	Sameri S, Mohammadi C, Mehrabani M, Najafi R. Targeting the hallmarks of cancer: the effects of silibinin on proliferation, cell death, angiogenesis, and migration in colorectal cancer. BMC Complement Med Ther. 2021;21(1):160.

6.	Mazewski C, Liang K, Gonzalez de Mejia E. Comparison of the effect of chemical composition of anthocyanin-rich plant extracts on colon cancer cell proliferation and their potential mechanism of action using in vitro, in silico, and biochemical assays. Food Chem. 2018;242:378-88.

7.	Sarvizadeh M, Hasanpour O, Naderi Ghale-Noie Z, Mollazadeh S, Rezaei M, Pourghadamyari H, et al. Allicin and Digestive System Cancers: From Chemical Structure to Its Therapeutic Opportunities. Front Oncol. 2021;11:650256.

8.	La X, Zhang L, Li Z, Li H, Yang Y. (โ่ั)-Epigallocatechin Gallate (EGCG) Enhances the Sensitivity of Colorectal Cancer Cells to 5-FU by Inhibiting GRP78/NF-ฮบB/miR-155-5p/MDR1 Pathway. Journal of Agricultural and Food Chemistry. 2019;67(9):2510-8.

9.	Yousuf M, Shamsi A, Khan P, Shahbaaz M, AlAjmi MF, Hussain A, et al. Ellagic Acid Controls Cell Proliferation and Induces Apoptosis in Breast Cancer Cells via Inhibition of Cyclin-Dependent Kinase 6. Int J Mol Sci. 2020;21(10).

10.	Agriculture USDo. Garlic (FDC ID:1104647) USA: U.S. Department of Agriculture; 2019 [Available from: <https://fdc.nal.usda.gov/food-details/1104647/nutrients>.

11.	Agriculture USDo. Apples, FDC ID:1750340 USA: U.S. Department of Agriculture; 2019 [Available from: <https://fdc.nal.usda.gov/food-details/1750340/nutrients>.

12.	Elhassaneen YA, Nasef AZ, Arafa RS, Bayomi AI. Bioactive compounds and antioxidant activities of milk thistle (Silybum marianum) extract and their potential roles in the prevention of diet-induced obesity complications. American Journal of Food Science and Technology. 2023;11(3):70-85.

13.	Central UF. Parsley, fresh, 100 g USA: USDA FoodData Central; 2019 [Available from: <https://growables.com/informationVeg/documents/ParsleyFreshUsda.pdf?utm_source=chatgpt.com>.

14.	Tarek K, Farid A, Safwat G. Extraction of grape seeds by different solvents affects the activities of the resultant extract. AMB Express. 2025;15(1):51.

15.	Commodites C. Product Technical Dossier (Green Tea Extract 40% Polyphenols ) England: Cambridge Commodites; 2026 [Available from: <https://www.cambridgecommodities.com/ugc-1/pdfs/spec-0736.pdf?utm_source=chatgpt.com>.

16.	Agriculture USDo. Pomegranates, raw (Nutrients),FDC ID:169134 U.S. Department of Agriculture: USA; 2019 [Available from: <https://fdc.nal.usda.gov/food-details/169134/nutrients>.

17.	Czabotar PE, Garcia-Saez AJ. Mechanisms of BCL-2 family proteins in mitochondrial apoptosis. Nature reviews Molecular cell biology. 2023;24(10):732-48.

18.	Korsmeyer S, Wei M, Saito M, Weiler S, Oh K, Schlesinger P. Pro-apoptotic cascade activates BID, which oligomerizes BAK or BAX into pores that result in the release of cytochrome c. Cell Death & Differentiation. 2000;7(12):1166-73.

19.	Tahmasebi S, Alimohammadi M, Khorasani S, Rezaei N. Pro-tumorigenic and Anti-tumorigenic Roles of Pro-inflammatory Cytokines in Cancer. Cancer Immunology: The Immune System and Tumor: Springer; 2025. p. 529-53.

20.	Bahar ME, Kim HJ, Kim DR. Targeting the RAS/RAF/MAPK pathway for cancer therapy: from mechanism to clinical studies. Signal transduction and targeted therapy. 2023;8(1):455.

21.	Do Carmo AL, Bettanin F, Oliveira Almeida M, Pantaleão SQ, Rodrigues T, Homem-de-Mello P, et al. Competition between phenothiazines and BH3 peptide for the binding site of the antiapoptotic BCL-2 protein. Frontiers in chemistry. 2020;8:235.

22.	Dengler MA, Robin AY, Gibson L, Li MX, Sandow JJ, Iyer S, et al. BAX Activation: Mutations Near Its Proposed Non-canonical BH3 Binding Site Reveal Allosteric Changes Controlling Mitochondrial Association. Cell Rep. 2019;27(2):359-73.e6.

23.	Somers W, Stahl M, Seehra JS. 1.9 Å crystal structure of interleukin 6: implications for a novel mode of receptor dimerization and signaling. The EMBO journal. 1997.

24.	Zdanov A, Schalk-Hihi C, Wlodawer A. Crystal structure of human interleukin-10 at 1.6 A resolution and a model of a complex with its soluble receptor. Protein Sci. 1996;5(10):1955-62.

25.	Wan PT, Garnett MJ, Roe SM, Lee S, Niculescu-Duvaz D, Good VM, et al. Mechanism of activation of the RAF-ERK signaling pathway by oncogenic mutations of B-RAF. Cell. 2004;116(6):855-67.

26.	Kim D, Herdeis L, Rudolph D, Zhao Y, Böttcher J, Vides A, et al. Pan-KRAS inhibitor disables oncogenic signalling and tumour growth. Nature. 2023;619(7968):160-6.
